# To PLP or Not to PLP: Stereodivergent Transaminase-Catalyzed Reactions Directed by Kinetic and Thermodynamic Control

**DOI:** 10.1021/acs.joc.5c01382

**Published:** 2025-09-01

**Authors:** Madeline J. Fitzgerald, Xiaoyan Li, Dawei Peng, Chenlu Qin, Yuehan Sun, Chloe C. Wang, YeePui Yeung, Chi Nguyen, Hanna M. Key

**Affiliations:** † Department of Chemistry, 2813 Davidson College, Davidson, North Carolina 28036, United States; ‡ Department of Chemistry, University of California, Berkeley, California 94720-1460, United States; § Department of Chemistry, 1259 University of Michigan, 930 N University Ave, Ann Arbor, Michigan 48109, United States; ∥ Department of Biostatistics, 50296 Yale School of Public Health, 60 College Street, New Haven, Connecticut 06520-0834, United States; ⊥ Center of Molecular and Cellular Oncology, Yale School of Medicine, 300 George Street, Suite 6400, New Haven, Connecticut 06511, United States; # Department of Radiation Oncology 609772 Duke University Medical Center. Duke Cancer Center, Medicine Circle, Durham, North Carolina 27710, United States; ∇ Center for Immunology and Inflammatory Diseases, 2348 Massachusetts General Hospital, 55 Fruit Street, Boston, Massachusetts 02114, United States; ○ Division of Rheumatology, Allergy, and Immunology, Massachusetts General Hospital, 55 Fruit Street, Boston, Massachusetts 02114, United States

## Abstract

Selective catalysis is a key objective in organic synthesis, and reactions with differing kinetic and thermodynamic products present the opportunity for divergent reaction outcomes with a single catalyst. We report a biocatalytic method in which a single transaminase can form either *cis* or *trans* cyclohexylamines in high diastereoselectivity. With the model substrate 4-methylcyclohexanone, *E. coli* cells expressing WT-*Vf*-ATA form either diastereomer of the amine product in >10:1 dr and >70% conversion, depending on reaction conditions, stereodivergence that also extends to a range of substrates. In the case of α(2)-substituted ketones, a concurrent dynamic kinetic resolution enables conversion of racemic ketones into *cis* or *trans* amines that are enantiomerically and diastereomerically enriched. Supplementation (or not) of the PLP cofactor in the reaction was found to be key in directing the stereochemical outcome and informed the development of a modified preparation of cells expressing the transaminases that included a metabolic precursor to the PLP cofactor, resulting in cells with more holo ATA catalyst and higher catalytic activity. This research demonstrates a rare, but operationally simple, example of highly stereodivergent reactions effected by a single catalyst and sheds new light on the PLP cofactor as a handle to optimize biocatalysis by transaminases.

## Graphical abstract

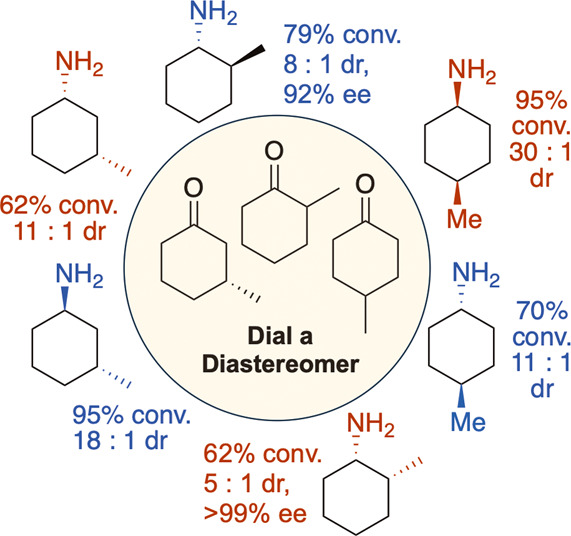

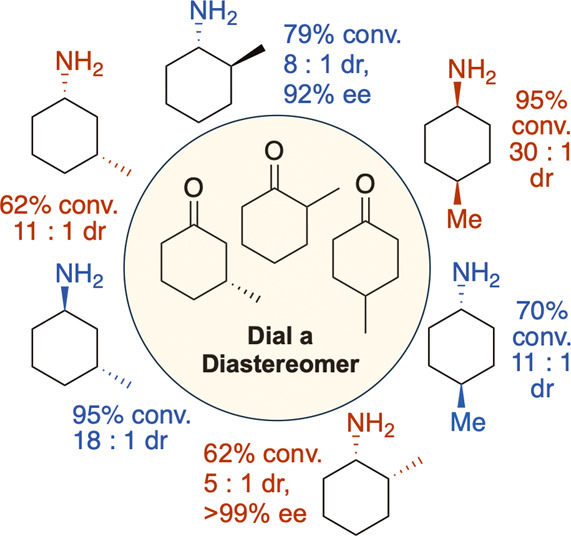

## 1 Introduction

Selective catalysis is a key tool for efficiency in organic chemistry and is underpinned by both kinetic and thermodynamic effects. Transition state barriers determine which of multiple products form the fastest, whereas the relative energies of the products determine which is thermodynamically favored. Differentiated kinetic and thermodynamic control becomes particularly interesting when one product is kinetically favored, while a different product is thermodynamically favored (Figure 1A). This scenario potentially allows a single catalyst to produce either of two products depending on the reaction conditions. Although an omnipresent concept in organic chemistry, it is an outcome that, in practice, is rarely achieved in full.^1–4^ The Diels–Alder^5–10^ and aldol^1,2^ reactions are among examples where reaction conditions such as shorter reaction times and lower reaction temperatures may lead to a kinetic isomer, while higher temperatures, longer reaction times, and/or higher catalyst loading may afford thermodynamic isomers. In select cases, this has been achieved (Figure 1B). Despite its theoretical simplicity, the need for catalysts that both facilitate the reaction reversibly and have kinetic preference for a nonthermodynamic product are among the challenges to designing selective reactions according to these principles.

**Figure 1:**
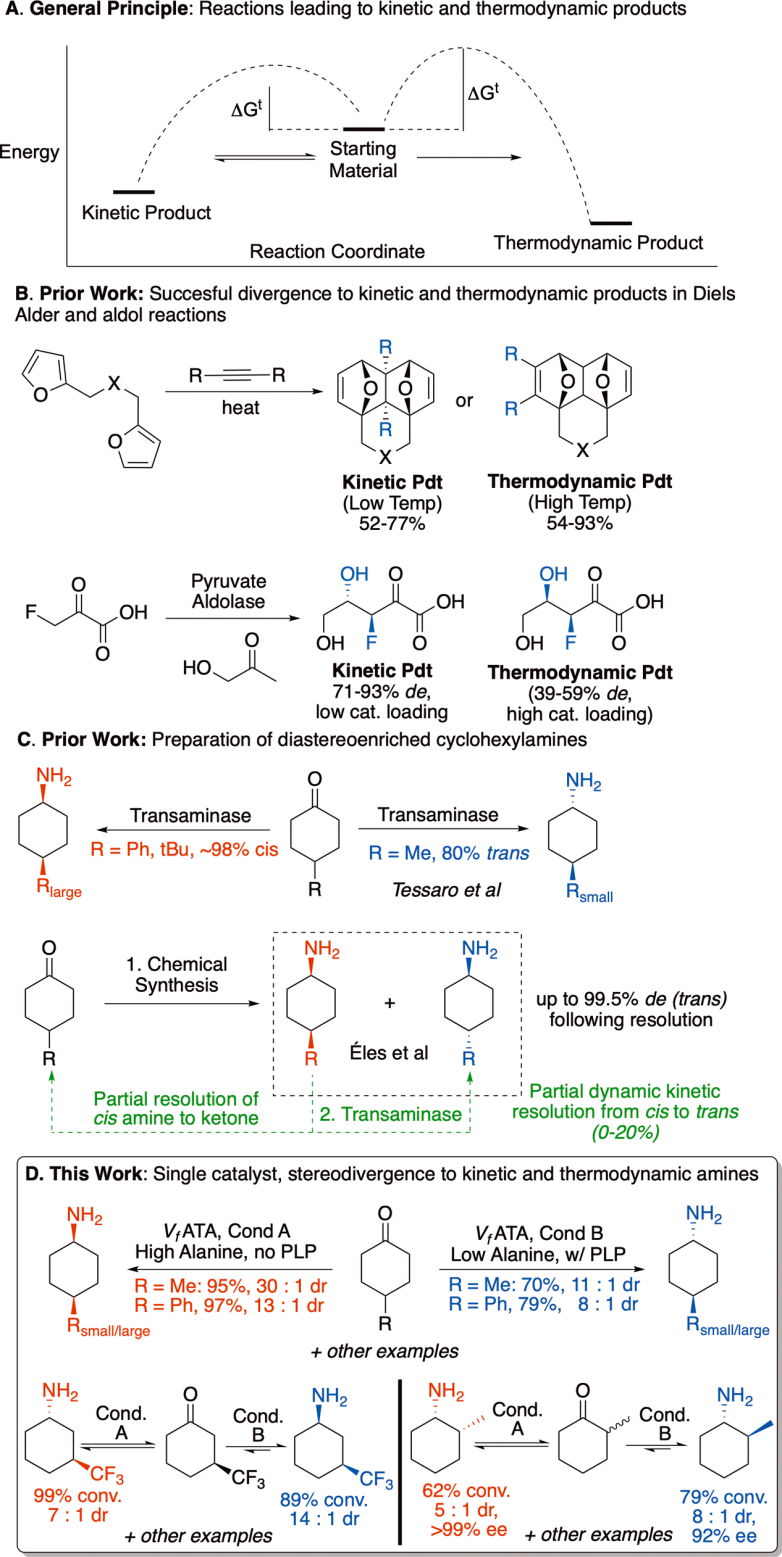
Divergent reaction outcomes due to kinetic and thermodynamic products. (A) Energetic conditions leading to differing kinetic and thermodynamic products. (B) Previous examples of reactions leading to differing products under conditions favoring kinetic and thermodynamic outcomes. Top: Diels–Alder Reaction. Reproduced from [4] Copyright [2018] American Chemical Society. Bottom: Aldol reaction.^1^ (C) Prior work investigating the reaction of cyclohexanones and cyclohexylamines with ATAs.^19,20^ (D) Summary of this work, in which a single transaminase catalyst converts a ketone into either of two stereoisomers with high diastereomer and/or enantiomer selectivity.

Enzymes are known to catalyze a range of reactions in both directions, suggesting their potential for the aforementioned divergent reaction paradigm. Transaminases (ATAs) in particular are a class of enzymes with significant synthetic value, owing to their potential to form stereodefined amines, which are useful building blocks for pharmaceuticals.^11–15^ While transaminases are most often applied in enantiomer-selective reactions, forming products with equal energy, they have also been studied in diastereomer-selective reactions, which result in products of differing energy and therefore potential for kinetic and thermodynamic products. For example, transaminases have been applied in the synthesis of stereodefined, 4-substituted cyclohexylamines from cyclohexanones. Cyclohexylamines form the core of biologically active compounds and known pharmaceuticals, including Ambroxol,^16^ Neltenexine,^17^ and Cariprazine.^18^ The *trans*-amine products of such reactions should be thermodynamically favored, owing to the diequatorial placement of both substituents in the chair conformation. Previous research, outlined next, also suggests that the *cis* product, at least for some substrates, may be kinetically favored.

For example, Tessaro et al. showed that various ATAs, including *Vibrio fluvialis* (*V_f_-*ATA), catalyze substrate-controlled reactions of 4-substituted cyclohexanones, observing *cis* products in cases of sterically hindered R groups (^t^Bu, Ph, ∼98% *cis*) and the *trans* product in the case of 4-methylcyclohexanone (up to 80% *trans*).^19^ More recently, Éles et al. reported that a variety of *(R)*- and *(S)-*selective transaminases react with cyclohexanones bearing four different sterically hindering 4-substituents, with selectivity generally favoring *cis* products, although in a singular case, a mutant *Ar­(R)-*ATA displayed moderate preference for the *trans* product from 4-benzylcyclohexanone.^20^ These authors then showed that mixtures of *cis*- and *trans*-cyclohexylamines (produced by a chemical reductive amination reaction) can be resolved kinetically by immobilized ATAs in flow reactors, yielding nearly diastereopure *trans* amines for four substrates.^20^

Through this work, the authors observed that, in addition to the reconsumption of the *cis* amine to ketone, there was some degree of dynamic isomerization, with the mole fraction of the *trans* product increasing by up to 20% during the reaction, depending on the substrate. This work suggests that when a mixture of amines is generated by a prior reaction, a transaminase can both selectively convert the *cis* amine to the ketone (kinetic resolution) and then may also convert some of that ketone into the thermodynamically favored *trans* amine product.

Together, these findings, conducted prior to and concurrently with our studies described here, suggest the potential to implement a single catalyst in the one-pot conversion of a cyclohexanone into either its *cis* (kinetic) or *trans* (thermodynamic) amine product. To achieve such a reaction paradigm, pseudo-irreversible reaction conditions (such as using an excess amine donor) could lead to the kinetically favored *cis* product. On the other hand, reversible reaction conditions (maintained, for example, by using a smaller excess of amine donor) might initially give rise to the *cis* product but eventually reach equilibrium, in which the *trans* product predominates.

Here, we report the one-pot, one-catalyst, diastereodivergent conversion of ketones into pseudo-asymmetrical *cis* or *trans* amines catalyzed by whole cells expressing the transaminase from*Vibrio fluvialis* (*V*_*f*_-ATA).^21–27^ Our findings show that manipulation of the concentrations of the alanine amine donor, and more unexpectedly, the PLP cofactor, enables reversal of the selectivity of the reaction of 4-methylcyclohexanone from 95% conversion and >95% *cis* product to 70% conversion and >90% *trans* product (Figure 1C). This phenomenon readily extends to a range of 4-substituted cyclohexanones with both small and large substituents, furnishing *cis* and *trans* diastereomers with high selectivity for both isomers. Several enantioenriched, 3-substituted cyclohexanones were also found to react in the same reaction paradigm, leading to *cis* or *trans* isomers of the products depending on reaction conditions (Figure 1D). Moreover, in the case of 2-substituted cyclohexanones, we found that racemic substrates can be submitted to the reaction, and enantioenriched samples of either *cis* or *trans* cyclohexyl-1­(*S*)-amine products can be formed (Figure 1D). In a secondary finding, supplementing the cells expressing transaminases with pyridoxine (PYP) was found to increase the effectiveness of those cells in whole-cell catalysis, enabling more rapid equilibration of sterically hindered substrates to thermodynamic products.

## 2 Results and Discussion

### 2.1 Diastereodivergent Synthesis of 4-Substituted Cyclohexylamines

To investigate the potential for transaminases to catalyze diastereodivergent reactions, we started with the model reaction of 4-methylcyclohexanone (**1A**), catalyzed by whole cells expressing the wild-type ATA from*Vibrio fluvialis* (*Vf*) (Figure 2A). Beyond studies pertaining to cyclohexanones and cyclohexylamines, *V*_*f*_-ATA^21–26,28^ is a widely studied transaminase for which there is immense data on its structure, reactivity, and mutability, which made it an attractive choice as we initiated our studies. Typical conditions for transaminase-catalyzed reactions of ketones include an excess of alanine (or other amine donor) along with a catalytic amount of exogenous PLP cofactor (Figure 2A). Under these conditions, we found that the reaction of 4-methylcyclohexanone in the presence of BL21­(DE3) *E. coli* cells expressing wild-type *V*_*f*_-ATA afforded the amine products **1B** and **1C** in >90% conversion and low diastereomer selectivity (∼2:1 *trans*:*cis*).

**Figure 2:**
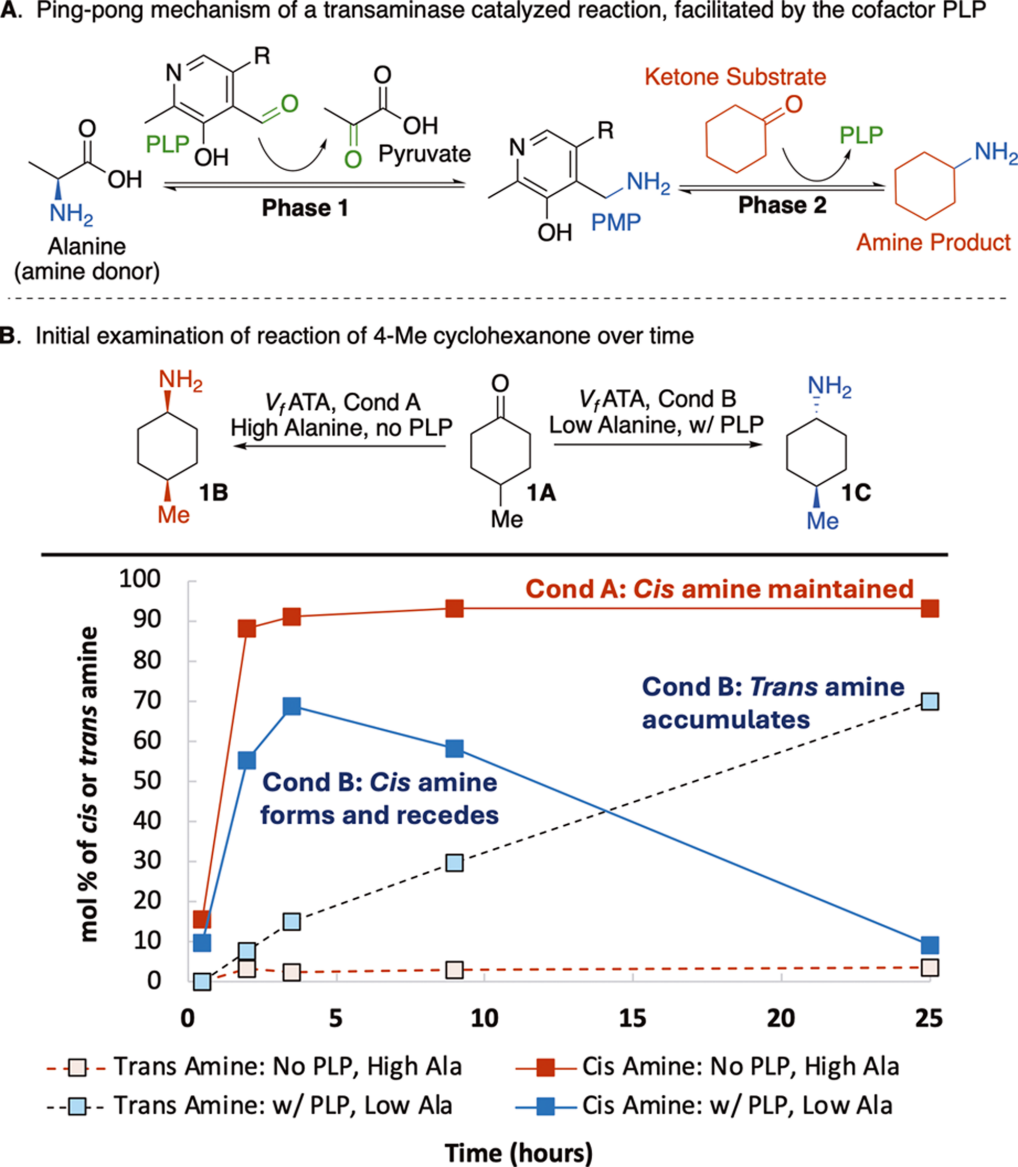
Initial study of the effect of PLP cofactor and alanine loading on the diastereomer selectivity of a transaminase-catalyzed reaction. (A) Overview of the mechanism of a transaminase-catalyzed reaction, including the involvement of the PLP cofactor as an amine transfer agent. R = CH_2_OPO_3_^2–^ (B) Model reaction used to study the effects of [PLP] and [alanine] on the reaction outcome. No PLP = no exogenous PLP added. w/PLP = including 1 mM PLP. Low Ala = 2.5 eq, High Ala = 30 eq. Plot of the mol % of *cis* and *trans* amines present in the reaction over 24 h, quantified by GC. mol % = (*cis* or *trans* amine/(*cis* amine + *trans* amine + ketone) × 100).

Considering these initial results, we hypothesized that using a smaller excess of alanine might increase the reversibility of the reaction toward the thermodynamic product. The expected impact of the [PLP] was less clear. PLP functions as a catalytic cofactor as well as a structural cofactor that binds at the interface of the dimeric enzyme; higher [PLP] may increase the concentration of holo (active) catalytic sites and/or dimeric transaminase structures,^26,28–30^ which could lead to more effective equilibration to the thermodynamic product through higher rates of reaction and/or longer catalyst lifetime. Conversely, reducing, or even omitting, PLP might help trap the reaction at the kinetic product. Thus, we evaluated the outcome of a series of reactions using varied concentrations of PLP and alanine over time to elucidate the effects of these two variables.

The outcomes of experiments reflecting the two extremes of these conditions (either 30 eq. alanine with no supplemental PLP or 2.5 eq. alanine with 0.1 eq. PLP) are shown in Figure 2B. Initially, both conditions produced *cis* amine. However, the reaction with high [alanine] and no exogenous PLP retained the *cis* amine for 24 h, whereas the distribution of compounds in the reaction with less alanine and supplemental PLP shifted from ∼70% *cis* amine to ∼70% *trans* amine over 24 h. When only the amount of either alanine or PLP was changed, equilibration to the *trans* product was also observed in both cases, but at a slower rate. These results suggested that [alanine] and [PLP] are key factors in the dynamic reaction and that their levels alone may be sufficient to direct a single catalyst to form either the *cis* or *trans* product selectivity.

From these initial findings, we sought to optimize the outcome of the reaction toward each diastereomer. Additional studies on the reaction time, buffer, pH, and eq alanine revealed that when using 30 eq. of alanine and a reaction buffer of 100 mM NaPi, pH = 8.0, *cis*-**1B** formed in 95% conversion and 30:1 dr (Figure 3). Moreover, these same reaction conditions (now referred to as “kinetic” conditions) enabled reactions of a series of ketones **1A-9A** to proceed efficiently with high conversion and with selectivity for the *cis* diastereomer (Figure 3). While previous studies found substrate-dependent selectivities (substrates with small R groups leading to *trans* products and substrates with large R groups leading to *cis* products),^19,20^ the kinetic conditions reported here enable ketones with substituents from Me to Ph to all be converted to the *cis* product with high selectivity. Reactions using 0.25–1.0 mmol ketone occurred similarly to those on an analytical scale, and the products could be isolated from the whole-cell reactions in 73–79% yield. Isolated yields from 0.25 mmol scale reactions producing volatile amines, such as **9B**, were lower than those producing the same amines on a 1.0 mmol scale (79% versus 56%) due to modest product losses while evaporating the solvent.

**Figure 3:**
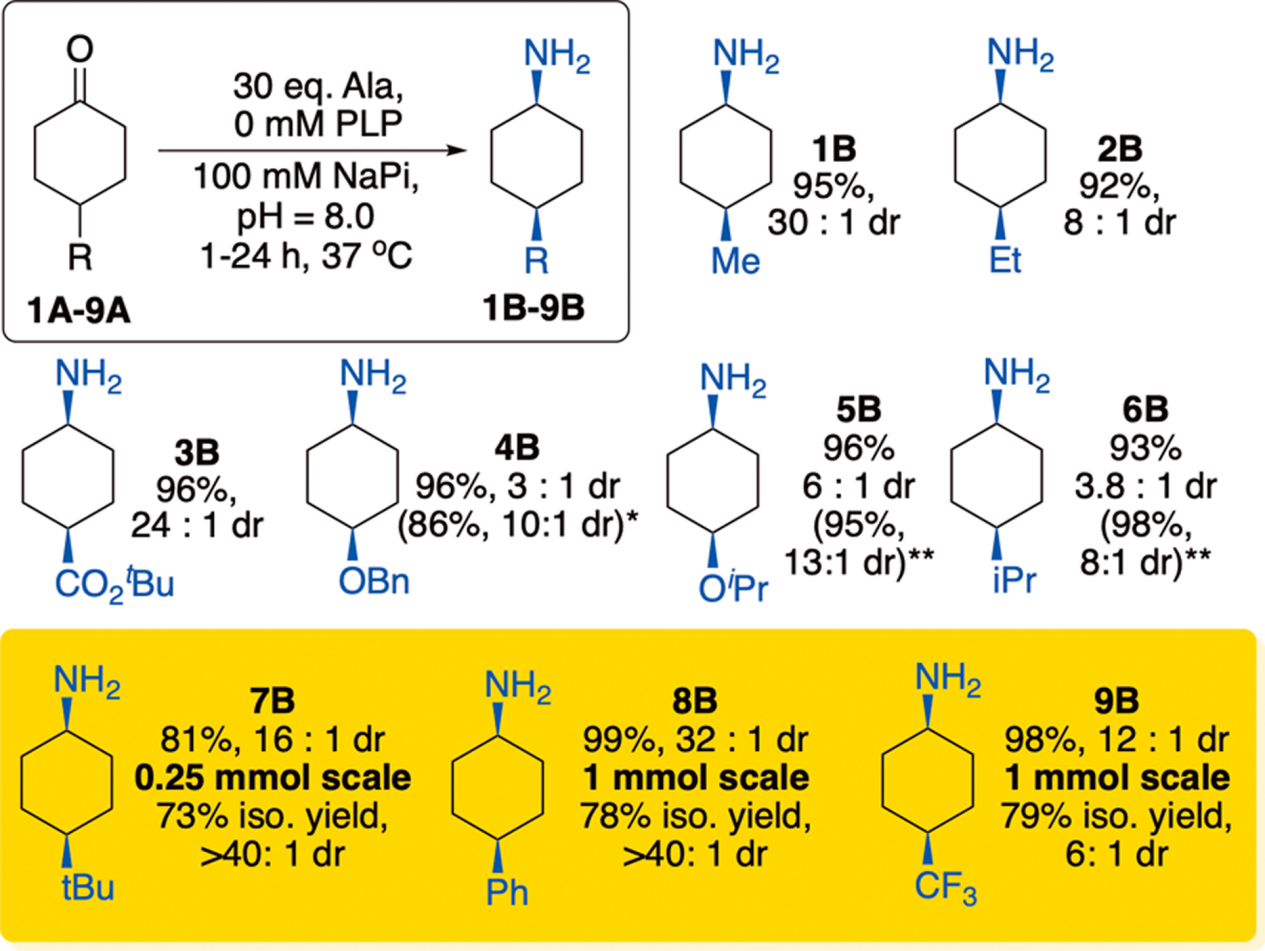
Synthesis of *cis*-configured cyclohexylamines **1B-9B** with varied substitutes at the 4-position. Reactions were conducted using cells expressing WT-*V_f_*-ATA (2 mL reaction containing 10 mM ketone, 300 mM alanine, 0 mM PLP, 80 mg whole cells expressing WT *V*_*f*_-ATA). Conversion and *dr* determined by gas chromatography. *dr* = *cis*:*trans* ratio. *Cells were expressed with PYP (pyridoxine) in the cell culture media. **Outcome using I259V mutant.

With effective conditions in hand to favor the kinetic products, we turned to optimizing the reaction toward the thermodynamic/*trans* isomers. Using the same buffer, but with just 2.5 eq. of alanine and the inclusion of 0.1 eq. of PLP, **1A** reacted with 70% conversion and 11:1 dr favoring the *trans* **1C** product, a near-complete reversal of the selectivity from the kinetic conditions (Figure 4A).

**Figure 4:**
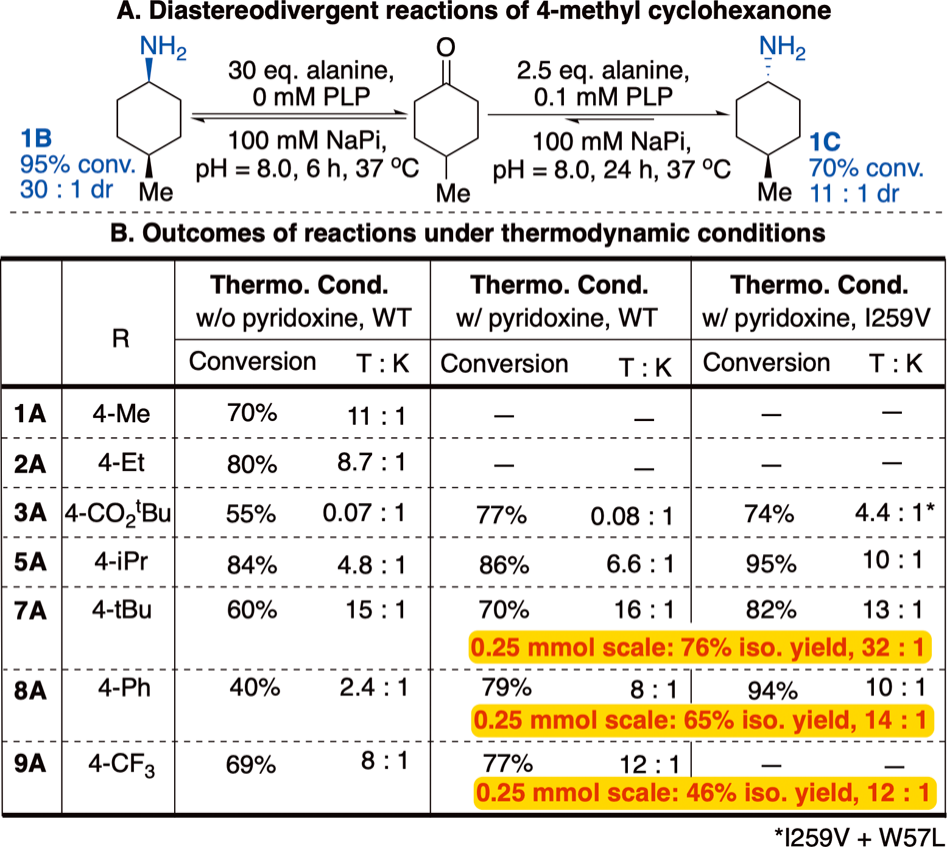
Synthesis of *trans*-cyclohexylamines. (A) Comparison of conditions leading to *cis* (K) and *trans* (T) amines. (B) Outcomes of reactions under thermodynamic-favoring conditions. Reactions were conducted using cells expressing WT-*V_f-_* ATA (2 mL reaction containing 10 mM ketone, 25 mM alanine, 1 mM PLP, 80 mg whole cells expressing WT or I259V *V*_*f*_-ATA). Conversion and *dr* determined by gas chromatography. T:K = thermodynamic (*trans*):kinetic (*cis*) ratio. Select reactions were compared when cells expressing the ATA were cultured with and without PYP, a metabolic precursor to PLP, or when using a mutated transaminase.

Despite the more modest excess of alanine, the conversion remained reasonably high, which we hypothesized to be due to the consumption of pyruvate by cellular metabolism, drawing the reaction to the products side (Figure 2A). Under the same reaction conditions, several other 4-substituted cyclohexanones also reacted effectively, resulting in a high fraction of the *trans* product. However, a few substrates, such as 4-CO_2_^t^Bu (**3**), 4-^i^Pr (**5**), 4-Ph (**8**) reacted more slowly, resulting in lower conversion and/or a lower diastereomer selectivity for the *trans* product; ketone **3** was not observed to favor the *trans* product under these reaction conditions.

### 2.2 The Key Role of PLP in Diastereodivergence

Seeing the critical role of PLP in the reaction dynamics, and given previous research suggesting the importance of the interface-bound PLP to the structure, dimerization, and stability of transaminases,^21,26,28–30^ we considered that the activity of the whole cells might be limited by the [PLP] produced naturally by the*E. coli* cells during the overexpression of the transaminase. If protein expression outpaces PLP biosynthesis, production of apo or partially apo ATA monomers or dimers that are unstable or insoluble may reduce the yield of enzyme per cell, even if PLP is added exogenously after the expression to backfill apo enzymes. To increase the availability of cellular PLP during the expression of the transaminase, we cultured the cells in the presence of 1.7 mM PYP, the hydroxyl metabolic precursor to PLP. When using cells cultured with PYP in catalytic reactions of **1A**, we observed the reactions equilibrated more quickly to the thermodynamic product (Figure 5). Furthermore, when the reactions were conducted in vials with air-permeable membrane closures, rather than airtight caps, the reactions also equilibrated to the thermodynamic product more quickly, further indicative of the importance of aerobic pyruvate metabolism in reaction dynamics.

**Figure 5:**
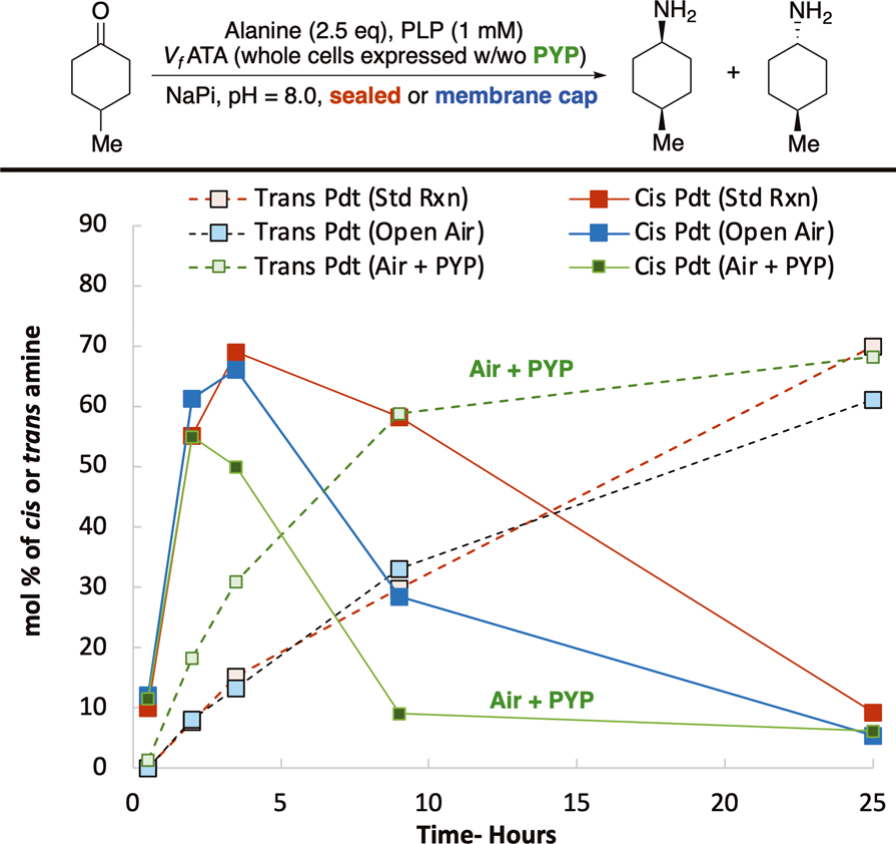
Comparison of reactions catalyzed by *V*_*f*_-ATA cells expressed with or without 1.7 mM PYP in culture media and when using airtight plastic caps or air-permeable membrane. mol % = (*cis* or *trans* amine/(*cis* amine + *trans* amine + ketone) × 100). Values determined by GC.

Seeing these results, we used PYP-supplemented cells in reactions of sterically hindered substrates under the thermodynamic conditions and achieved higher conversions and higher *trans*:*cis* ratios (Figure 4B) than in the reactions using cells expressed without PYP. For example, the *dr* of the product from the 4-phenyl substrate improved from 2:1 to 8:1 *trans* to *cis*. These data show that the singular WT *V*_*f*_-ATA can effectively catalyze one-pot conversion of sterically varied ketones into either *cis* or *trans* products in high selectivity.

However, we also wondered whether mutants of the enzyme could better facilitate the *cis*-to-*trans* isomerization process. Previous studies have shown that *V*_*f*_-ATA is amenable to mutation at a number of sites in the large and small binding pockets, with mutants displaying higher selectivities or reaction rates.^14,21,23,25,26^ In the presence of PYP, we expressed a library of ATA variants with mutations to positions F19, L56, W57, V153, and I259. I259V was found to be a highly effective mutation, without or in combination with the mutation W57L, for increasing the total amine formed and/or the final diastereomer selectivity that was attained (Figure 4). For example, the 4-CO_2_^t^Bu substrate reversed selectivity from 0.08:1 *trans*:*cis* to 4.4:1 *trans*:*cis* when using I259V/W57L as opposed to WT ATA.

For other substrates, conversions and/or selectivities also increased with the mutant I259V, without further improvement when adding W57L. In 0.25 mmol scale reactions of **7A**-**9A**, conducted in round-bottom flasks with air-permeable covers, we observed that equilibration to the thermodynamic product occurred as or more readily than in analytical-scale reactions, with all reactions reaching the same or higher dr. Modest loss of the more volatile **9A** resulted in the only isolated yield below 50%.

### 2.3 Diastereodivergent Synthesis of 3-R-Cyclohexylamines

Given this success in producing diastereomerically enriched 4-substituted cyclohexylamines, we considered how other cyclohexanones would react under the same sets of reaction conditions. First, we examined 3-substituted cyclohexanones, which contain an existing, nonepimerizable stereocenter (Figure 6). As such, each enantiomer of the ketone can undergo an independent, diastereomer-selective reaction with one *cis* and one *trans* isomer possible. Previous research has shown that purified WT *V*_*f*_-ATA in the presence of 20 eq. of alanine and 1 mol % PLP (a hybrid of our kinetic and thermodynamic conditions) leads to a roughly equal mixture of *cis* and *trans* amines from (*S*)-3-methylcyclohexanone, while divergent mutagenesis of the transaminase gives rise to mutants that favor each diastereomer.^22^ In the case of (*R*)-3-methylcyclohexanone, the *trans* product was favored by both WT and all mutated transaminase.^22^ Given that enantioenriched 3-substituted cyclohexanones can be prepared by methods such as asymmetric conjugate addition,^31–37^ further stereoselective elaboration of these types of compounds could be synthetically valuable, and we wondered if *cis* and *trans* diastereomers could be formed from a single catalyst, without the need to customize the catalyst for each targeted diastereomer.

**Figure 6:**
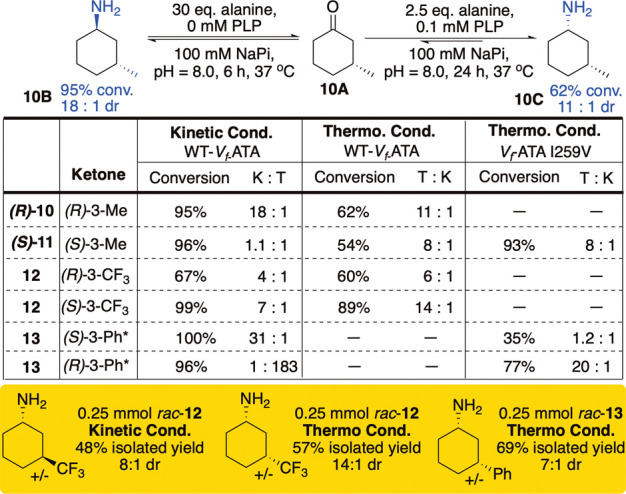
Synthesis of *cis* and *trans* 3-substituted cyclohexylamines. Conversion and selectivity were quantified by GC. K:T or T:K = ratio of thermodynamic (*trans*) to kinetic (*cis*) product in the order indicated. *Cells were expressed with PYP in the cell culture media.

The change to a 3-substituent introduces key differences to our previous reaction paradigm. First, the bis-equatorial (thermodynamic) product is *cis*, rather than *trans*, in configuration. Second, the lack of molecular symmetry in the substrate means the amine is introduced as a true, rather than pseudo, stereocenter in either *R* or *S* configuration. Depending on the configuration at the 3-position, the *cis* (thermodynamic) product may have ( *1R* , *3S*) or ( *1S* , *3R*) stereochemistry, meaning the thermodynamically favored configuration of the 1-amine stereocenter may match or mismatch the natural stereochemical preference of the (*S*)-selective *V*_*f*_-ATA.

As a model substrate, we studied the reaction of the enantiopure (*R*)-3-methylcyclohexanone **(R)-11**. Under the previously kinetic conditions, **(** *R* **)-11** did indeed form the *trans* (*1R*, *3R*) product in 95% conversion and 18:1 dr (Figure 6). A similar result (10:1 dr) was obtained in the aforementioned prior work.^22^ Therefore, for this substrate, the presence of the small, remote stereodefined methyl group did not invoke the native “*S*” selectivity of V_*f*_-ATA, with the (*1R*, *3R*) product forming under kinetic conditions. Under thermodynamic conditions, equilibration to the *cis* (*1S*, *3R*) diastereomer was also successfully attained with the same catalyst (62%, 11:1 dr).

Derivatization and separation of the four stereoisomeric products from reactions of racemic substrates allowed us to determine the outcome of the reaction of (*S*)-**11** and both (*R*) and (*S*)-**12** (Figure 6), bearing a 3-CF_3_ substituent. Reactions of **12** showed that both enantiomers of this substrate display diastereodivergent behavior under the two reaction conditions, leading to all four possible stereoisomers of the product from a single biocatalyst when using the correct enantioenriched reagent and reaction condition. The potential to accumulate the (*R*)-amine as the thermodynamic product from the (*S*)-3-CF_3_ again suggests that the ketone behaves as a pseudoachiral reactant. Similarly, the (*S*)-3-methyl ketone yielded the *cis (1R,3S)* product (54% conversion, 8:1 dr). However, this substrate was unselective at all time points under the kinetic conditions, suggesting unselective binding leading to protonation from either face, and a mixture of *cis* and *trans* products that, under thermodynamic conditions, undergo a diastereomer-selective equilibration to the *cis* product.

Not unexpectedly, desymmetrizing the ketone with a larger 3-Ph group resulted in a stronger, catalyst preference to form the (*S*) amine (Figure 6). Under kinetic conditions, both (*S*) and (*R*) 3-phenyl (**S–13** and **R–13**) ketones reacted in nearly full conversion to the *cis* ( *1S* , *3R*) and *trans* ( *1S* , *3S*) amine, respectively. Ketone (***R)*** **–13** remained *cis*-selective under the thermodynamic conditions, while (***S)*** **–13**, whose *cis* (*1R, 3S*) product is a mismatch for the *S*-preference of the transaminase, slowly equilibrated to favor the *1R*-*cis* product but only reached 1.2:1 *dr* after 72 h. While not a particularly selective final outcome, it strongly contrasts the 31:1 diastereomer selectivity for the *trans* product attained under the kinetic conditions and shows that even with a large R group, there is potential for the transaminase to equilibrate to thermodynamic products that oppose its natural *S* enantiopreference.

### 2.4 Enantioselective Catalysis via Dynamic Kinetic Resolution

Finally, we examined 2-substituted cyclohexanones. Like 3-substituted cyclohexanones, these substrates are chiral, and there are four possible stereoisomers from the reaction of a racemic 2-substituted cyclohexanone. However, the presence of the substituent at the α position to the carbonyl enables the potential for dynamic kinetic resolution, either by spontaneous racemization in solution or racemization via the imine or enamine intermediates formed upon condensation of the ketone with PMP (Figure 2A).^38–40^ Therefore, it is possible for a racemic ketone to be fully converted to a single stereoisomer of the amine product. This would require multiple modes of selectivity to be operative, as the two enantiomers of each diastereomer are equal in energy (Figure 7A). A singular product could arise if the enzyme reacted with high enantiomer selectivity for one of the two interconverting enantiomers of the substrate and then, with high diastereomer selectivity, introduced the amine group *cis* or *trans* to the existing methyl group.

**Figure 7:**
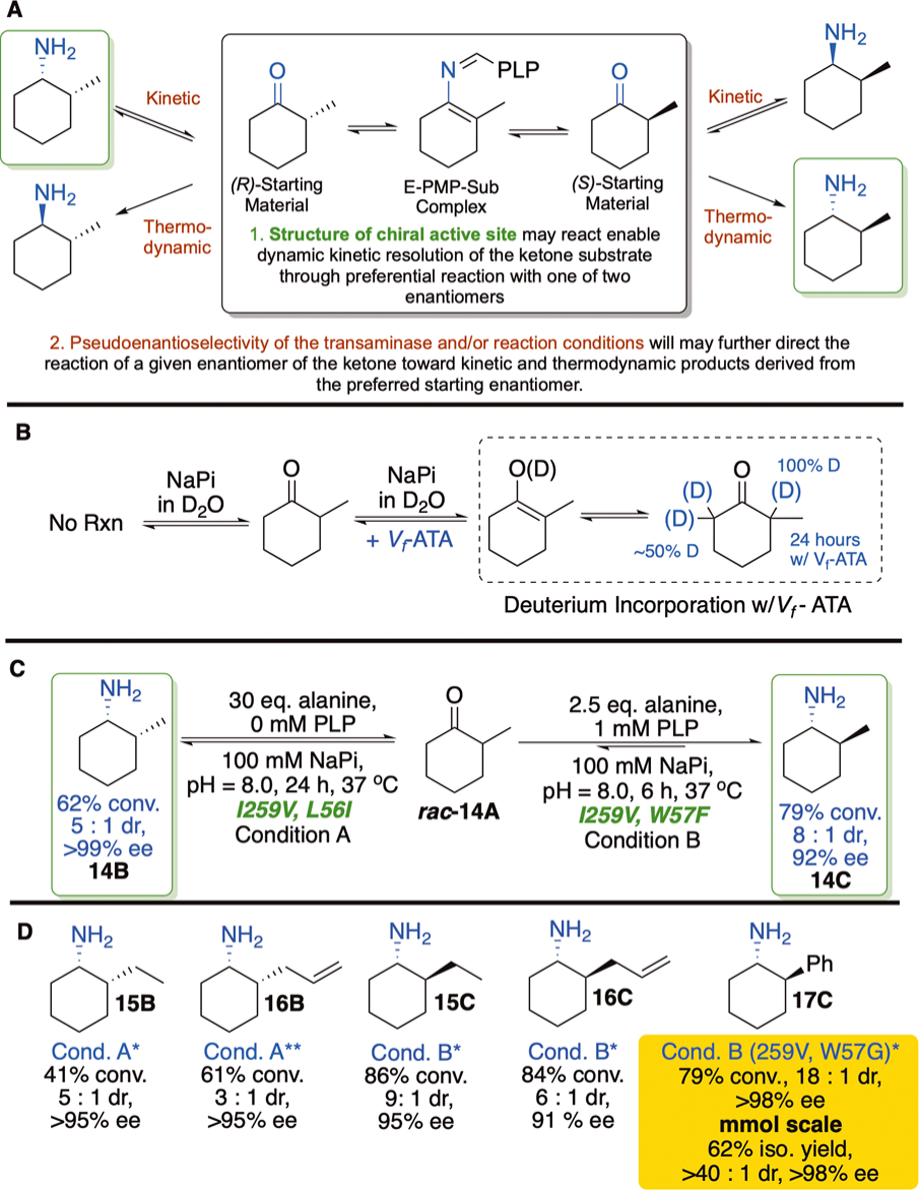
Proposed dynamic kinetic resolution (DKR) enabling divergent, enantioselective synthesis of 2-substituted cyclohexylamines. (A) Tautomerization process enabling DKR and leading to stereoselective reaction outcomes dependent on catalyst and/or thermodynamic control. (B) Outcomes of H/D exchange experiment providing evidence for tautomerization in the presence of cells. See Supporting Information for additional data. (C,D) Outcomes of DKR reactions of 2-substituted cyclohexanones. The *dr* is the ratio of diastereomers based on the major isomer shown in the structure. The *ee* is calculated for the major diastereomer shown in the structure. *The cells were expressed with PYP. **The cells were expressed without PYP.

Previous research examined the outcomes of reactions of 2-methylcyclohexanone with two (*S*)-selective transaminases and one (*R*)*-*selective transaminase in reactions containing 10 eq. of alanine and endogenous PLP. In reaction times sufficient to achieve at least 40% conversion, they found that *Cv*-ATA formed the *cis* (*1S, 3R*) product in 7:1 dr, *Pp*-ATA formed the *trans* (*1S, 3S*) product in <2:1 *dr*, and *Ar*(*R*)­Mut11 formed the opposite *trans* (*1R, 3R*) product in <2:1 *dr.* ^40^

Before studying catalytic reactions, we used an H/D exchange experiment to assess the conditions under which the methyl group of 2-methylcyclohexanone would epimerize (Figure 7B). When the substrate was incubated in D_2_O, NaPi buffer prepared in D_2_O, or PLP alone in D_2_O, we did not observe incorporation of deuterium alpha to the carbonyl. However, when the substrate was incubated in the presence of the cells expressing the ATA, deuteration of all three alpha hydrogens was observed by GC-MS and ^1^H NMR (Figure 7B). Because deuterium presumably incorporates through deprotonation of the alpha proton, which would epimerize the stereocenter, these data suggested the potential for dynamic kinetic resolution of these substrates.

To observe if this proposed dynamic kinetic resolution occurred in practice, 2-methyl (**14A)**, 2-ethyl (**15A**), 2-allyl (**16A**), and 2-phenyl (**17A**) cyclohexanone were subjected to reactions under the aforementioned kinetic and thermodynamic conditions, catalyzed by cells expressing WT and mutated *V*_*f*_-ATAs (Figure 7C,D). In the case of all mutants tested, the major products that formed had 1*S* stereochemistry, suggesting that the increased proximity of the point of asymmetry in 2- versus 3-substituent substrates was sufficient to increase the pseudoenantioselective behavior of the enzyme to furnish (*S*) amines. Nonetheless, the reactions, regardless of mutation, still underwent dynamic equilibration of *cis* amines to *trans* amines over the course of the reaction under thermodynamic, and to a lesser extent, kinetic, conditions, if extended reaction times were used. While the enzyme appeared to exhibit a kinetic preference for the reaction of the (*R*)-ketone for ketones **14–16**, initially accumulating the less stable *cis* (*1S*, *2R*) product, over time, that *cis* product was reconsumed in a diastereomer-selective fashion, such that the product that accumulated over time was the *trans* (*1S*, *2S*) amine, characterized by epimerized stereochemistry at the methyl group. The *dr* for the *trans* products ranged from 6:1 to 40:1, higher than that attained in previously reported reactions containing higher levels of alanine (10 equiv).^40^ Differing mutants were found to either more effectively trap the *cis* product (I259V + L56I) or to equilibrate the reaction more effectively to the *trans* product (I259V + W57L or G). Ketone **17**, bearing a bulky phenyl substituent, required the W57G mutation for high reactivity. Though no mutant was identified to form the *cis* product with high selectivity, the *trans* product was remarkably formed with 79% conversion, 18:1 dr, and >98% ee on an analytical scale and with a comparable outcome on a 0.25 mmol scale.

Two pieces of data more specifically indicated an operative DKR. First, the leftover ketone was observed to be racemic. Second, >50% conversion to a single stereoisomer was observed in several reactions. For example, in the case of 2-methylcyclohexanone, thermodynamic conditions converted 72% of the racemic ketone specifically to the *trans* (S,S) isomer of the product. In the cases of 2-ethyl, 2-allyl, and 2-phenyl substrates, 74–78% conversions to single enantiomers of the *trans* products were observed.

### 2.5 Elucidation of Reaction Dynamics Leading to Diastereodivergence

The transamination reaction in whole cells involves a complex equilibrium including not only the ketone reactant, the amine donor, the amine product, and the ketone byproduct, but also the PLP (aldehyde) cofactor and its PMP (amine) counterpart, which facilitate the ping-pong mechanism of the transaminase (Figure 2). In addition, whole-cell catalysis enables further metabolism of reaction components, in particular the pyruvate derived from the amine donor being metabolized to CO_2_. Having uncovered the described approach to diastereodivergent synthesis, we sought further insight into the origin of selectivity.

First, we investigated the relative rates of the *cis* and *trans* amines reacting in the reverse reaction. According to our initial hypothesis, the *trans* product accumulates because it reacts more slowly in the reverse reaction. To verify this, we incubated the *cis* (**7B**) and *trans* (**7C**) 4-^*t*^Bu amines, whose lower overall reactivity facilitated the measurement, in the presence of 7.5 eq. of pyruvate and found that **7B** fully reacts back to the ketone in less than 5 min, whereas the reaction of **7C** is much slower (Figure 8).

**Figure 8:**
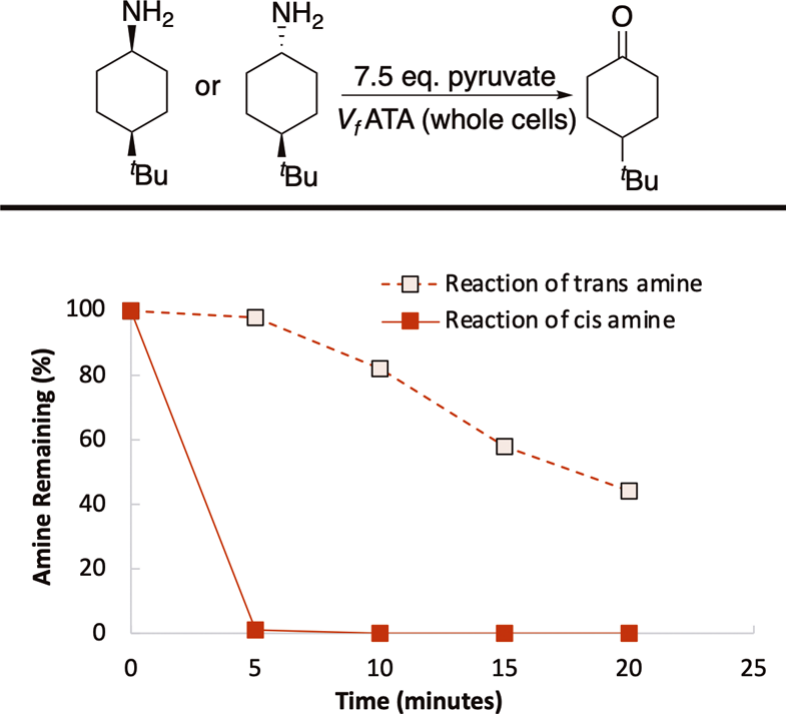
Plot showing the rate of amine consumption when reacted with pyruvate in the presence of cells expressing *V*_*f*_-ATA.

Another significant finding was the specific importance of the PLP cofactor and alanine concentrations to the stereodivergent outcomes of these reactions despite the use of a single biocatalyst. While our original studies quantified the relative concentrations of the ketone reactant and amine products over time, they did not examine the PLP (aldehyde) cofactor and its PMP (amine) counterpart, the alanine reactant, the pyruvate byproduct, and the various possible metabolic derivatives of pyruvate. To comonitor all these species over time, we examined the reaction of **9A** in the presence of ^13^C-alanine (2.5 or 30 equiv) and PLP (0 or 0.1 equiv) by ^19^F and ^13^C NMR and UV–vis spectroscopy (Figure 9A–F).

**Figure 9:**
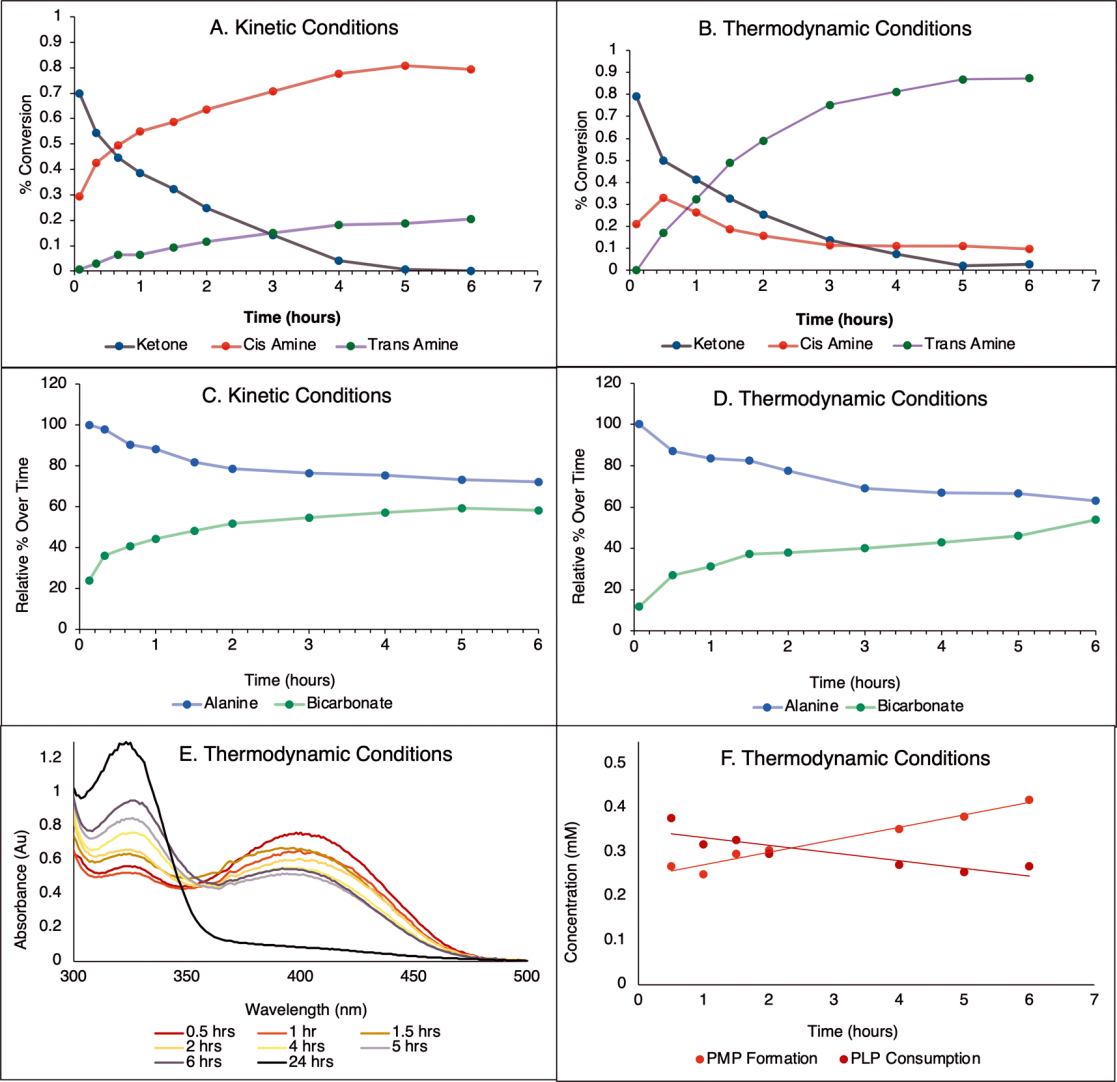
Analysis of the concentrations of reacting species over time in the reaction of 4-trifluoromethyl cyclohexanone (5 mM). (A) Ketone consumption and amine formation under kinetic conditions, as determined by ^19^F NMR. (B) Ketone consumption and amine formation under thermodynamic conditions, as determined by ^19^F NMR. (C) Alanine consumption and pyruvate formation under kinetic conditions, as determined by ^13^C NMR. (D) Alanine consumption and pyruvate formation under thermodynamic conditions, as determined by ^13^C NMR. (E) Analysis of the reaction under thermodynamic condition by UV–vis spectroscopy. (F) PLP consumption and PMP formation under thermodynamic conditions, as determined by UV–vis (shown in panel E). Structures of PLP and PMP are in Figure 2.

Analysis of the reaction over time by ^13^C NMR revealed no observable accumulation of pyruvate; the only species observed by ^13^C NMR were alanine and bicarbonate, which decayed and formed at proportional rates, respectively (Figure 9C,D). This suggests that pyruvate is rapidly metabolized in the reaction, rather than accumulating as a byproduct to facilitate the hypothesized reverse reaction of the kinetic amine products. Interestingly, in studies of the reverse reaction by ^13^C NMR (using ^13^C pyruvate), we found that pyruvate was rapidly metabolized to a range of metabolites, including lactate, bicarbonate, and alanine, suggesting a different metabolic fate for pyruvate when it is slowly generated in the forward reaction versus added in a large quantity to initiate a reverse reaction, such as in Figure 9A.

Monitoring the reaction by UV–vis revealed that over the course of the reaction, the resting state of the PLP cofactor switches from PLP to PMP, owing to reaction with alanine in the first half of the ping-pong mechanism. PLP and PMP could only be detected in the thermodynamic reactions in which supplemental PLP was added (Figure 9E,F). However, given that the kinetic condition has an even larger excess of alanine and less (only endogenous) PLP, it is reasonable to think that this is true to an even greater extent under the kinetic conditions.

Taken together, these data suggest that the isomerization of the kinetic to the thermodynamic product may not involve complete reversal of the kinetic amine to the ketone, but rather the re-reaction of the amine product with the PLP cofactor to form the quinonoid intermediate. When the quinonoid re-releases the amine product, it can release it with epimerized stereochemistry, either at the amine group (in the case of 4- and 3- substituted ketones) or at the substituent (in the case of 2-substituted ketones) (Figure 10). Such a mechanism would not require pyruvate (which was not observed to accumulate in ^13^C NMR studies) and would also be unfavored under the kinetic conditions, in which excess alanine would push the limited PLP cofactor to the PMP resting state, depleting the reaction of the PLP needed for the amine to isomerize.

**Figure 10:**
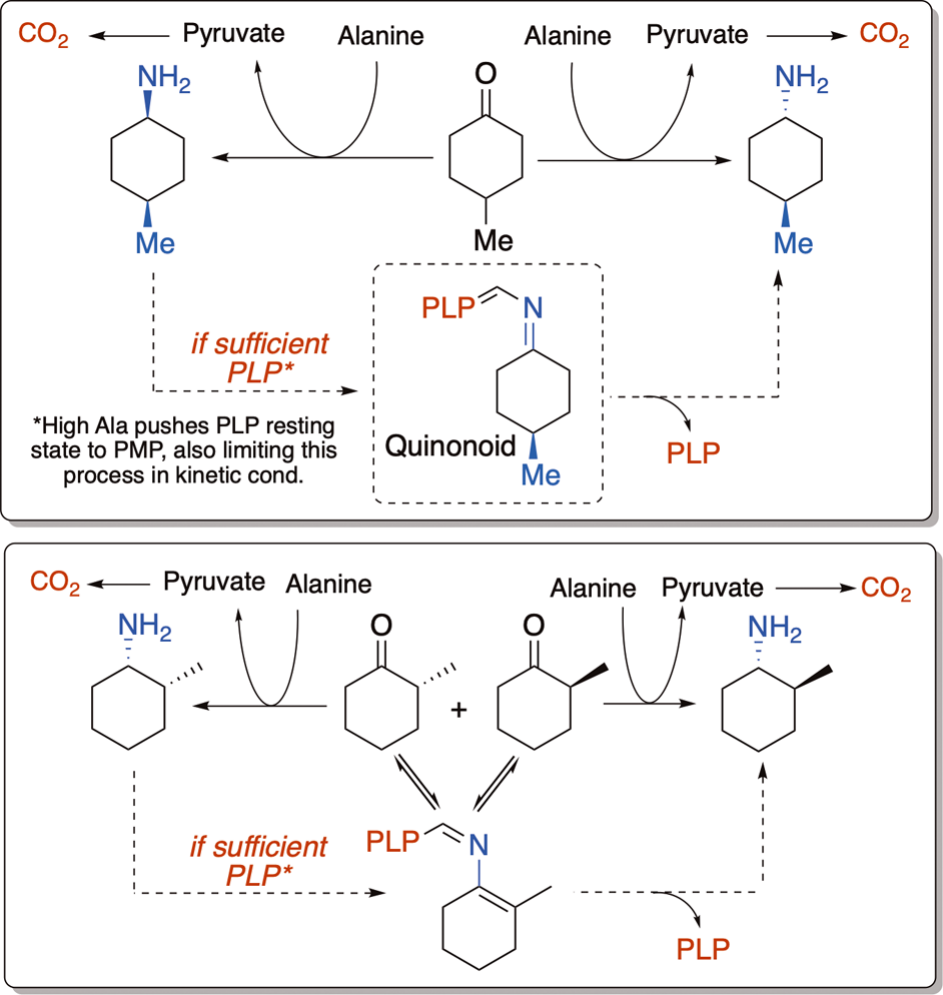
Summary of pathways leading to divergent formation of kinetic and thermodynamic products.

Another finding of our studies was that the supplementation of the expression media with PYP led to cells that more effectively equilibrated the reactions toward the thermodynamic products. To investigate this effect, we purified the transaminases produced with and without the addition of PYP to the cell culture. For WT and mutant *V*_*f*_-ATAs, we obtained 3.5–5 times more holo (active) enzyme per cell weight when expressing the ATA with supplemental PYP (Figure 11A). This was due to both higher yields of the transaminase enzyme and a higher percent holo of the transaminase that was produced. Of note, for cases when the cells containing mutated transaminases outperformed the WT cells, the amount of ATA per cell was actually found to be lower when expressing mutants than when expressing WT-ATA, showing that the reason for the enhanced activity of the mutants was not simply due to more catalyst in the reaction. In fact, the mutants contained less catalyst per reaction than the WT.

**Figure 11:**
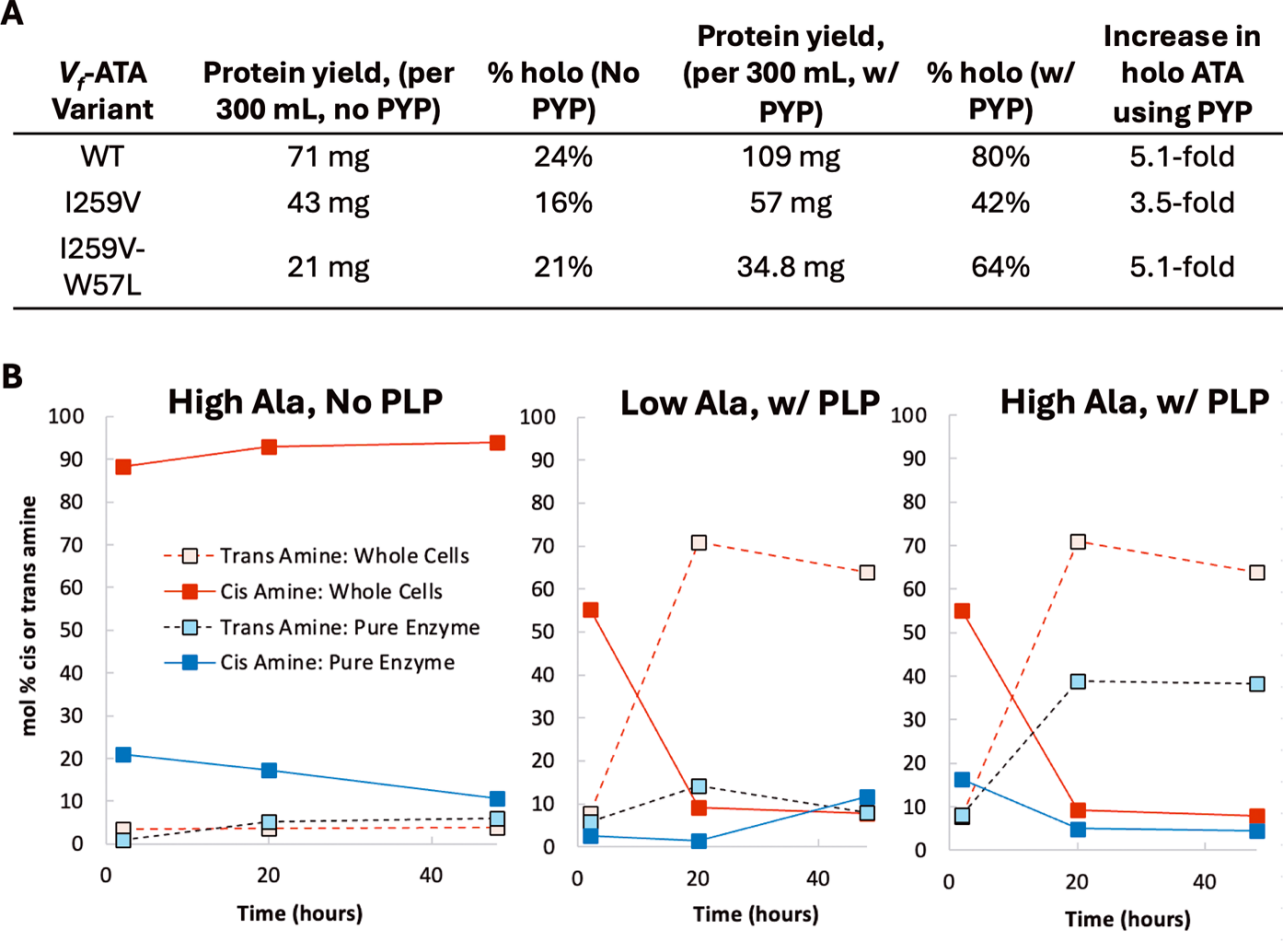
(A) Quantification of ATA yield and % of holoenzyme, determined by Abs 280 nm (protein) and Abs 415 nm (PLP). WT and mutated ATAs were expressed with and without 1.7 mM PYP in the culture media. 600 mL cell cultures were expressed in parallel with and without PYP under otherwise identical conditions. Each cell culture was split in half, with half of each batch purified and the other half left as whole cells in order to collect the comparative reactivity data using enzymes derived from the same original cell culture. (B) Outcomes of reactions catalyzed by whole cells or purified enzymes in the reaction of **1A**. Methods as in Figure 2.

Finally, we compared the outcomes of reactions using the kinetic and thermodynamic conditions when purified enzyme, rather than whole cells, was used as the catalyst. Incidentally, the quantification of the enzyme present in the whole cells also allowed us to estimate the catalyst loading of the whole cells’ reaction to be approximately 0.05 mol % for the WT enzyme, and therefore this catalyst loading was also used in the reactions of the purified enzyme for a consistent comparison.

We found that, to a degree, similar dynamic phenomena were observed using purified *V*_*f*_-ATA; however, the levels of conversion were negligible compared to those attained using the whole-cell catalysts, and the level of selectivity was also not as high, though still divergent within the two conditions (Figure 11B). These results are not surprising given existing literature on effective reaction conditions for pure transaminases. Without supplemental PLP, as in the kinetic conditions, there is little active catalyst to catalyze product formation, and accumulating pyruvate could promote the reverse reaction, leading to the degradation of *cis* product before it can accumulate at a high level. Conversely, under the thermodynamic condition, the absence of either a high excess of amine donor or a secondary enzyme to consume pyruvate means that the equilibrium position is not shifted to the products side, explaining why conversion reaches only 10%.

To supplement these findings, we tested the purified enzyme under a third set of conditions, in which high alanine and high PLP were both used. When using purified enzymes under these conditions, comparable selectivity for the *trans* product was attained as when using whole cells, but the conversion, while higher than under the earlier conditions, was nonetheless ∼50% of that attained with the whole-cell reactions under the same conditions. No set of conditions using the pure enzyme resulted in high selectivity for the kinetic product using the methyl substrate.

Reactions of the slower-reacting **8A** with the purified enzyme reflected similar, but reversed, limitations to that of **1A**. Only trace product was observed under the thermodynamic and kinetic conditions described here. Under conditions with high Ala and added PLP, the reaction occurred with modest conversion (25%) with high selectivity for the kinetic product. No condition with the pure enzyme afforded high conversion or high selectivity for the *trans* product. Therefore, while these studies suggest that the transaminase alone can affect the general reaction dynamics discussed here, these results also show that, at least as this study was designed, the whole-cell environment is essential in achieving the goal of a single catalyst that can lead to the formation of either the kinetic or thermodynamic product in high selectivity and conversion, because it allows the reaction to be conducted successfully with either high Ala/no PLP conditions or low Ala/high PLP conditions.

## 3 Conclusion

Transaminase-catalyzed reactions provide a synthetic method to produce biologically and medicinally valuable, stereodefined amines. Owing to their reversible reaction mechanism, we considered their suitability for stereodivergent reactivity under varied reaction conditions. We found that a high excess of alanine (amine donor) combined with the omission of endogenous PLP cofactor created pseudo-irreversible reaction conditions that afford the kinetic stereoisomers of reactions of 2-, 3-, and 4-substituted cyclohexanones in high stereoselectivity. In contrast, limiting the equivalents of alanine and supplementing the reactions with PLP affords reversible reaction conditions that enable equilibration to the thermodynamic products. In the case of 2-substituted substrates, concurrent DKR enabled the production of enantio- and diastereo-enriched amines from racemic substrates when four stereoisomers were possible. Moreover, this study prompted our investigation of PYP as a key additive to the media when expressing transaminases in order to increase the yield of recombinant expression and the percent of enzymes binding PLP. Together, these results are poised to inform further development of both transaminases as biocatalysts and other biocatalytic methods that, owing to their reversibility, may be directable by kinetic and thermodynamic control.

## Supplementary Material



## Data Availability

The data underlying this study are available in the published article and its Supporting Information.

## Notes

Experimental methods and spectral and chromatographic data (PDF)
