# Supplementary material for: To PLP or Not to PLP: Stereodivergent Transaminase-Catalyzed Reactions Directed by Kinetic and Thermodynamic Control
Source: J Org Chem. 2025 Sep 1;90(36):12655–66. doi: 10.1021/acs.joc.5c01382 (PMC12442079; doi:10.1021/acs.joc.5c01382)
Supplement: Supplementary file 1 [file jo5c01382_si_001.pdf]

Supporting Information

To PLP or Not to PLP: Stereodivergent Transaminase Catalyzed Reactions Directed By Kinetic and Thermodynamic Control

Madeline J. Fitzgerald, Xiaoyan Li, Dawei Peng, Chenlu Qin, Yuehan Sun, Chloe C. Wang, YeePui Yeung, Chi Nguyen, Hanna M. Key*

Table of Contents

I.	Preparation of Whole Cells expressing *Vf*-ATA Enzyme	S2
II.	Whole Cell Reactions of Ketones with *Vf*-ATA Enzyme	S6
III.	Analytical Methods for Catalytic Reactions	S6
IV.	Preliminary Evaluation of Mutants on 96-Well Plates	
V.	H/D Exchange Experiments for 2-Methylcyclohexanone	S11
VI.	Kinetics Studies: Elucidating the Mechanistic Details of the Reversible, Whole-Cell Transaminase Reaction	S13
VII.	Data from ATA Reactions Conducted on Analytical Scale	S20
VIII.	Characterization of Products from Representative Reactions Conducted on Preparative Scale	S72
IX.	References	S82

I. Preparation of Whole Cells expressing *Vf*-ATA

Media Components: Media components were purchased from Research Products International and used without further purification.

Preparation of Agar Plates

To a 2 L Erlenmeyer flask, agar (7.5 g), tryptone (5 g), sodium chloride (5 g), yeast (2.5 g), and water (DD, 500 mL) were added. The resulting solution was autoclaved and then allowed to cool to just above room temperature. Under sterile conditions, Ampicillin, sterilized by syringe filter, (1 mL, 100 mg/mL) was added to the cooled agar solution. The agar was poured into petri dishes (100 mm x 15 mm), forming a thin layer, then allowed to cool, covered, overnight. The final plates were stored, at 4°C in a sealed plastic bag.

Preparation of Stock Solution and Rich LB Media

To a 2 L Erlenmeyer flask, tryptone (10 g), yeast (5 g), sodium chloride (10 g) and water (DD, 500 mL) were added. A cell media buffer was prepared using dibasic sodium phosphate (0.075 mols, 10.75 g), dibasic potassium phosphate (0.032 mols, 5.63 g), monobasic sodium phosphate (0.0025 mols, 0.3 g), monobasic potassium phosphate (0.0022 mols, 0.3 g), ammonium chloride (0.07 mols, 0.75 g) and water (DD, 150 mL). A 20% by weight glucose stock solution was prepared (20 g D-glucose in 100 mL of DD H_2O). Media, buffer, and glucose solutions were autoclaved. Media was stored covered at room temperature for immediate use or portioned into 50 mL falcon tubes and stored at -20 °C for future use. Buffer and glucose solutions were stored covered at room temperature. Stock solutions of ampicillin (100 mg/mL in DD H_2O), and pyridoxine hydrochloride (0.2 M in DD H_2O) were also prepared, stored at -20 °C and sterilized by syringe filter prior to addition to the media. A solution of magnesium sulfate (1 M in DD H_2O), stored at room temperature, was also prepared, and sterilized by syringe filter prior to addition to the media. Rich LB medium was prepared and aliquoted for future use by combining the LB media (500 mL), buffer (50 mL), glucose (20 mL), ampicillin (1 mL, 100 mg/mL) and magnesium sulfate (1 mL, 1 M). SOC media was prepared from LB media (19 mL), potassium chloride (200 uL, 250 mM), magnesium chloride (100 uL, 2 M), glucose (400 uL, 1 M), and water (300 uL, DD).

Plasmid Source

The gene encoding WT Vf-ATA was purchased from GeneScript pre-cloned into a PET 28a vector, featuring a poly-histidine purification tag. The DNA and amino acid sequences corresponding to the gene are given below:

Gene Sequence:
CATATGAACAAACCGCAGAGCTGGGAGGCGCGTGCGGAAACCTACAGCCTGTATGG
CTTTACCGACATGCCGAGCCTGCACCAACGTGGTACCGTGGTTGTGACCCACGGCGA
GGGCCCGTACATTGTTGACGTGAACGGCCGTCGTTATCTGGATGCGAACAGCGGTCT
GTGGAACATGGTTGCGGGTTTTGACCACAAGGGCCTGATCGATGCGGCGAAAGCGC
AGTACGAACGTTTCCCGGGCTATCACGCGTTCTTTGGTCGTATGAGCGATCAAACCG
TGATGCTGAGCGAGAAGCTGGTTGAAGTGAGCCCGTTCGACAGCGGCCGTGTGTTCT
ACACCAACAGCGGTAGCGAGGCGAACGATACCATGGTGAAGATGCTGTGGTTCCTG

CACGCGGCGGAAGGTAAACCGCAGAAGCGTAAAATCCTGACCCGTTGGAACGCGTA
CCATGGTGTTACCGCTGTGAGCGCGAGCATGACCGGTAAACCGTATAACAGCGTTTT
CGGTCTGCCGCTGCCGGGCTTTGTGCACCTGACCTGCCCGCACTACTGGCGTTATGGC
GAGGAAGGCGAGACCGAGGAACAGTTTGTTGCGCGTCTGGCGCGTGAGCTGGAGGA
AACCATTCAACGTGAAGGCGCGGACACCATCGCGGGTTTCTTTGCGGAACCGGTTAT
GGGTGCGGGTGGCGTGATTCCGCCGGCGAAGGGTTACTTCCAAGCGATCCTGCCGAT
TCTGCGTAAATATGACATCCCGGTTATTAGCGATGAGGTGATCTGCGGTTTTGGCCGT
ACCGGTAACACCTGGGGCTGCGTTACCTACGACTTCACCCCGGATGCGATCATTAGC
AGCAAGAACCTGACCGCGGGTTTCTTTCCGATGGGCGCGGTGATTCTGGGTCCGGAA
CTGAGCAAACGTCTGGAGACCGCGATCGAAGCGATTGAGGAATTTCCGCATGGTTTT
ACCGCGAGCGGTCATCCGGTTGGTTGCGCGATTGCGCTGAAGGCGATTGATGTTGTG
ATGAACGAGGGTCTGGCGGAAAACGTGCGTCGTCTGGCGCCGCGTTTCGAGGAACGT
CTGAAACACATCGCGGAGCGTCCGAACATTGGTGAATATCGTGGTATCGGCTTTATG
TGGGCGCTGGAGGCGGTTAAGGACAAAGCGAGCAAGACCCCGTTCGATGGTAACCT
GAGCGTGAGCGAACGTATTGCGAACACCTGCACCGACCTGGGTCTGATCTGCCGTCC
GCTGGGTCAGAGCGTTGTGCTGTGCCCGCCGTTCATCCTGACCGAGGCGCAAATGGA
CGAAATGTTTGATAAGCTGGAGAAAGCGCTGGATAAAGTTTTCGCGGAAGTGGCGTA
ACTCGAG

Amino Acid Sequence:
HMNKPQSWEARAETYSLYGFTDMPSLHQRGTVVVTHGEGPYIVDVNGRRYLDANSGL
WNMVAGFDHKGLIDAAKAQYERFPGYHAFFGRMSDQTVMLSEKLVEVSPFDSGRVFYT
NSGSEANDTMVKMLWFLHAAEGKPQKRKILTRWNAYHGVTAVSASMTGKPYNSVFGL
PLPGFVHLTCPHYWRYGEEGETEEQFVARLARELEETIQREGADTIAGFFAEPVMGAGG
VIPPAKGYFQAILPILRKYDIPVISDEVICGFGRTGNTWGCVTYDFTPDAIISSKNLTAGFFP
MGAVILGPELSKRLETAIEAIEEFPHGFTASGHPVGCAIALKAIDVVMNEGLAENVRRLAP
RFEERLKHIAERPNIGEYRGIGFMWALEAVKDKASKTPFDGNLSVSERIANTCTDLGLICR
PLGQSVVLCPPFILTEAQMDEMFDKLEKALDKVFAEVA-

Transformation of BL21-DE3 *E. coli* cells and Expression of *Vf*-ATA, 600 mL Scale

Fisher brand BL21-DE3 competent *E. coli* cells (**Catalog number** EC0114) were thawed on ice. To a 1 mL culture tube, cells (50 uL) and the desired *Vf*-ATA plasmid (1.5 uL) were added. This process was repeated for the corresponding DNA of each variant of interest. For a single variant of interest: the tube was placed back on ice for 30 mins, then heat shocked at 42 °C for 30 seconds and put back on ice for 2 minutes. Sterile SOC media (500 uL) was added to the tube. Following SOC addition, each tube incubated for one hour at 250 rpm and 37 °C. Under sterile conditions, an aliquot (200 uL) from each culture was spread on an LB agar plate and incubated overnight (~17 hours) at 37 °C. The next morning, the plate was removed from the incubator and placed in the fridge until colonies were selected, generally within 1 day. A single colony was picked from the plate and transferred to a culture tube containing rich LB media (3 mL, recipe described previously) and incubated at 37 °C and 250 rpm for 17 hours.

Following the incubation period, to a 2 L Erlenmeyer flask containing 500 mL of LB media, 3 mL of overnight culture, cell media buffer (50 mL), glucose (20 mL, 20% by weight), magnesium sulfate

solution (1 mL, 1 M), and ampicillin (1 mL 100 mg/mL) were added under sterile conditions. Pyridoxine hydrochloride (5 mL, 0.2 M) was added to the media when indicated. The 2 L flask was covered loosely with aluminum foil and incubated at 37 °C and 250 rpm. After 7 hours, IPTG solution (500 uL, 1M in DD H_2O) was added and incubation continued overnight (15 hours) at 37 °C and 250 rpm. Following incubation, the cells were centrifuged (10 min, 4000 rpm) and resuspended in sodium-phosphate pH 8.0 (100 mM) buffer to a concentration of 80 g cells/L buffer. The cells were transferred to 15 mL tubes and stored at -80°C until use in a catalytic reaction. Cells were generally stored in 8 mL aliquots and could be used with a few freeze-thaw cycles without apparent impact on the reaction. The impact of repeated freeze-thaw cycles was not investigated in detail.

Site Directed Mutagenesis

Site directed mutagenesis (SDM) was performed using an Agilent QuikChange Lightning Standard or Multi Site-Directed Mutagenesis Kit. The desired primers were purchased as RxnReady Primer Pools from Integrated DNA Technologies and diluted per the kit instructions. The kit supplied dNTP mix was diluted with 40 uL of water (DD), centrifuged at 2,000 rpm for 1 minute and aliquots (5 uL) were transferred to PCR tubes for storage at -20°C.

For a given SDM reaction either a primer for a single point mutation (ex. W57G) or degenerate primer mixture encoding multiple mutations at the same site was used. To a PCR tube containing aliquoted dNTP mix (5 uL), 10x QuikChange Lightning Multi reaction buffer (2.5 uL), water (dd, 12.5 uL), ds-DNA (1.5 uL), diluted primer solution (1.5 uL), and QuikChange Lightning Multi enzyme blend (1 uL) were added, in that order. The reaction was then cycled using the parameters outlined below:

	**1 Temperature**	**3 Temperatures, 30 Cycles**			**1 Temperature**
**Temperature (°C)**	95.0	95.0	55.0	65.0	65.0
**Time (mins)**	2.0	0.33	0.5	5.0	5.0

Following cycling, the reaction was placed on ice for two minutes, followed by addition of Dpn digestive enzyme (1 uL) which was mixed by pipette. The reaction mixture was set in a 37 °C water bath for at least 60 minutes to digest the parent DNA.

Transformation SDM Product into XL10-Gold Ultracompetent Cells

XL10-gold ultracompetent cells (45 uL) were gently thawed on ice and transferred to a pre-chilled 14-mL round-bottom culture tube. To the culture tube, the supplied beta-mercaptoethanol (2 uL) was added, and the contents was incubated on ice for 10 minutes with swirling every two minutes. To the culture tube, DPN treated products from SDM (1.5 uL) were added and the tube was swirled, followed by 30 minutes of incubation on ice. The tube was then heat shocked in a 42 °C water bath for exactly 30 seconds and returned to ice for two minutes. To the culture tube, pre-heated SOC media (0.5 mL) was added, followed by incubation at 37°C and 250 rpm for one hour.

Following incubation, a pool of SOC media (100 uL) was pipeted on an agar plate and the transformation reaction (50 uL) was diluted on the plate into the media pool and the plate was spread, covered, and incubated overnight at 37 °C for 16 hours. The next morning, the plate was removed from the incubator and placed in the fridge. Within 24 hours, single colonies were picked from the plate and transferred to individual culture tubes. For plates corresponding to SDM using degenerate/mixed primers, a culture loop was swiped across the plate to enable transfer of material from multiple colonies. To the

culture tube, pre-prepared LB media (3 mL) was added, and the tube was incubated at 37 °C and 250 rpm for 17 hours.

DNA Isolation

A QIAprep Spin Miniprep Kit was used to isolate the DNA from SDM. The overnight culture was centrifuged in the original culture tubes for five minutes at 3,500 rpm and 20 °C, to pellet the cells. The DNA was then isolated according to the instructions provided by the kit.

The concentration (ng/uL) of extracted DNA was measured using a nano-drop using the built in setting for dsDNA quantification. DNA plasmids corresponding to variants of interest sequenced at the UC Berkeley DNA Sequencing Facility. Mutations targeting residues 1 – 258 were analyzed using a T7 forward primer and mutations targeting residues 259+ were analyzed using a T7 reverse primer. An example of raw sequencing data is given below:

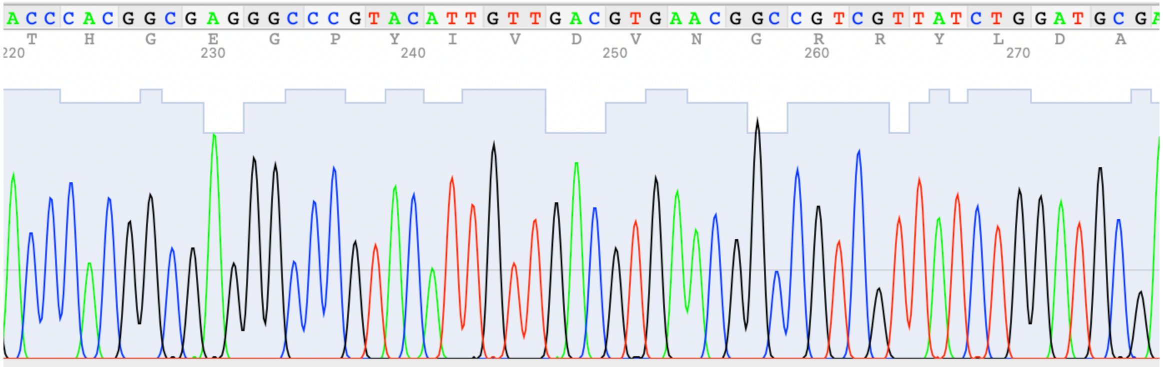

Translated sequences were compared to that of the *Vf*-ATA wildtype. The finding from a sample mutant is given below:

MNKPQSWEARAETYSLYGFTDMPSLHQRGTVVVTHGEGPYIVDVNGRRYLDANSG**L**WNMVAG
FDHKGLIDAAKAQYERFPGYHAFFGRMSDQTVMLSEKLVEVSPFDSGRVFYTNSGSEANDTMV
KMLWFLHAAEGKPQKRKILTRWNAYHGITAVSASMTGKPYNSVFGLPLPGFVHLTCPHYWRYGE
EGETEEQFVARLARELEETIQREGADTIAGFFAEPVMGAGGVIPPAKGYFQAILPILRKYDIPVISDE
V**I**CGFGRTGNTWGCVTYDFTPDAIISSKNLTAGFFPMGAVILGPELSKRLETAIEAIEEFPHGFTASG
HPVGCAI

Protein Purification and Determination of %holo

Cell suspensions were thawed in a room-temperature water bath, decanted to 50 mL plastic tubes, and lysed on ice by sonication (120 sec total on, 4 sec on, 8 sec off, 45% power). Cell debris was removed by centrifugation (9,000 rpm, 15 min, 4 °C). Ni-NTA (2.5 mL, 50% suspension per 300 mL cell culture) was rinsed with Ni-NTA lysis buffer in a small plastic column, and the lysate was added to the column and allowed to pass through, followed by additional washing of the resin with Ni-NTA wash buffer (~25 mL). The protein was eluted from the resin with 1 mL fractions of Ni-NTA elution buffer (50 mM NaPi, 250 mM NaCl, 250 mM Imidazole, pH = 8.0), and the fractions containing greater than 1 mg/mL protein were pooled and passed through a NAP column to exchange the buffer to 100 mM NaPi, pH = 8.0. The samples were analyzed by UV-Vis spectroscopy at 280 nm and 415 nm to quantify the [ATA] and [bound PLP]. The %holo was calculated by dividing the [PLP]/[protein].

II. Whole Cell Reactions of Ketones with *Vf*-ATA

Chemical Sourcing: Chemicals were procured from commercial supplies and used without further purification. These chemicals included solvents, alanine, PLP, PYP, ketones, and standards of amine products, when commercially available (4-Me, 2-Me, 4-tBu, 4-Ph). Suppliers included Sigma Aldrich, CombiBlocks, 1ClickChemistry, Fisher, Oakwood, RPI, ChemImpex, and Cambridge Isotopes.

Preparation of Stock Solutions

Stock solutions of alanine (445 mg in 5 mL H_2O, 1M), and pyridoxal phosphate (PLP, 12.4 mg in 5 mL H_2O, 10 mM) were prepared. 1 M substrate solutions of the ketones were prepared in DMSO: 4-methylcyclohexanone (31 uL in 219 uL DMSO), 4-ethylcyclohexanone (35 uL in 215 uL DMSO), 4-isopropylcyclohexanone (39 uL in 211 uL DMSO), 4-methoxycyclohexanone (32 uL in 218 uL DMSO), 4-oxocylcohexanecarbonitrile (29 uL in 221 uL DMSO), 4-trifluoromethylcyclohexanone (34 uL in 216 uL DMSO), 4-isopropoxycyclohexanone (39 uL in 211 uL DMSO), 4-benzyloxycyclohexanone (46 uL in 204 uL DMSO), 4-tert-butyl-oxocyclohexanecarboxylate (49 mg in 250 uL DMSO), 3-methylcyclohexanone (31 uL in 219 uL DMSO), 3-trifluoromethylcyclohexanone (34 uL in 216 uL DMSO), 2-methylcyclohexanone (30 uL in 220 uL DMSO), 2-allylcyclohexanone (37 uL in 213 uL DMSO).

Standard Reaction Set-Up

The following reagents were added to 20 mL glass vials for each reaction. Reagents were scaled accordingly to increase the total volume of the reaction and the number of equivalents indicated for a given reaction. Standard reactions were filled to 2 mL with NaPi buffer (100 mM, pH 8) after accounting for the volume taken up by the stock solutions of reagents. Unless otherwise noted, the following conditions were used. Reactions were incubated at 37 °C and 400 rpm for the amounts of time indicated in later sections.

General Kinetic condition, K: Cells (2 mL cell suspension, 80 mg cells/mL of 100 mM NaPi, pH 8), ketone (20 µL, 1 M in DMSO), alanine (600 µL, 1 M in H_2O, 30 eq.), and buffer (to a total volume 2 mL, 100 mM NaPi, pH 8). Reactions were sealed with plastic, air tight caps except in experiments which monitored the reaction progress with an air permeable membrane. Reactions were incubated at 37C for the time indicated for each substrate with 300 rpm of rotation.

General Thermodynamic condition, T: Cells (2 mL, 80 mg/mL in 100 mM NaPi, pH 8), ketone (20 µL, 1 M in DMSO), alanine (300 µL, 1 M in H_2O, 30 eq.), PLP (100 µL, 10 mM in H_2O, 0.1 eq.), and buffer (to a total volume 2 mL, 100 mM NaPi, pH 8). Reactions were sealed with plastic, air tight caps except in experiments which monitored the reaction progress with an air permeable membrane. Reactions were incubated at 37C for the time indicated for each substrate with 300 rpm of rotation.

III. Analytical Methods for Catalytic Reactions

General Instrumentation: Reactions were analyzed by GC, GC-MS, and NMR. GC analysis was performed on an Agilent 7890B Series GC with an FID detector and a PAL 3 autosampler. Chiral separations were performed using a CP-Chirasil-Dex CB Column. GC data was analyzed within the native ChemStation Software and chromatograms were further processed and visualized using MestreNova. GC-MS analysis was conducted using a Bruker GC-MS. Data was viewed within the native MSWS software

and further visualized using OpenChrom/Eclipse 1.3. NMR data was acquired on a Bruker 400 MHz NMR. Data was processed and visualized using MestreNova. Structural assignments were made with additional information from gCOSY experiments.

Analysis of Conversion: Calibration curves for 4-methyl cyclohexanone, 4-methylcyclohexylamine, 2-methyl cyclohexanone and 2-methylcyclohexylamine indicated that the response factors by GC-FID for a ketone and its corresponding amine were within 5%, which is not unexpected for a reaction that does not introduce carbon atoms into the structure. Given the similarities, conversions for other reactions were calculated without adjustment for the relative response factors of each ketone/amine pair.

Analysis of Selectivity: In most cases, ketones and both amine diastereomers were well separated in achiral GC. In those cases, the achiral GC of the direct amine product was used to calculate the diastereoselectivity. In some cases, when suitable separation was not achieved for all three signals, the amines were derivatized with acetic anhydride and then analyzed as their respective acyl amides, using either a chiral or achiral column. In these cases, chromatograms from the original amines and their respective amides are provided in a later section. Derivatization was also used to separate the four amine products derived from 3- and 4-substituted ketones.

Method to Analyze Reactions by GC/GC-MS

To a 1.5 mL centrifuge tube, a portion of the reaction (200 uL), sodium hydroxide (20 uL, 10 M) and ethyl acetate (800 uL) were added. The vial was vortexed and centrifuged at 10,000 rpm for 2 minutes. The top, organic, layer (750 uL) was transferred to a GC or GC-MS vial for analysis. GC-MS facilitated assignment of the signals in the GC as the amine products, while NMR (see below) enabled assignment of the *cis* and *trans* isomers. Sample GC and GC-MS chromatograms have been included below. Generally, the ketone and amine were the only significant compounds observed in the extracts, other than indole, which is another metabolite produced by cells.

Sample GC traces (complete data for all compounds found in subsequent section):

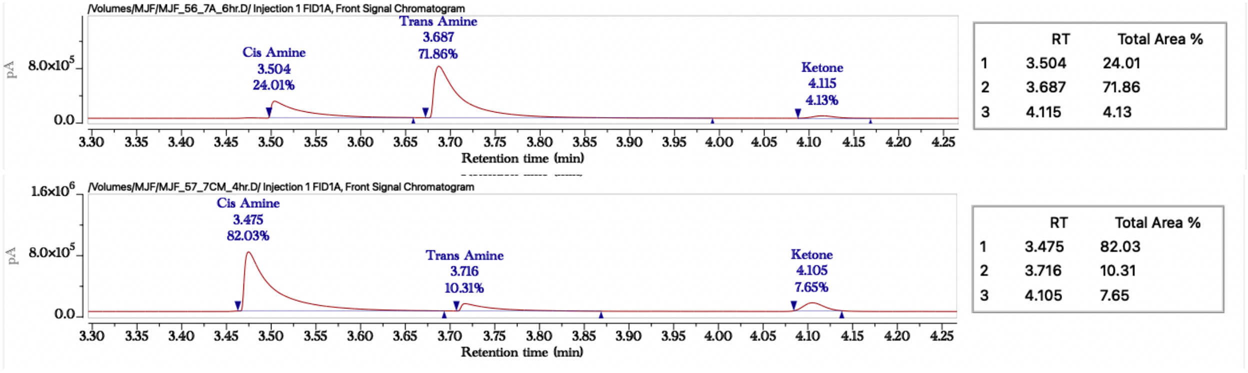

Analysis of Derivatized Reaction Products by GC/GC-MS

To each of two 1.5 mL centrifuge vials, a portion of the incubated reaction (500 uL), sodium hydroxide (40 uL, 10 M) and diethyl ether (600 uL) were added. Use of diethyl ether, rather than ethyl acetate, was found to be most effective for the success of the small scale, crude derivatizations. The contents of each tube were vortexed and then centrifuged at 10,000 rpm for 2 minutes. The top (organic) layers (500

uL) from each tube were combined in a 2 mL glass vial. The organic layer was dried with a small amount of magnesium sulfate and transferred to a new vial. Acetic anhydride (10 uL) was added, the reaction was mixed gently on a vortex (setting 2) for at least 2 hours and up to overnight, after which H_2O (1 mL) was added, and the organic layer was transferred to a GC vial for analysis. A sample chiral GC chromatogram has been included below. Generally, the ketone and amine were the only significant compounds observed in the extracts, other than indole (a cellular metabolite) and its derivative.

Sample chiral GC data for derivatized reaction (complete data for all compounds found in subsequent section):

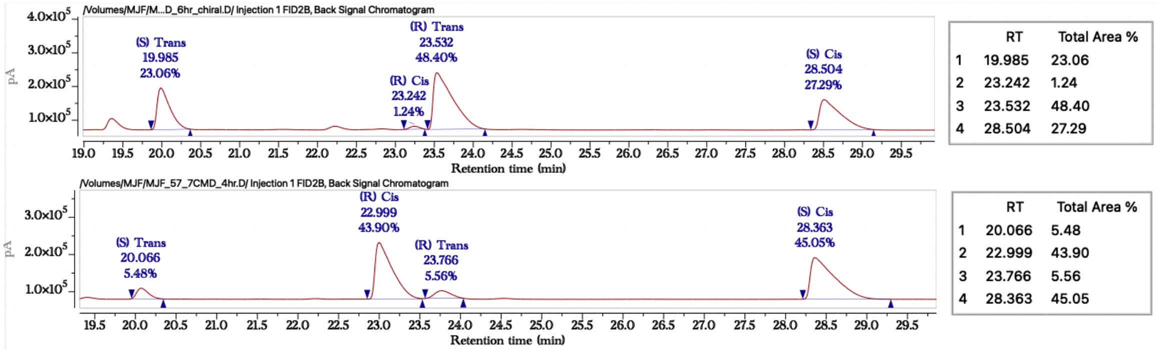

GC-Methods

Analysis methods for the reaction of each substrate are given below. In all cases except 4-isopropylcyclohexanone (4-iPr), some separation of the *cis* and *trans* isomers was achieved by achiral GC. The products from the 4-iPr substrates fully overlapped in all methods attempted, while in select other cases, the two amines partially overlapped, or one amine overlapped with the ketone. To improve the separation in these cases, the amines were derivatized to the corresponding amide using acetic anhydride, and the amides were subsequently analyzed. The formation of the amine and amide products were confirmed using GC-MS.

**Reactions analyzed with this method**	**Method Description**
4-Methylcyclohexanone,<br>3-Methylcyclohexanone,<br>2-Methylcyclohexanone,<br>2-Ethylcyclohexanone<br>4-Trifluoromethylcyclohexanone,<br>3-Trifluoromethylcyclohexanone,<br>4-Ethylcyclohexanone,<br>4-Methoxycylcohexanone,<br>4-Oxocyclohexanecarbonitrile<br>2-Allylcyclohexanone	75 °C (3.5 min)<br>To 108 °C (10 °C per min)<br>To 180 °C (60 °C per min)<br>Hold 180 °C (2 min)
4-Isopropylcyclohexanone	75 °C (3.5 min)<br>To 120 °C (10 °C per min)<br>To 180 °C (30 °C per min)<br>Hold 180 °C (2 min)

3-phenylcyclohexanone<br>4-phenylcyclohexanone	50 °C to 200 °C (6 °C per min)
4-tert-butyl-oxocyclohexanecarboxylate	50 °C<br>To 160 °C (8 °C per min)<br>Hold 160 °C (2 min)<br>To 200 °C (40 °C per min)<br>Hold 200 °C (1 min)
4-Isopropoxycyclohexanone,<br>4-Isopropylcyclohexanone (derivative)	100 °C (2 min)<br>To 180 °C (80 °C per min)<br>Hold 180 °C (3 min)<br>To 200 °C (10 °C per min)<br>Hold 200 °C (1 min)
4-Benzyloxycyclohexanone	100 °C (2 min)<br>To 180 °C (20 °C per min)<br>Hold 180 °C (1 min)<br>To 200 °C (10 °C per min)
3-Phenylcyclohexanone (derivative)	Isothermal, 165 °C (60 min)
2-Methylcyclohexanone (derivative),<br>2-Ethylcyclohexanone (derivative),<br>3-Methylcyclohexanone (derivative),<br>3-Trifluoromethylcyclohexanone (derivative)	Isothermal, 115 °C (33 min)<br>Used on a chiral column.
3-Trifluoromethylcyclohexanone (derivative),<br>4-Trifluoromethylcyclohexanone (derivative)	Isothermal, 115 °C (10 min)
2-Allylcyclohexanone (derivative)	Isothermal, 140 °C (20 min)
2-Methylcyclohexanone, 3-Methylcyclohexanone, 2-Allylcyclohexanone (ketone separation)	Isothermal, 110 °C (20 min)

Analysis of Reaction Products by ^{1}H NMR

To each of two 1.5 mL centrifuge tubes, the incubated reaction (500 uL), sodium hydroxide (40 uL, 10 M), and NMR solvent (600 uL) were added, and the contents of each tube were vortexed and centrifuged at 10,000 rpm for 2 minutes. The organic layers (500 uL) from each tube were combined in a 2 mL glass vial. Magnesium sulfate drying agent was added and the dried extract was transferred to an NMR tube. $CDCl_3$ or C_6D_6 were used as the NMR solvent. Use of C_6D_6 was found to enable more effective extraction and greater separation of signals from residual water and DMSO from those of the products. NMR taken on a 400 mHz instrument and was primarily used to assign *cis*/*trans* stereochemistry. The stereochemistry was assigned on the basis of the chemical shift and splitting of the proton bonding to the amine carbon. *Trans* products were characterized by small coupling constants and higher chemical shifts, whereas *cis* amines were characterized by larger coupling constants and smaller chemical shifts. In the cases of several amine products (4-Me, 2-Me, 4-tBu, 4-Ph) the identity of the products could be further confirmed by comparison to authentic, commercially available amines which revealed these characteristic trends. In other cases, the amines were assigned by analogy to these trends.

A sample NMR spectrum has been included below. Complete data are found in a later section.

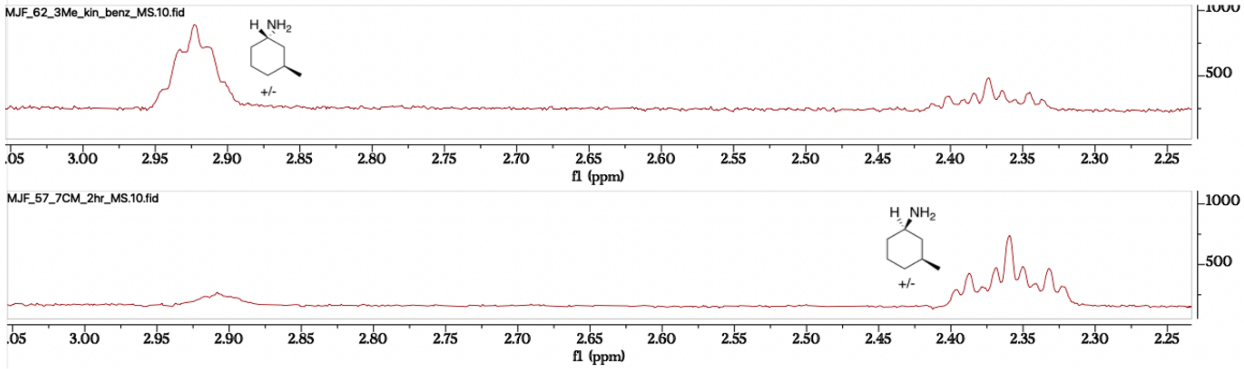

IV. Preliminary Evaluation of Mutants on 96-Well Plates

Expression of Mutant Transaminase Arrays on 96 Well Plates to Enable Efficient Discovery of Improved Mutants for Kinetic and Thermodynamic Conditions

BL21-DE3 *E. coli* cells were defrosted on ice. To a 14 mL culture tube, cells (30 uL) were added. Then, 1.5 uL of a mixture of DNA corresponding to multiple point mutants (prepared by site directed mutagenesis using degenerate primers, described previously) was added to the cells. The tube was placed back on ice for 30 mins, then heat shocked at 42 °C for 30 seconds and put back on ice for 2 minutes. SOC media (500 uL) was added to the tube. Following SOC addition, each tube was placed in the incubator for one hour at 250 rpm and 37 °C. Under sterile conditions, the contents (200 uL) from the tubes were spread on an agar plates and incubated overnight (~17 hours) at 37 °C. The next morning, the plates were removed from the incubator and placed in the fridge for up to one day before being used to inoculate a 96-well plate of 1 mL cultures.

An empty, 96 deep-well culture plate was autoclaved. To each deep-well of the plate, rich LB media (1 mL) was added. Single colonies were picked from the previously prepared agar plates and transferred to corresponding wells. The 96-deep well culture plate was then incubated overnight at 37 °C and 250 rpm for 17 hours.

Following incubation, an expression plate was induced. To a new, sterilized, 96 deep-well plate, rich LB media (1 mL per well), and the corresponding overnight culture cells (10 uL), were added. Pyridoxine hydrochloride (20 uL, 0.1 M) was added to wells corresponding to variants aimed at improving the rate and selectivity of thermodynamic favoring reactions. This expression plate was incubated at 37 °C and 250 rpm for 7 hours, followed by IPTG addition (10 ul, 0.1 M to each well) and 16 hours of further incubation. The expression plate was removed from the incubator and the cells were allowed to settle. Media (400 uL) was removed from each well to increase cell concentration. The remaining cells were gently mixed by pipet to resuspend and were ready for use in reactions.

Reaction Set-Up, 96 Well Plate

The following reagents, according to desired condition, were added to glass vials arrayed on a 96-well plate. For the sake of organization, the wells were set to mimic the expression plate. Reagents were generally added using a multi-chamber pipette. Reactions were incubated at 37 °C and 250 rpm for varying

lengths of time. **Kinetic condition, K:** Cells (200uL from the above expression plate), ketone (5 μL, 0.2 M in DMSO), alanine (30 μL, 1 M in H_2O, 30 eq.), and buffer (70 uL, 100 mM NaPi, pH 8). **Thermodynamic condition, T:** Cells (200 uL from the above expression plate), ketone (5 μL, 0.2 M in DMSO), alanine (5 μL, 1 M in H_2O, 30 eq.), PLP (10 μL, 10 mM in H_2O, 0.1 eq.), and buffer (90 uL, 100 mM NaPi, pH 8).

Analysis of Products by GC on a 96 Well Plate

To the reaction vessel, sodium hydroxide (10 uL, 10 M), and ethyl acetate (300 uL), were added, by multi-chamber pipet. The contents of the wells were mixed by pipet, bringing the bottom 250 uL to the top, and repeating eight times. The organic layer (top 250 uL) for each well was then transferred to new glass tubes in a corresponding analysis plate. To each of the new vials, ethyl acetate (400 uL) was added. The complete plate was then analyzed by GC directly from the 96 well array using a PAL3 autosampler fitted with a refrigerated sample tray, using the method indicated above for each substrate.

V. H/D Exchange Experiments for 2-Methylcyclohexanone (2-Me)

2-Methylcyclohexanone was used as a representative substrate. NaPi pH 8.0 buffer was prepared using deuterium oxide (10 mM, 0.00209 g NaH_2PO_4, 0.01527 g Na_2HPO_4, 5 mL D_2O). An aliquot of the L56I + I259V cells was resuspended in the NaPi D_2O buffer by transferring 2 mL of the original cells to a new 15 mL falcon tube. This tube was then centrifuged at 4,000 rpm and 4 °C for 10 minutes, following by the addition of NaPi D_2O (2 mL) to the tube and shaking to mix. Separate 2 mL reactions were prepared in 20 mL glass vials containing varying combinations of NaPi D_2O buffer, 2-Me ketone (1M in DMSO) NaPi D_2O cells and alanine (1 M in D_2O). Reactions were then extracted and measured by GCMS and ^1H NMR. The initial reaction time points used were 24 and 48 hours.

A single extraction protocol was implemented, creating one sample per reaction to be analyzed by both GC-MS and NMR. To a 1.5 mL centrifuge tube NaOH (40 uL 10 M), reaction mixture (500 uL), and deuterated benzene (C_6D_6, 600 uL) were added (repeated for a total of two tubes for each reaction). The contents were vortexed on setting 10 for ~10 seconds and centrifuged for 2 minutes at 10,000 rpm. The top layers, ~500 uL, from each centrifuge tube, were combined in a 2 mL glass vial. Magnesium sulfate drying agent was added to the vial, and the solution was transferred to a GC-MS capped vial. Following GC-MS analysis, the contents of the GC-MS vial were transferred directly to an NMR tube for the ^1H analysis. The commercial 2-Me ketone was analyzed in C_6D_6 via both the 18-minute GC-MS method and with the ^1H NMR, to confirm the signals associated with the pure substrate. A COSY ^1H NMR was also obtained for the commercial 2-Me ketone to enable further assignment of hydrogens and their corresponding splitting patterns. NMR samples contained 1 uL of pure ketone in 650 uL C_6D_6.

COSY of 2-methylcyclohexanone, used to interpret H/D exchange results

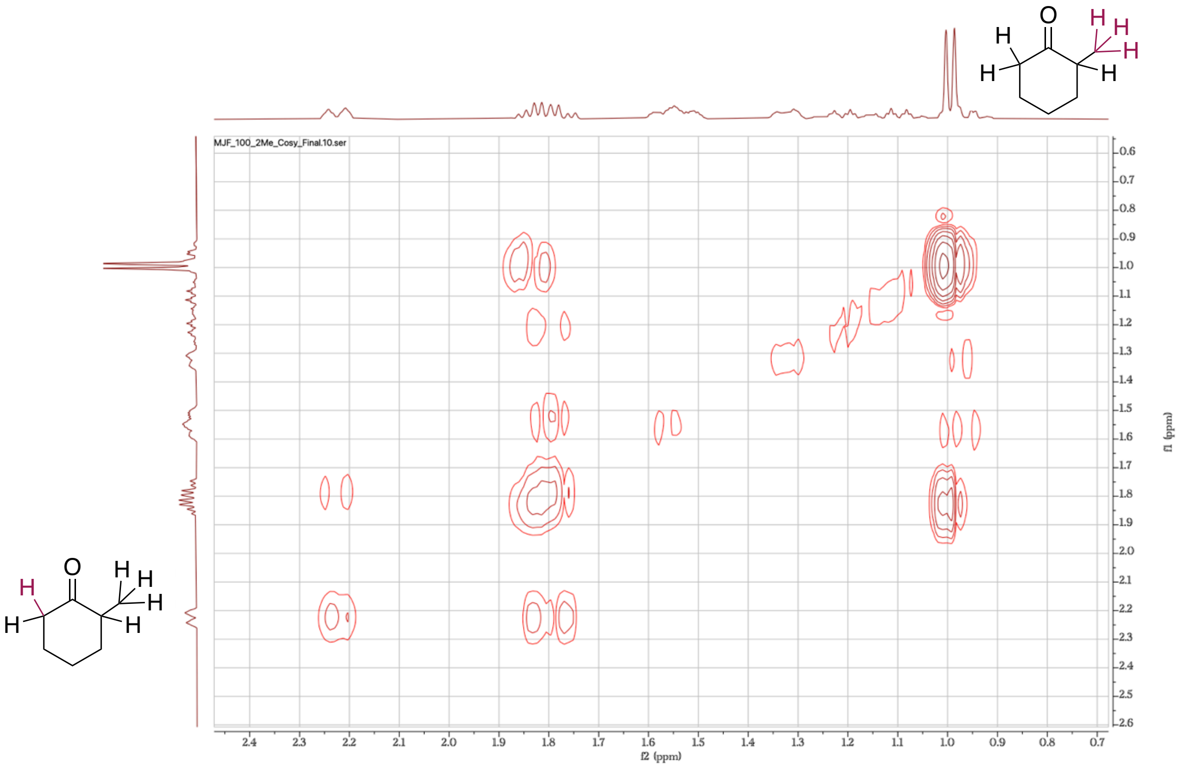

NMR and GC-MS Data Obtained From H/D Exchange Experiment Using Either Buffer Alone or Buffer + Cells. MW of non-deuterated ketone = 112 Da.

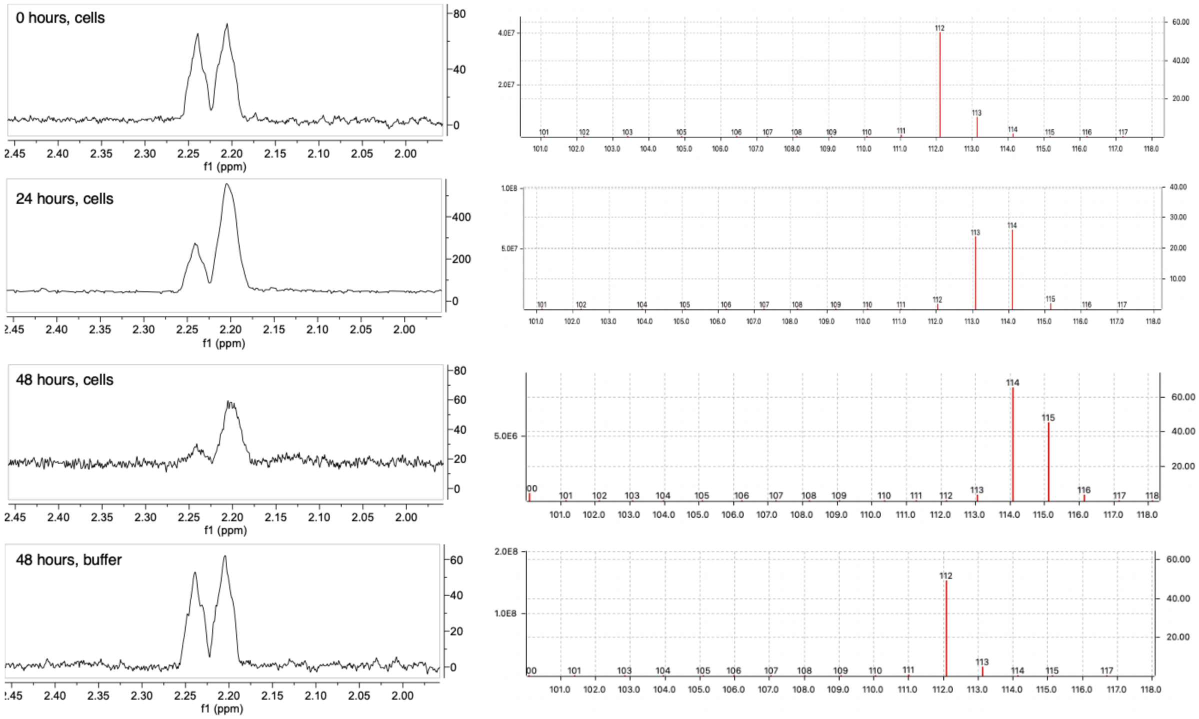

Summary of Findings from H/D Exchange Experiment

A.

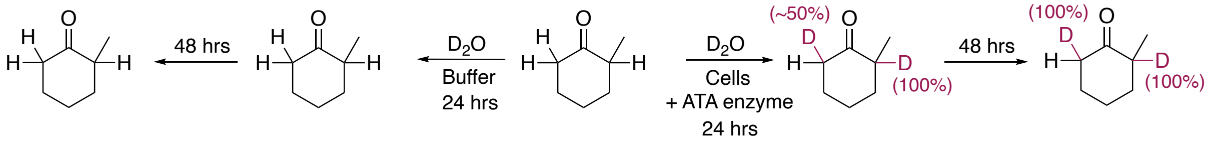

B.

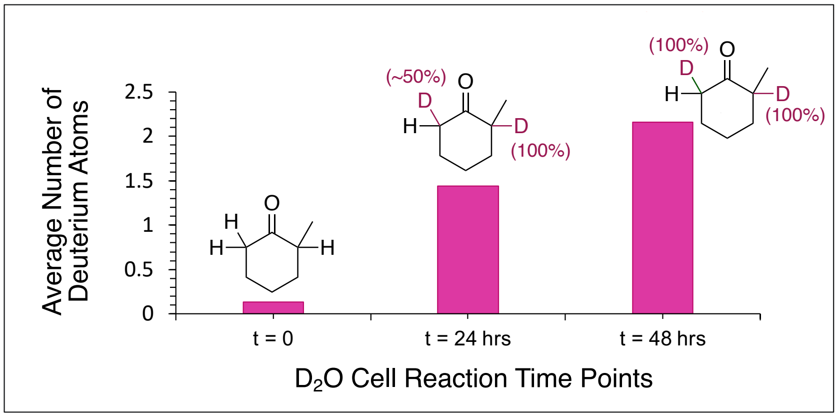

VI. Kinetics Studies: Elucidating the Mechanistic Details of the Reversible, Whole-Cell Transaminase Reaction

Experimental Overview

We conducted a series of NMR and UV-Vis experiments, using the 4-trifluoromethylcyclohexanone substrate. For all NMR experiments, the reactions were performed directly in an NMR tube, a set-up that provided comparable results to the standard reaction set-up.

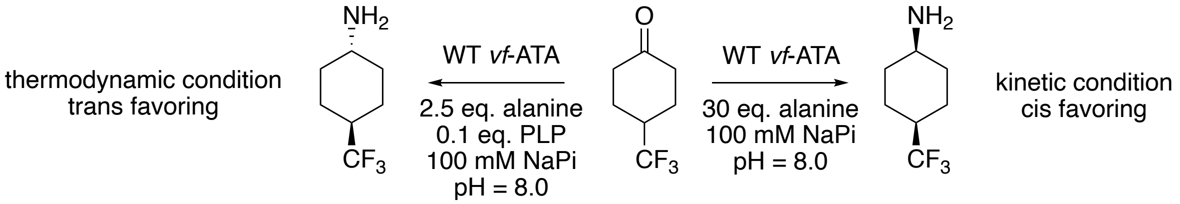

We began by analyzing the conversion of the ketone to the *cis* and *trans* amines, under both kinetic and thermodynamic favoring conditions, over the course of six hours . ^{19}F NMR was used for these experiments and the conversions were calculated from integrating the NMR peaks corresponding to each compound.

Next, we analyzed the same reaction, at the same time points using ^{13}C labeled alanine and ^{13}C NMR to track the consumption of alanine and the formation of pyruvate.

Finally, we analyzed the conversion of PLP to PMP and back to PLP under the thermodynamic condition. The kinetic condition does not contain additional extracellular PLP, and the differences in cellular PLP could not be monitored by UV-Vis. Both PLP and PMP are UV-active, enabling time-point aliquots to be analyzed using UV-Vis spectroscopy. Initial screening was performed to identify the wavelength of maximum absorbance for each substance: PLP $\lambda_{max} = 402$ and PMP $\lambda_{max} = 328$. These values were consistent with literature reports. An extinction coefficient of 5020 $M^{-1}cm^{-1}$ was used for PLP and 5191 $M^{-1}cm^{-1}$ for PMP. The PLP extinction coefficient was taken from the literature, while the PMP extinction coefficient was calculated under the assumption that the total concentration of PMP + PLP would remain constant throughout the reaction, as has been done in previous analyses investigating PLP dependent enzymes. From the absorbance spectra and the extinction coefficients, the concentration of PLP and PMP were calculated at each time point.

Detailed Methods for Kinetics Experiments

All kinetics experiments were performed using 4-trifluoromethylcyclohexanone as the ketone and using an aliquot of 80 mg/mL WT *Vf*-ATA cells, without pyridoxine hydrochloride.

Kinetics Experiments, ^{19}F NMR

The following reagents were added to standard NMR tubes, one per condition. **Kinetic condition, K:** Ketone (3.5 uL, 1.0 M), alanine (105 uL, 1.0 M), buffer (170 uL 100 mM NaPi, pH 8), D_2O (70 uL), and cells (350 uL, above aliquot). **Thermodynamic condition, T:** Ketone (3.5 uL, 1.0 M), alanine (9 uL, 1.0 M), buffer (235 uL 100 mM NaPi, pH 8), pyridoxal phosphate (35 uL, 10 mM), D_2O (70 uL), and cells (350 uL, above aliquot). A stopwatch was started immediately after adding the cells and the tubes were inverted to mix.

For each reaction (NMR tube), ^{19}F NMR with decoupling was performed with 256 scans and 90% H_2O and 10% D_2O solvent. NMR analysis was performed as soon as possible after the reaction started (usually two-three minutes due to practically of starting the NMR analysis), and at incremented time points over the

course of 24 hours The reaction under the kinetic condition was analyzed at the start and at 20 minutes, 40 minutes, 1 hour, 1.5 hours, 2 hours, 3 hours, 4 hours, 5 hours, 6 hours, and 24 hours. The reaction under the thermodynamic condition was analyzed at the start and at 30 minutes, 1 hour, 1.5 hours, 2 hours, 3 hours, 4 hours, 5 hours, 6 hours, and 24 hours. In between NMR time-points the reaction was incubated at room temperature, laying horizontally on a shaker to keep the cells suspended. Relative concentrations were calculated from integration values. These were transformed into absolute concentrations under the assumption that the total amine + ketone concentration was always equal to that of the original ketone concentration.

^{19}F NMR spectra, over the course of the reaction (0 – 24 hours):

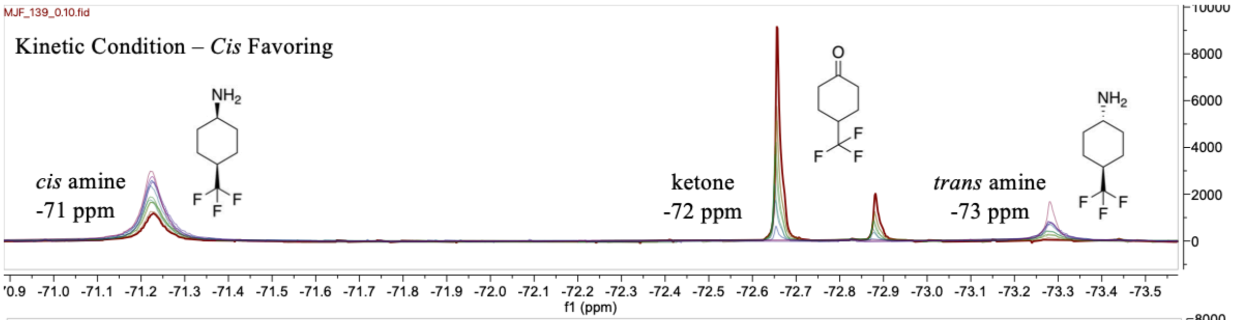

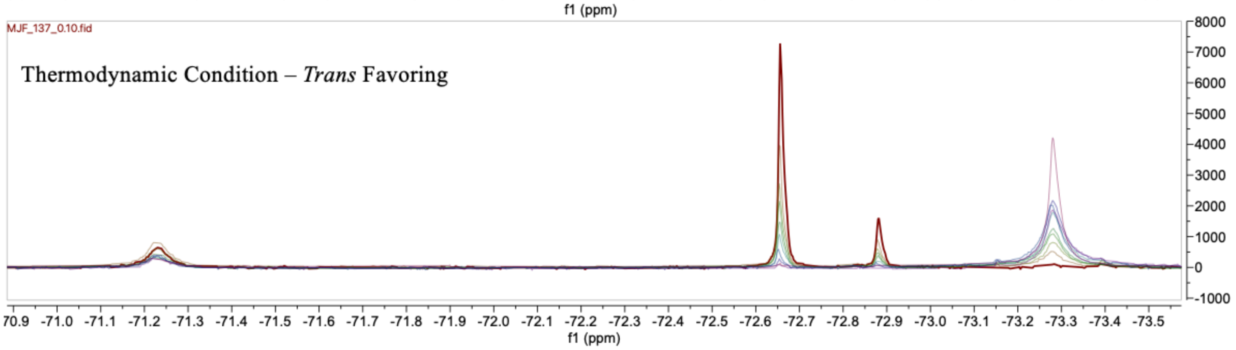

Kinetic condition, tabulated ^{19}F NMR data:

Time (h)	Ketone	Ketone mM	Cis Amine	Cis mM	Trans Amine	Trans mM
0.0791667	70%	3.5	30%	1.5	1%	0.03
0.33	54%	2.7	43%	2.1	3%	0.15
0.666	45%	2.2	49%	2.5	6%	0.31
1	39%	1.9	55%	2.8	6%	0.31
1.5	32%	1.6	59%	2.9	9%	0.46
2	25%	1.2	64%	3.2	12%	0.59
3	14%	0.72	71%	3.5	15%	0.75
4	4%	0.21	78%	3.9	18%	0.91
5	1%	0.03	81%	4.0	19%	0.93
6	0%	0.01	79%	4.0	20%	1.0
24	0%	0.00	79%	3.9	21%	1.1

Thermodynamic condition, tabulated ^{19}F NMR data:

Time (h)	Ketone	Ketone mM	Cis Amine	Cis mM	Trans Amine	Trans mM
0.1	79%	4.0	21%	1.1	0%	0.00
0.5	50%	2.5	33%	1.7	17%	0.85
1	41%	2.1	26%	1.3	32%	1.6
1.5	33%	1.7	19%	0.95	49%	2.5
2	25%	1.3	16%	0.80	59%	3.0
3	14%	0.70	11%	0.55	75%	3.8
4	8%	0.40	11%	0.55	81%	4.1
5	2%	0.10	11%	0.55	87%	4.4
6	3%	0.15	10%	0.50	87%	4.4
24	2%	0.10	9%	0.45	89%	4.5

Kinetics Experiments, ^{13}C NMR:

The following reagents were added to standard NMR tubes, one per condition. **Kinetic condition, K:** Ketone (3.5 uL, 1.0 M), ^{13}C alanine (105 uL, 1.0 M), buffer (170 uL 100 mM NaPi, pH 8), D_2O (70 uL), and cells (350 uL, above aliquot). **Thermodynamic condition, T:** Ketone (3.5 uL, 1.0 M), ^{13}C alanine (9 uL, 1.0 M), buffer (235 uL 100 mM NaPi, pH 8), pyridoxal phosphate (35 uL, 10 mM), D_2O (70 uL), and cells (350 uL, above aliquot). A stopwatch was started immediately after adding the cells and the tubes were inverted to mix.

For each reaction (NMR tube), ^{13}C NMR with decoupling was performed with 28 scans with 90% H_2O and 10% D_2O solvent. NMR analysis was performed as soon as possible after the reaction started (usually two-three minutes), and at incremented time points over the course of 24 hours. The reaction under the kinetic condition was analyzed at the start and at 20 minutes, 40 minutes, 1 hour, 1.5 hours, 2 hours, 3 hours, 4 hours, 5 hours, 6 hours, and 24 hours. The reaction under the thermodynamic condition was analyzed at the start and at 30 minutes, 1 hour, 1.5 hours, 2 hours, 3 hours, 4 hours, 5 hours, 6 hours, and 24 hours. In between NMR time-points the reaction was incubated at room temperature, laying horizontally on a shaker to keep the cells suspended. The concentrations of alanine, pyruvate and bicarbonate were calculated overtime. DMSO was used as an internal standard for integration.

NMR spectra, over the course of the reaction (0 – 24 hours):

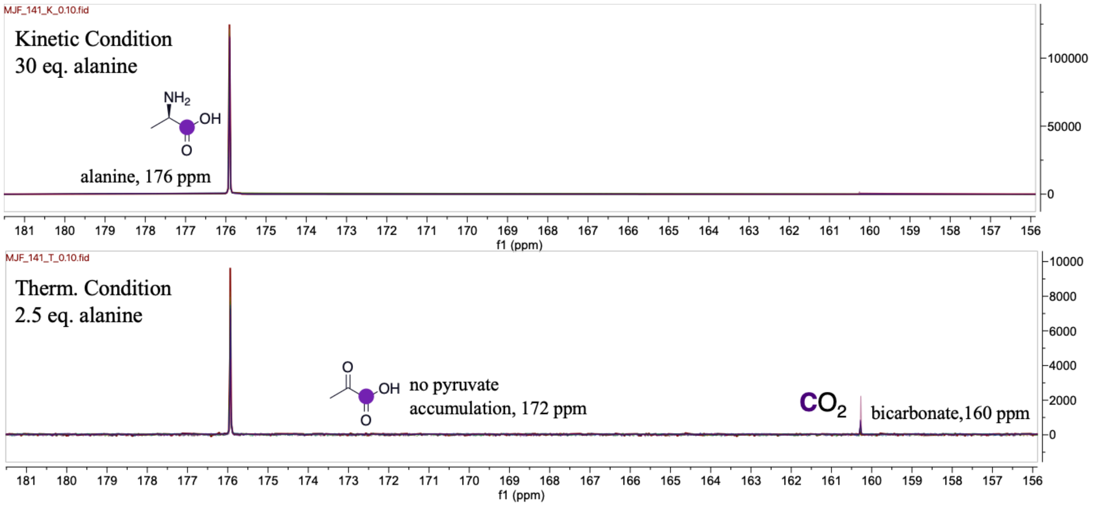

Kinetic condition, tabulated ^{13}C NMR data

Time Point (h)	Alanine	Alanine mM	Bicarbonate	Bicarbonate mM
0.124	100	150	24	0.4
0.33	98	149	36	0.6
0.66	91	149	41	0.7
1	88	149	44	0.8
1.5	82	149	48	0.9
2	79	149	52	1.1
3	77	149	55	1.1
4	76	149	57	1.2
5	73	149	59	1.3
6	72	149	58	1.3
24	65	148	100	2.4

Thermodynamic condition, tabulated ^{13}C NMR data:

Time Point (h)	Alanine	Alanine mM	Bicarbonate	Bicarbonate mM
0.065	100	12	12	0.2
0.5	87	12	27	0.6
1	84	12	31	0.7
1.5	82	12	37	0.8
2	78	12	38	0.9
3	69	11	40	1.0
4	67	11	43	1.1
5	66	11	46	1.2
6	63	11	54	1.4
24	35	9	100	3.8

Kinetics Experiments, UV-Vis Assay

UV-Vis assays were performed to track the relevant concentration of PLP and PMP, both UV-active reagents, overtime. The following reagents were added to 20 mL glass vials, one per condition. **Kinetic condition, K:** Ketone (40 uL, 1.0 M), alanine (1.20 mL, 1.0 M), buffer (2.76 mL 100 mM NaPi, pH 8), and cells (4 mL, above aliquot). **Thermodynamic condition, T:** Ketone (40 uL, 1.0 M), alanine (100 uL, 1.0 M), buffer (3.460 mL 100 mM NaPi, pH 8), pyridoxal phosphate (400 uL, 10 mM), and cells (4 mL, above aliquot). A stopwatch was started immediately after adding the cells.

For each reaction, UV-Vis analysis was performed as soon as possible after the reaction started (usually two-three minutes), and at incremented time points over the course of 24 hours. The reaction under the kinetic condition was analyzed at the start and at 20 minutes, 40 minutes, 1 hour, 1.5 hours, 2 hours, 3 hours, 4 hours, 5 hours, 6 hours, and 24 hours. The reaction under the thermodynamic condition was analyzed at the start and at 30 minutes, 1 hour, 1.5 hours, 2 hours, 3 hours, 4 hours, 5 hours, 6 hours, and 24 hours. In between time-points the reaction was incubated at room temperature, in a shaker to keep the cells suspended.

At the indicated time points, 700 uL aliquots were removed from the reaction and transferred to a 1.5 mL centrifuge vial. The vial was centrifuged for two minutes at 10,000 rpm and the top layer was transferred to a plastic cuvette (path length 1 cm) for analysis. All UV-Vis analysis was performed on a Agilent Technologies Cary 8454 UV-Vis spectrometer with a tungsten-deuterium lamp over the complete

visible range. Prior to analysis of each sample, a blank sample of buffer (100 mM NaPi, pH 8) was analyzed. A calibration curve was obtained and later used to quantify the concentrations of PLP ($\lambda_{max} = 402$) and PMP ($\lambda_{max} = 328$) in each sample. An extinction coefficient of 5020 $M^{-1}cm^{-1}$ was used for PLP and 5191 $M^{-1}cm^{-1}$ for PMP. The PLP extinction coefficient was taken from the literature, while the PMP extinction coefficient was calculated under the assumption that the total concentration of PMP + PLP would remain constant throughout the reaction. [33]

Thermodynamic condition, tabulated UV-Vis data:

Time Point	Abs 328 nm	Conc Cuvette (mM)	Conc Reaction (mM)	Abs 402 nm	Conc Cuvette (mM)	Conc Reaction (mM)
0.5	0.553	0.107	0.266	0.754	0.150	0.376
1	0.517	0.100	0.249	0.637	0.127	0.317
1.5	0.614	0.118	0.296	0.656	0.131	0.326
2	0.629	0.121	0.303	0.593	0.118	0.295
4	0.728	0.140	0.351	0.544	0.108	0.271
5	0.788	0.152	0.379	0.509	0.101	0.253
6	0.868	0.167	0.418	0.535	0.107	0.266
24	1.038	0.200	0.460	0.081	0.016	0.040

A.

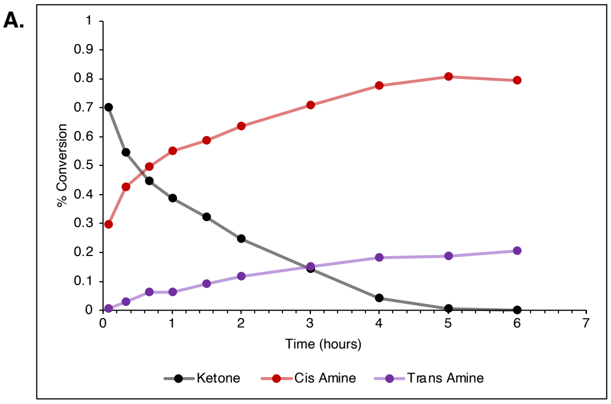
% Conversion
Time (hours)
Ketone
Cis Amine
Trans Amine

B.

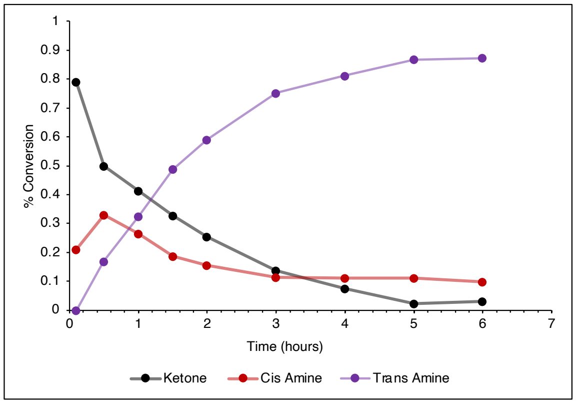
% Conversion
Time (hours)
Ketone
Cis Amine
Trans Amine

C.

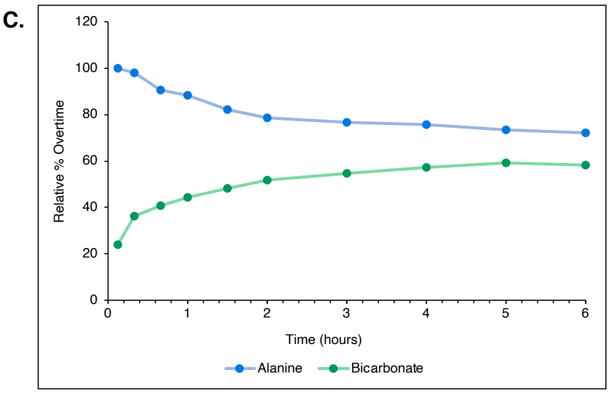
Relative % Overtime
Time (hours)
Alanine
Bicarbonate

D.

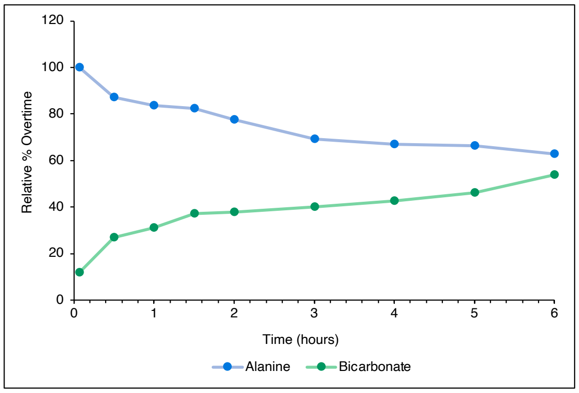
Relative % Overtime
Time (hours)
Alanine
Bicarbonate

E.

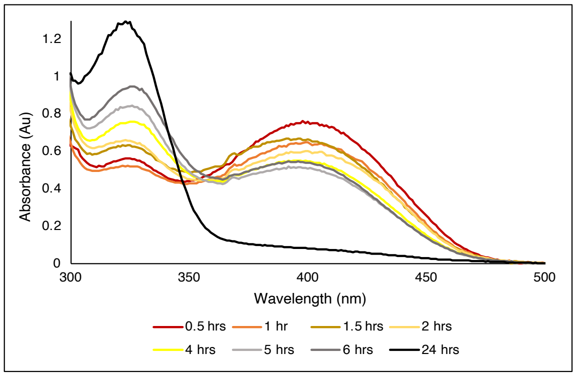
Absorbance (Au)
Wavelength (nm)
0.5 hrs
1 hr
1.5 hrs
2 hrs
4 hrs
5 hrs
6 hrs
24 hrs

F.

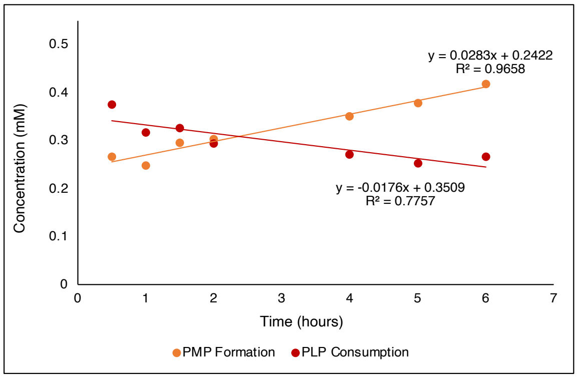
y = 0.0283x + 0.2422
R² = 0.9658
y = -0.0176x + 0.3509
R² = 0.7757
Concentration (mM)
Time (hours)
PMP Formation
PLP Consumption

A.

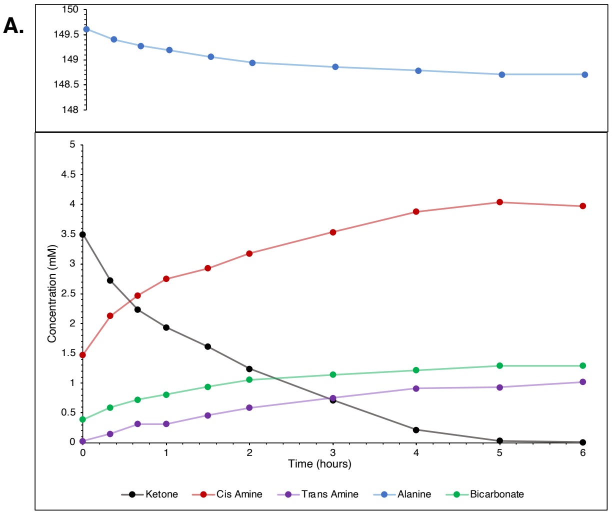
Concentration (mM)
Time (hours)
Ketone
Cis Amine
Trans Amine
Alanine
Bicarbonate

B.

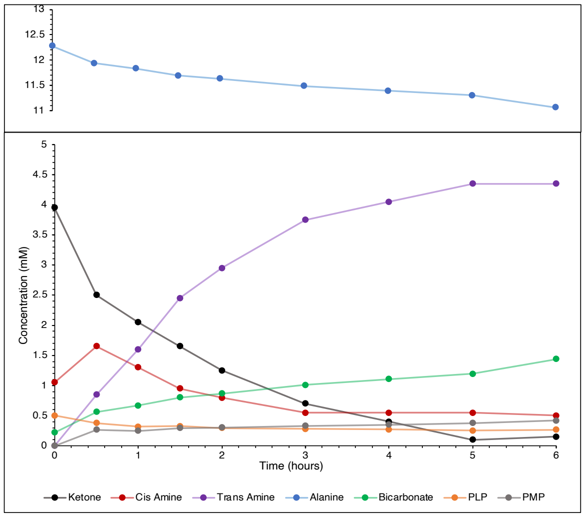
Concentration (mM)
Time (hours)
Ketone
Cis Amine
Trans Amine
Alanine
Bicarbonate
PLP
PMP

VII. Data from ATA Reactions Conducted on Analytical Scale

4-Methylcyclohexanone (1A)

GC Data: Top. Commercial *trans*-4-methylcyclohexamine. Middle. Kinetic condition, *cis* favoring: 30 eq alanine, WT without pyridoxine hydrochloride, 6 hours. Bottom. Thermodynamic condition, *trans* favoring: 2.5 eq alanine, 0.1 eq PLP, WT without pyridoxine hydrochloride, 24 hours.

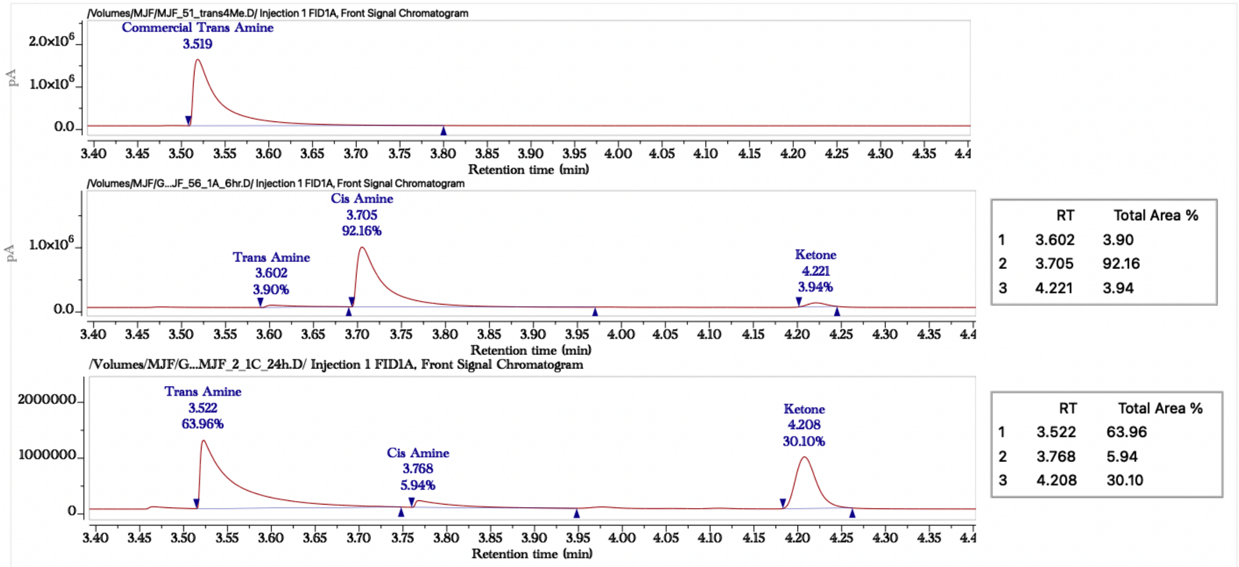

GC-MS Data: Top. Kinetic condition, Bottom. thermodynamic condition.

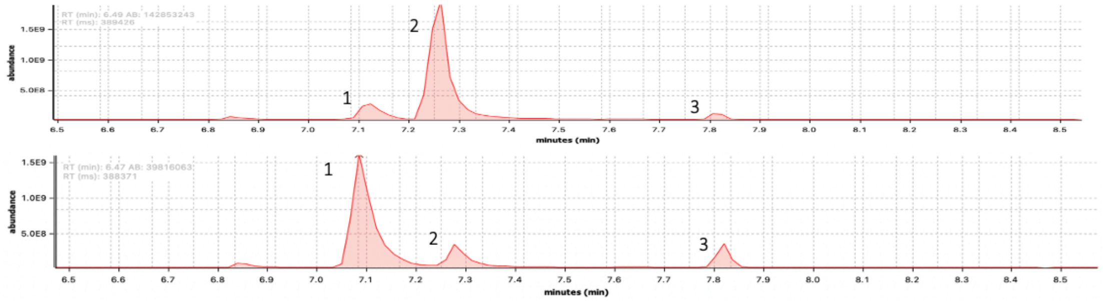

GC-MS Peak 1: *Trans* Amine. MW = 113.

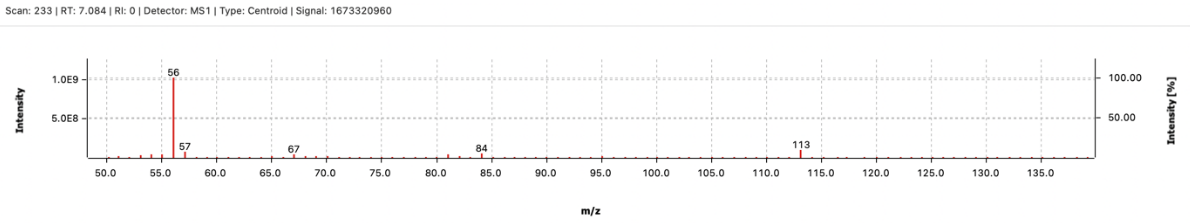

GC-MS Peak 2: *Cis* Amine. MW = 113.

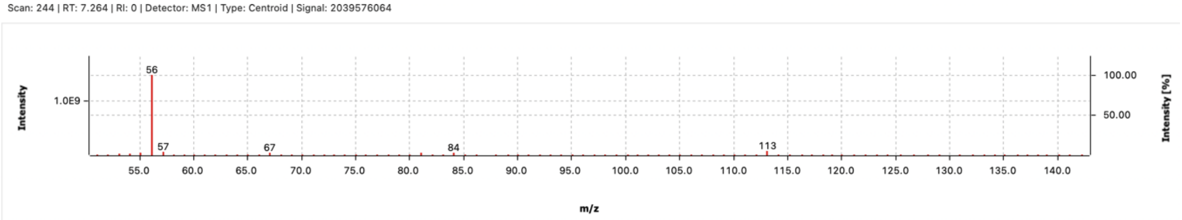

GC-MS Peak 3: Ketone. MW = 112.

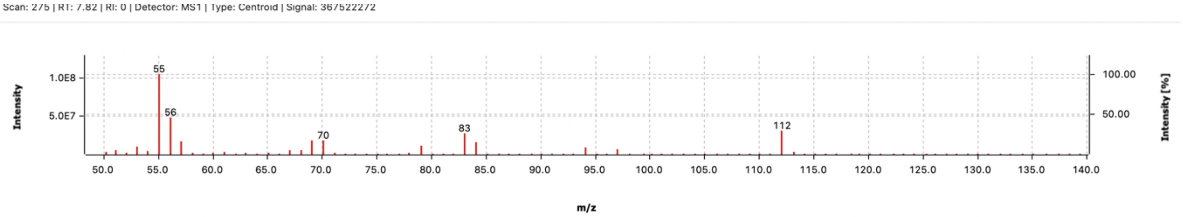

Assignment of Stereochemistry: GC was used to compare the retention time of the commercially available *trans*-4-methylcyclohexylamine to the retention time of the products of the reaction.

4-Ethylcyclohexanone (2A)

GC Data: Top: Kinetic condition, *cis* favoring: 30 eq alanine, WT without pyridoxine hydrochloride, 6 hours. Bottom: Thermodynamic condition, *trans* favoring: 2.5 eq alanine, 0.1 eq PLP, WT without pyridoxine hydrochloride, 24 hours.

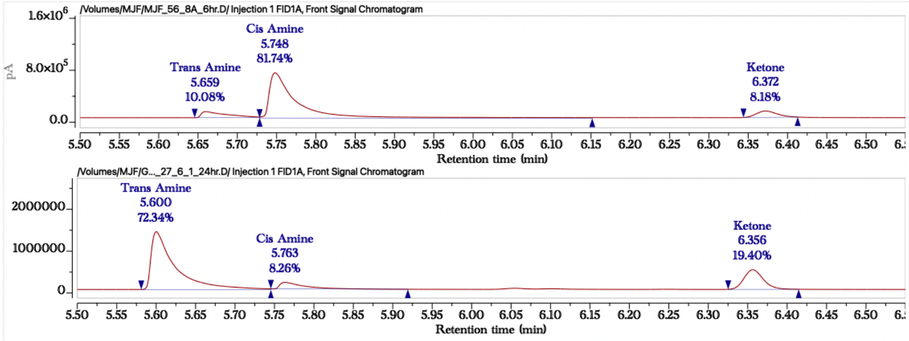

	RT	Total Area %
1	5.659	10.08
2	5.748	81.74
3	6.372	8.18

	RT	Total Area %
1	5.600	72.34
2	5.763	8.26
3	6.356	19.40

GC-MS Data: Top. Kinetic condition, Bottom. Thermodynamic condition.

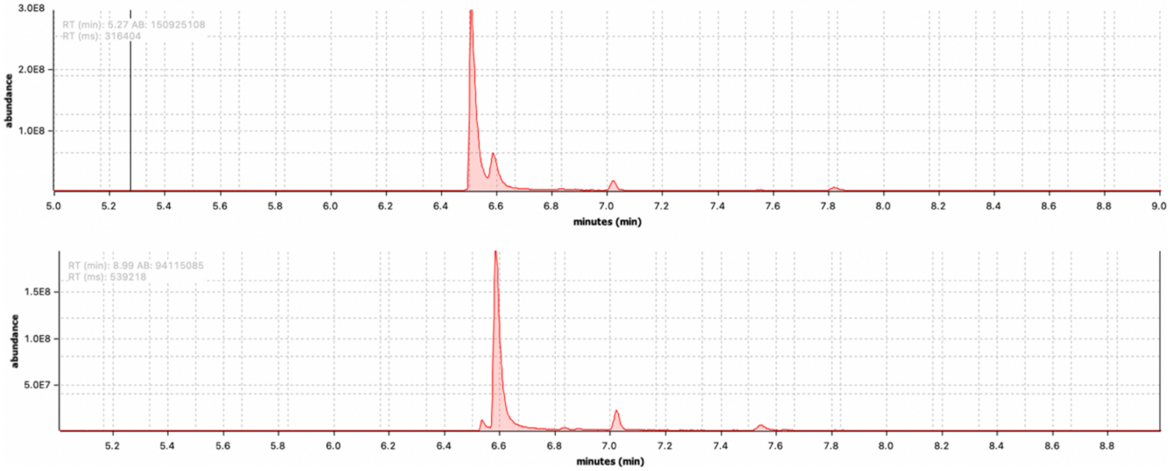

GC-MS: Peak 1: *Cis* amine. MW = 127.

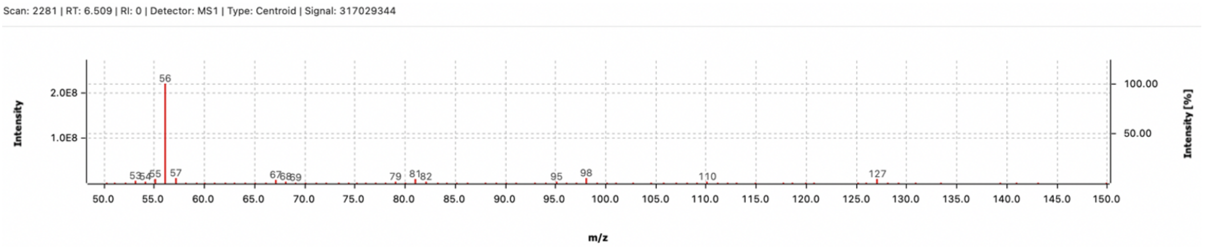

GC-MS: Peak 2: *Trans* amine. MW = 127.

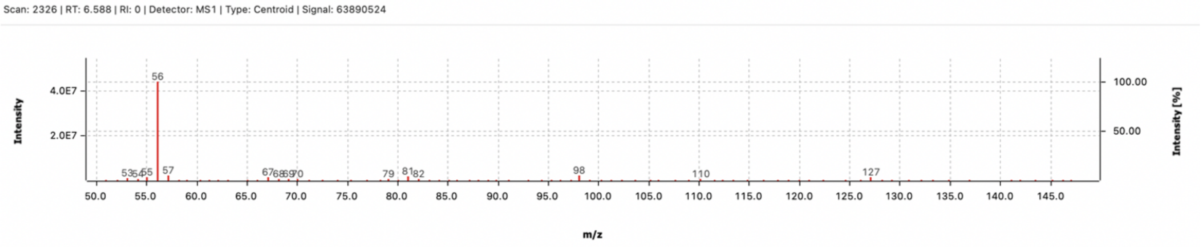

GC-MS: Peak 3: Ketone. MW = 126.

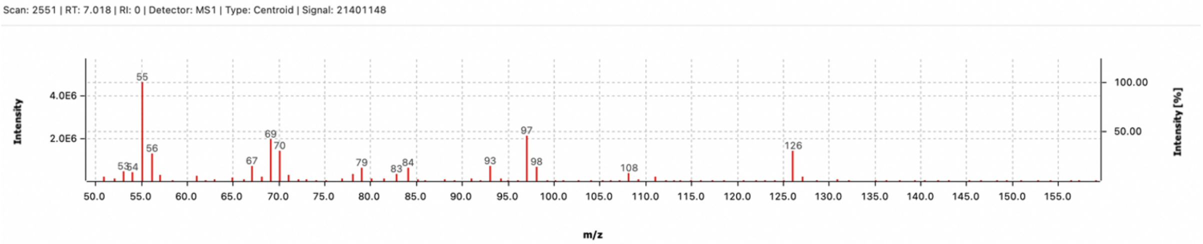

Assignment of Stereochemistry: Crude extracts of the reactions under conditions proposed to be *cis* and *trans* favoring were analyzed by 1H NMR. As observed in the methyl, phenyl, and tert-butyl substrates, in the *cis* isomer, the proton ipso to the amine has a higher chemical shift and a small coupling constant than the same proton in the *trans* isomer, with a difference in chemical shift of ~0.5ppm. This same pattern was observed in the case of the products from the ethyl amine. Therefore, by analogy, we assigned the *trans* isomer and *cis* isomer of the 4-ethylcyclohexylamine.

Assignment of Stereochemistry using the ^{1}H NMR signal of ipso hydrogen as observed in the ^{1}H crude reaction: (C_6D_6). Top. Kinetic condition, Bottom. Thermodynamic condition.

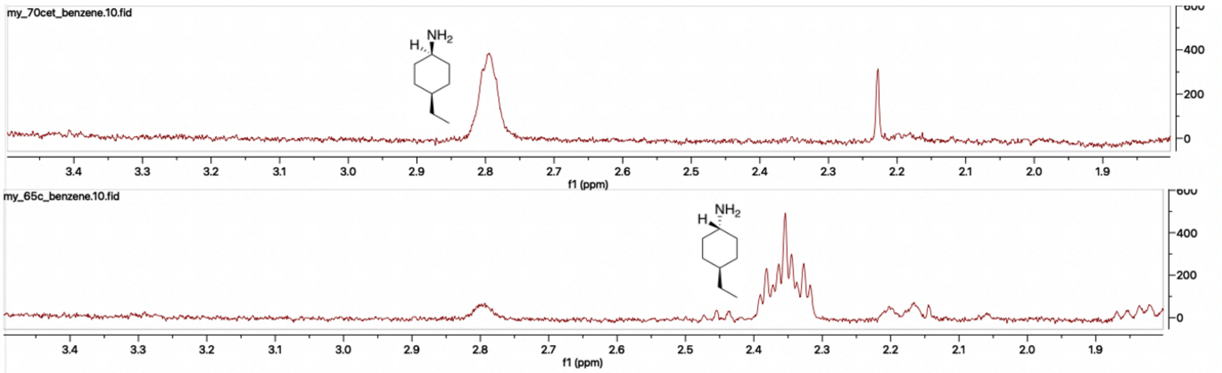

4-Isopropylcyclohexanone (6A)

GC Data: Top. Kinetic condition, *cis* favoring: 30 eq alanine, WT with pyridoxine hydrochloride, 24 hours. Middle. Thermodynamic condition, *trans* favoring: 3.5 eq alanine, 0.1 eq PLP, WT with pyridoxine hydrochloride, 48 hours. Bottom. Optimized thermodynamic condition, *trans* favoring: 3.5 eq alanine, 0.1 eq PLP, I259V mutant, 24 hours.

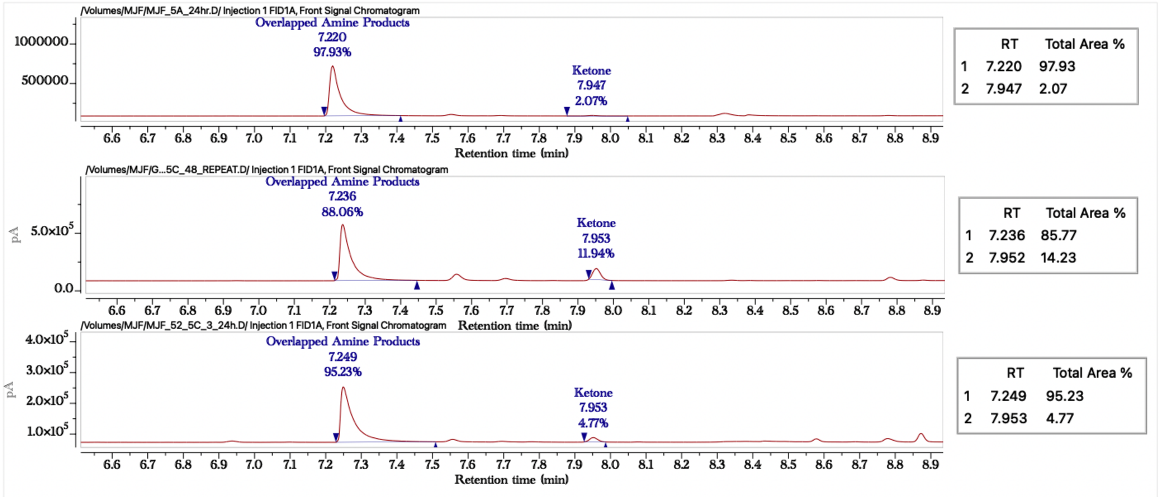

GC-MS Data: 1. Kinetic condition, 2. Thermodynamic condition.

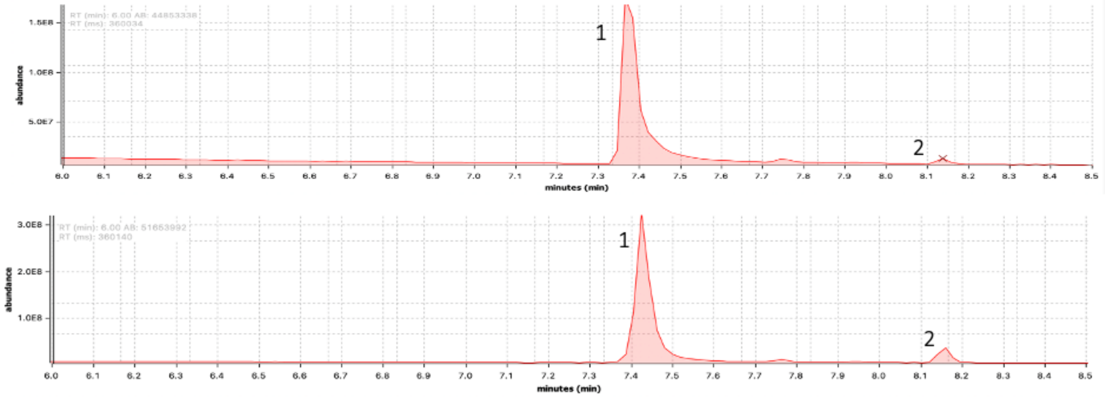

GC-MS Peak 1: Overlapped Amine Products. MW = 141.

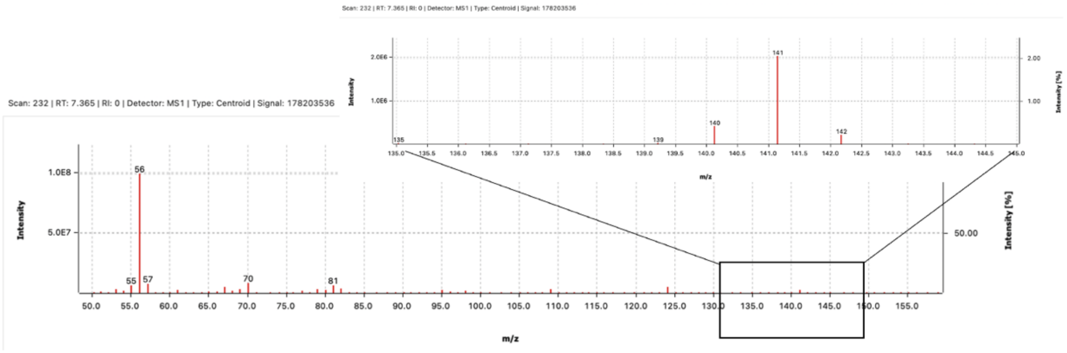

GC-MS Peak 2. Ketone. MW = 140.

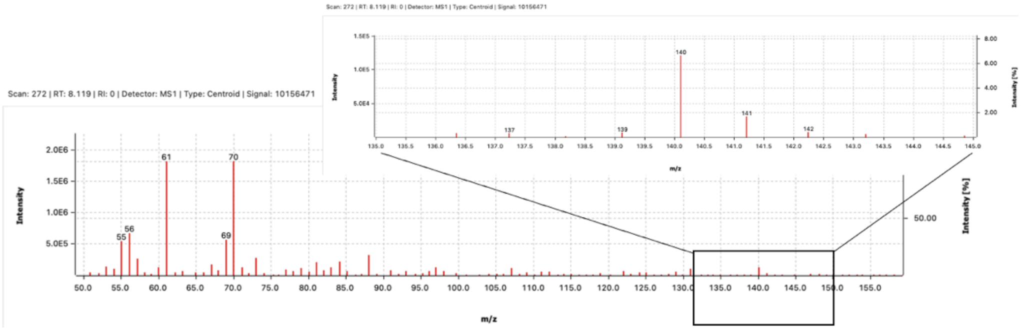

Derivatized reactions of 4-iPr Cyclohexanone:

GC Data: Top. Kinetic condition, *cis* favoring: 30 eq alanine, WT with pyridoxine hydrochloride, 24 hours. Middle. Thermodynamic condition, *trans* favoring: 3.5 eq alanine, 0.1 eq PLP, WT with pyridoxine hydrochloride, 48 hours. Bottom. Optimized thermodynamic condition, *trans* favored: 3.5 eq alanine, 0.1 eq PLP, I259V mutant, 24 hours.

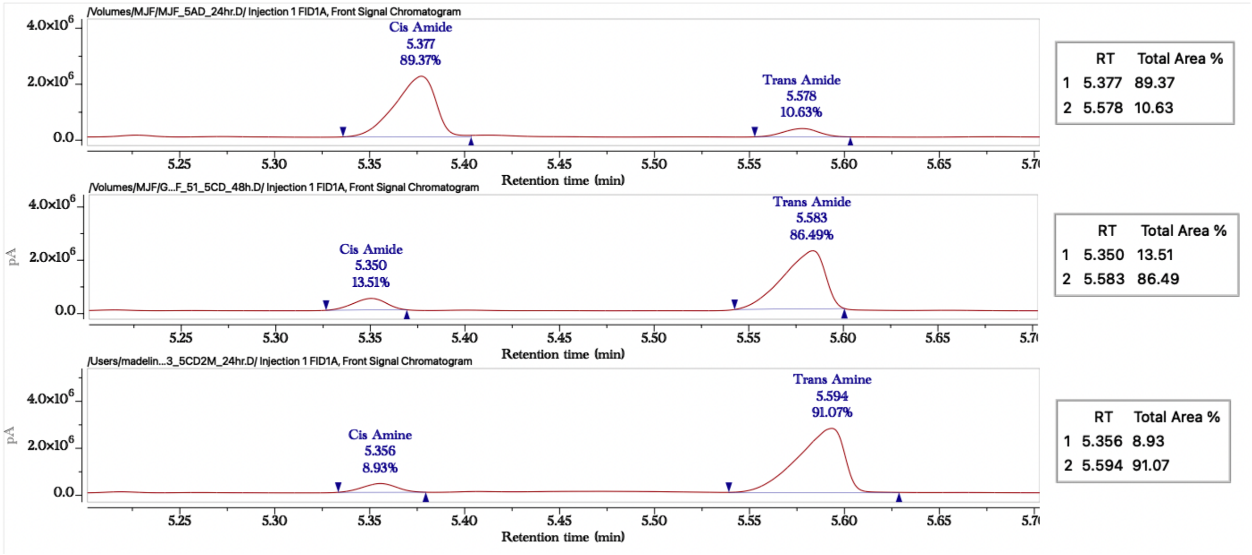

GCMS Data: Top: Kinetic condition, Bottom: Thermodynamic condition.

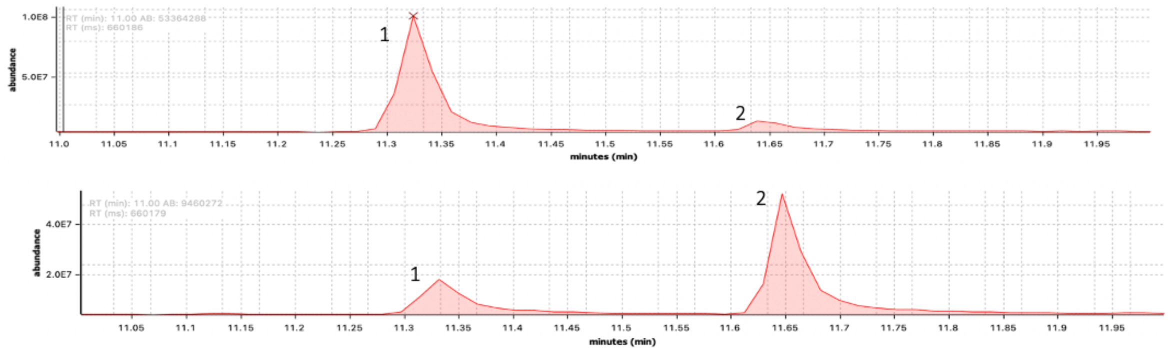

Peak 1: *Cis* Amide. MW = 183.

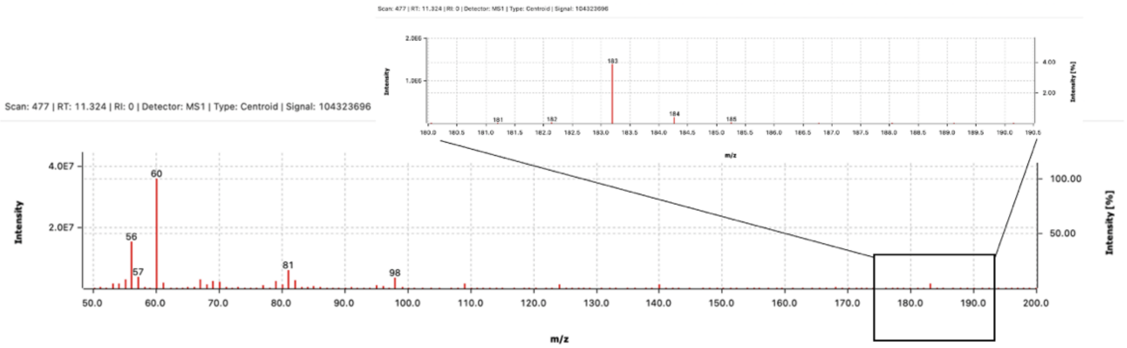

Peak 2: *Trans* Amide. MW = 183.

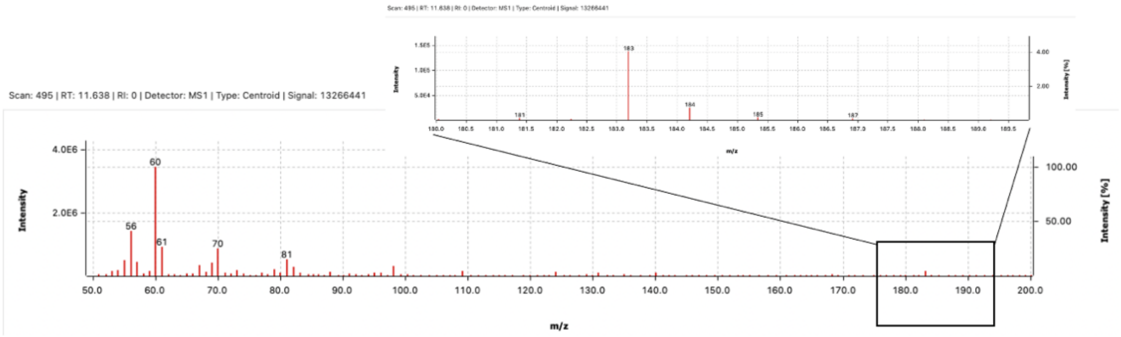

Assignment of Stereochemistry: Crude extracts of the reactions under conditions proposed to be *cis* and *trans* favoring were analyzed by 1H NMR. As observed in the methyl, phenyl, and tert-butyl substrates, in the *cis* isomer, the proton ipso to the amine has a higher chemical shift and a small coupling constant than the same proton in the *trans* isomer, with a difference in chemical shift of ~0.5ppm. This same pattern was observed in the case of the products from the isopropyl amine. Therefore, by analogy, we assigned the *trans* isomer and *cis* isomer of the 4-isopropylcyclohexylamine.

Assignment of Stereochemistry using the ^{1}H NMR signal of ipso hydrogen as observed in the ^{1}H crude reaction: (C_6D_6). Top. Kinetic condition, Bottom. Thermodynamic condition.

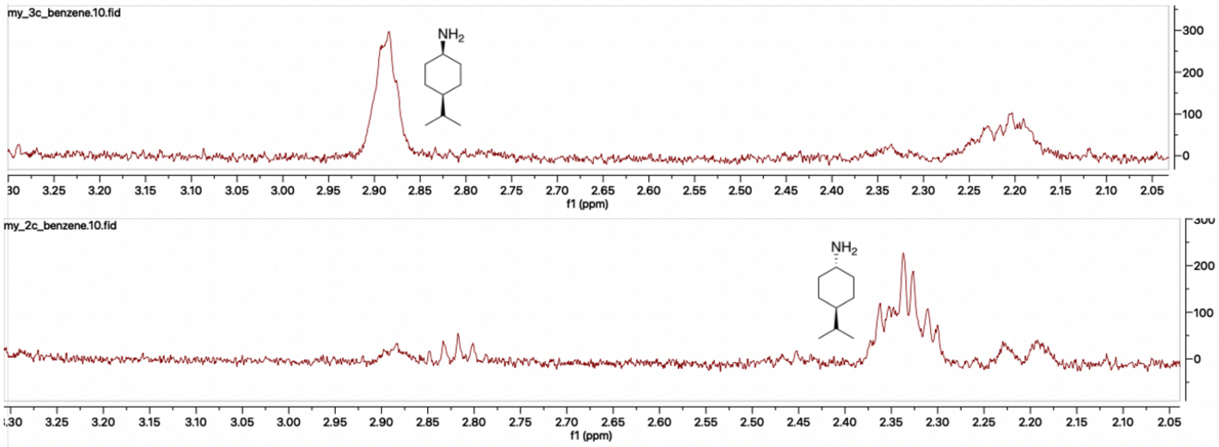

4-Trifluoromethylcyclohexanone (9A)

GC Data: Top. Kinetic condition, *cis* favoring: 30 eq alanine, WT without pyridoxine hydrochloride, 1.5 hours. Bottom. Thermodynamic condition, *trans* favoring: 2.5 eq alanine, 0.1 eq PLP, WT with pyridoxine hydrochloride, 6 hours.

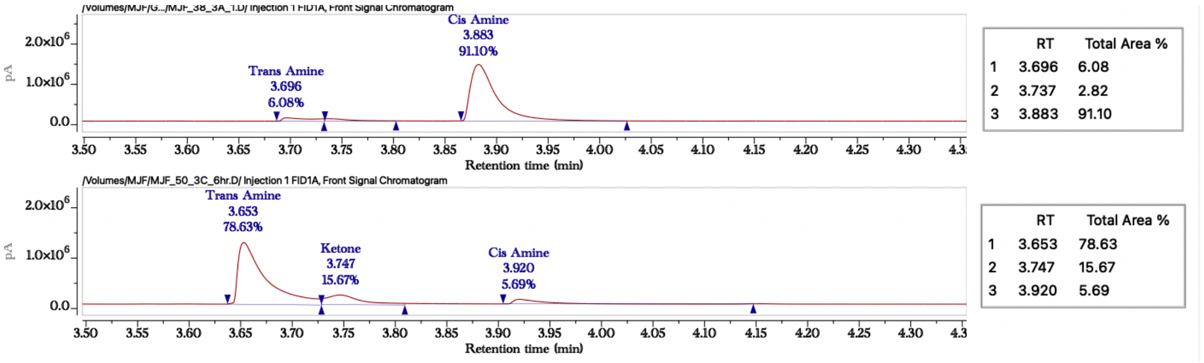

GC-MS Data: 1. Kinetic condition. 2. Thermodynamic condition.

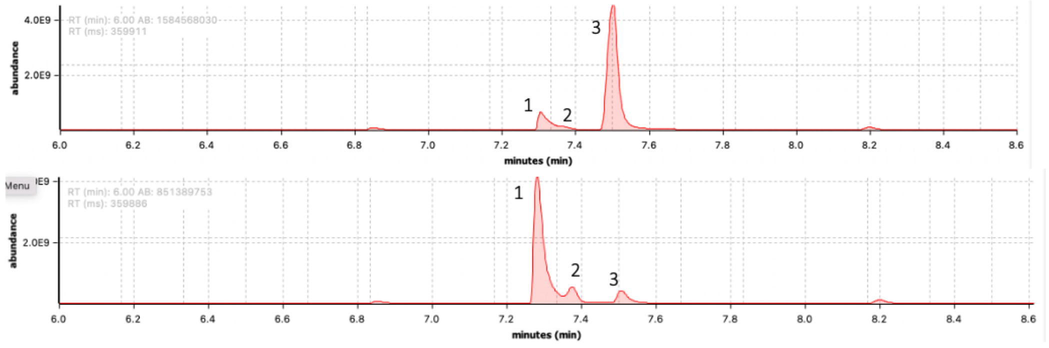

GC-MS Peak 1: *Trans* Amine. MW = 167.

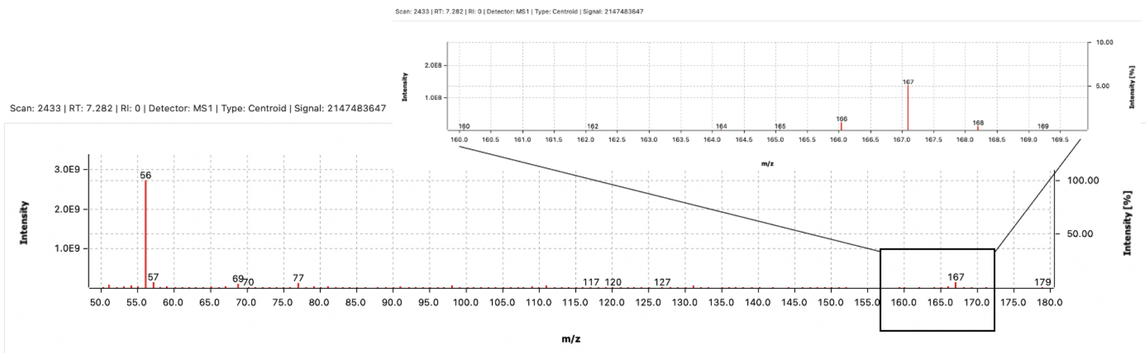

GC-

GC-MS Peak 2: Ketone. MW = 166.

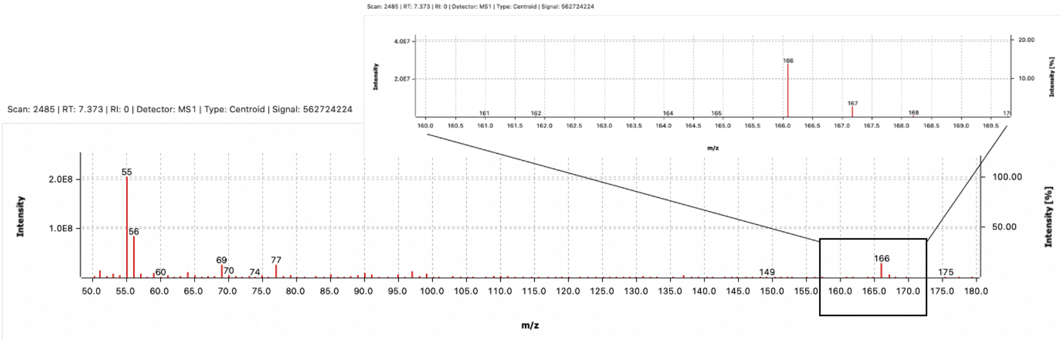

GC-MS Peak 3: *Cis* Amine. MW = 167.

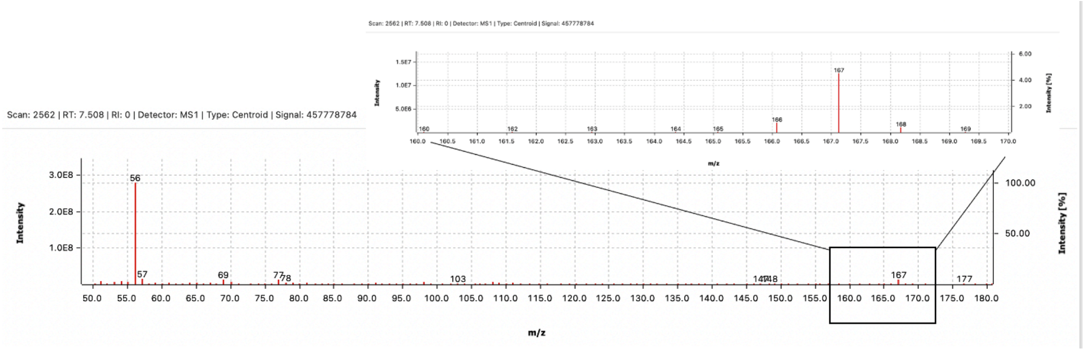

Derivatized Reactions of 4-CF_3 Cyclohexanone:

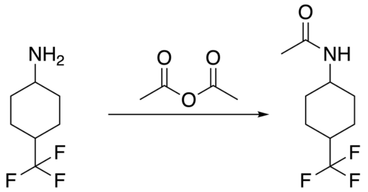

GC Data: Top. Kinetic condition, *cis* favoring: 30 eq alanine, WT without pyridoxine hydrochloride, 1.5 hours. Bottom. Thermodynamic condition, *trans* favoring: 2.5 eq alanine, 0.1 eq PLP, WT with pyridoxine hydrochloride, 6 hours.

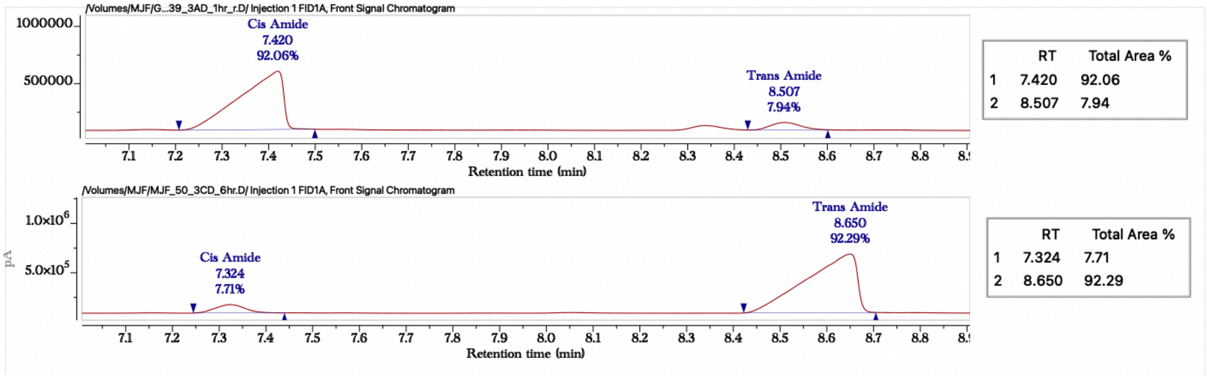

GC-MS Data: Top. Kinetic condition. Bottom. Thermodynamic condition.

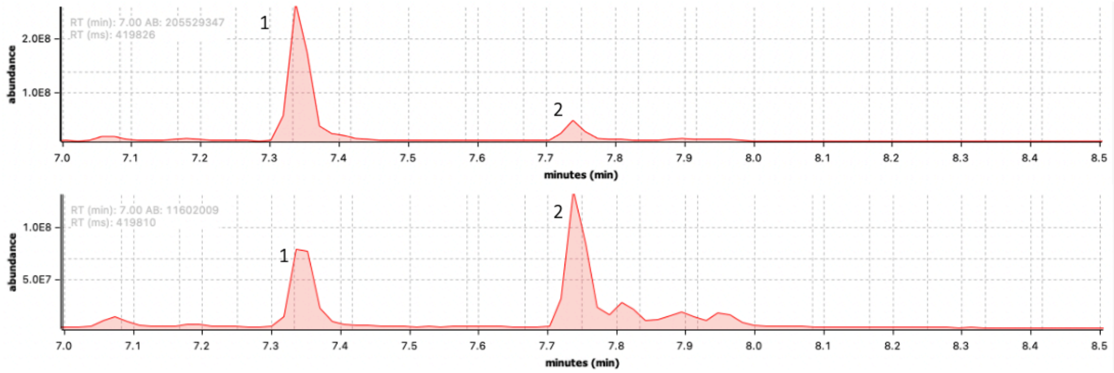

GC-MS Peak 1. *Cis* Amide. MW = 209.

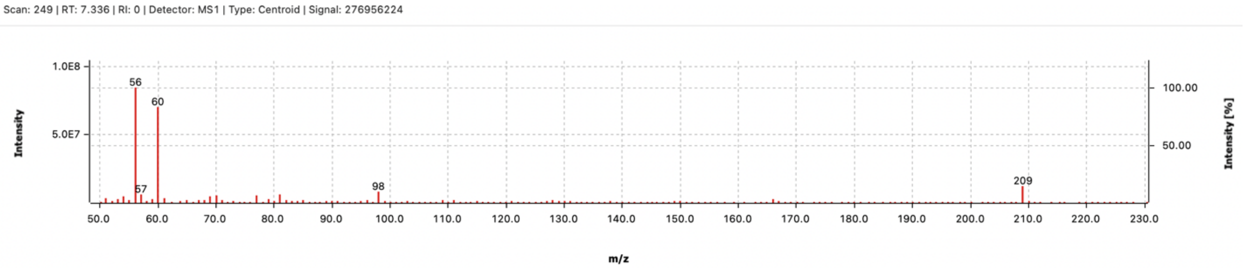

GC-MS Peak 2. *Trans* Amide. MW = 209.

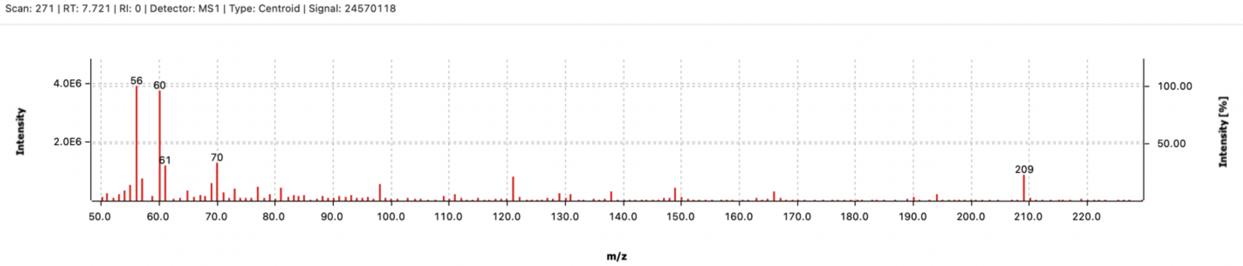

Assignment of Stereochemistry using the ^{19}F NMR signal of the CF_3 as observed in the ^{19}F crude reaction: (C_6D_6). Top. Kinetic condition, Bottom. Thermodynamic condition.

^{19}F NMR: (10% D_2O, 90% H_2O). Top. Kinetic condition. Bottom. Thermodynamic condition.

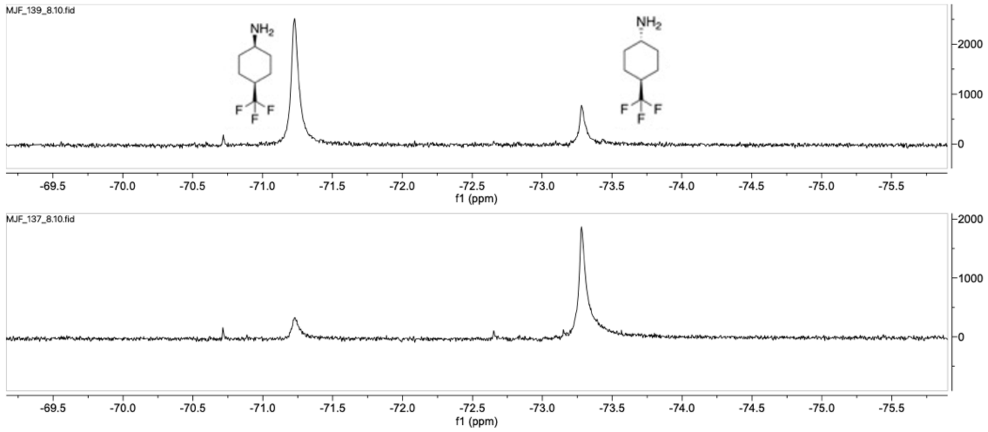

4-Isopropoxycyclohexanone (5A)

GC Data: Top. Kinetic condition, *cis* favoring: 30 eq alanine, WT without pyridoxine hydrochloride, 24 hours. Bottom. Thermodynamic condition, *trans* favoring: 2.5 eq alanine, 0.1 eq PLP, WT without pyridoxine hydrochloride, 72 hours.

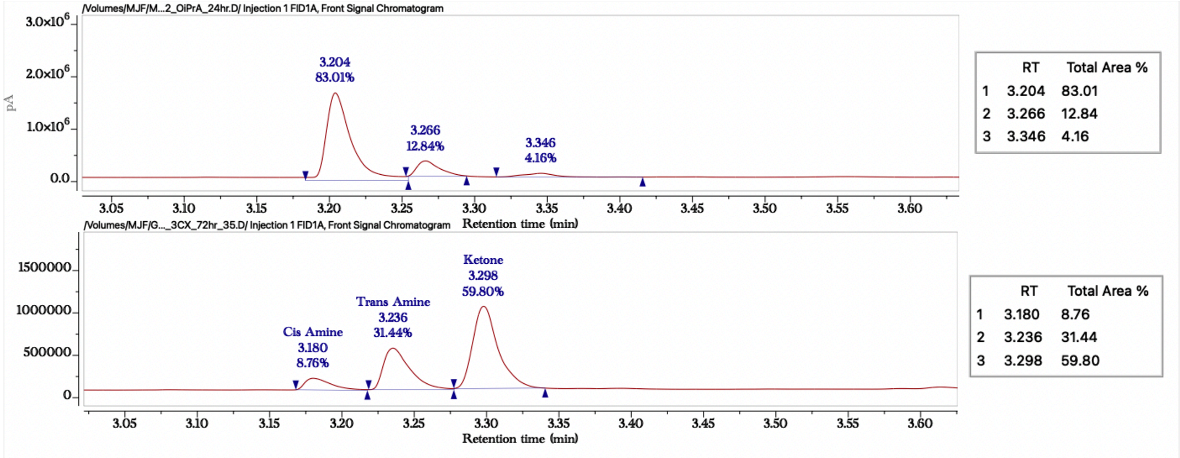

GC-MS Data: Top. Kinetic condition. Bottom. Thermodynamic condition.

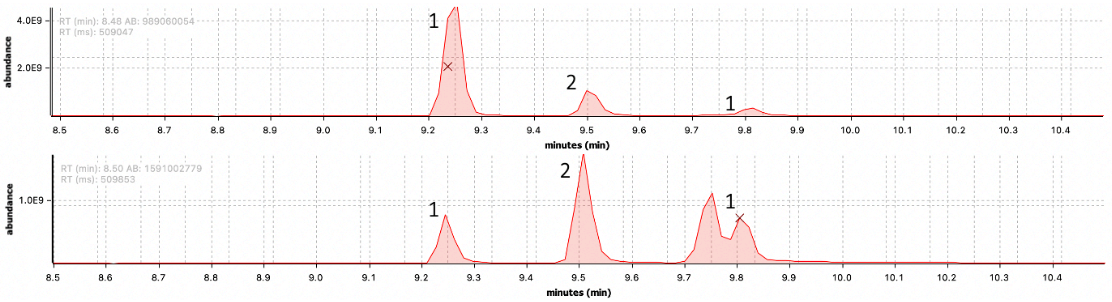

Peak to the left of Peak 1 (ketone) determined to be an alcohol byproduct produced by the cells.

GC-MS Peak 1: *Cis* Amine. MW = 157.

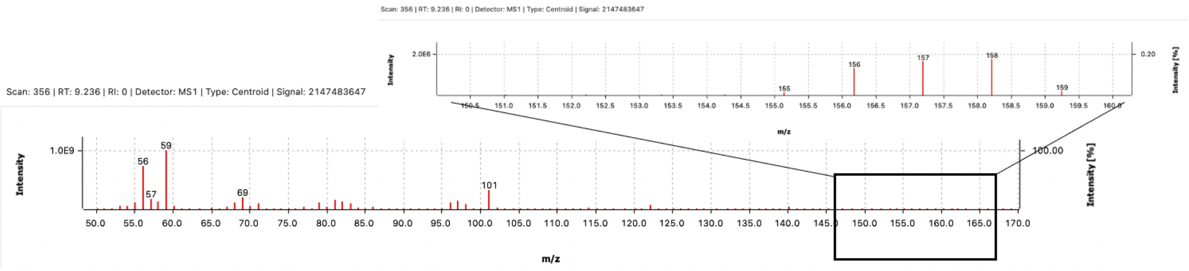

GC-MS Peak 2. *Trans* Amine (3g). MW = 157.

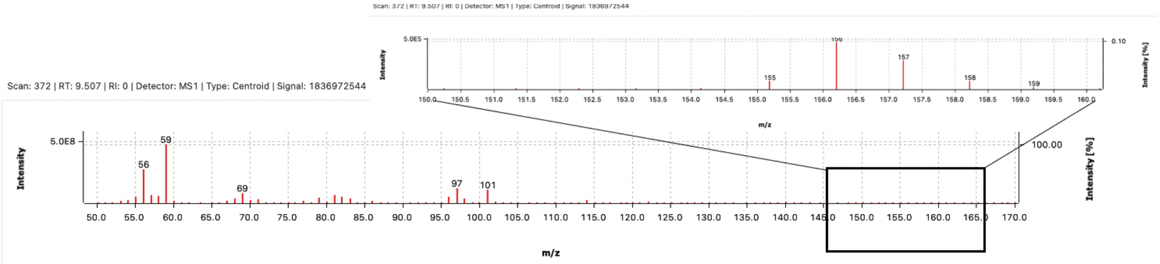

GC-MS Peak 3. Ketone (1g). MW = 156.

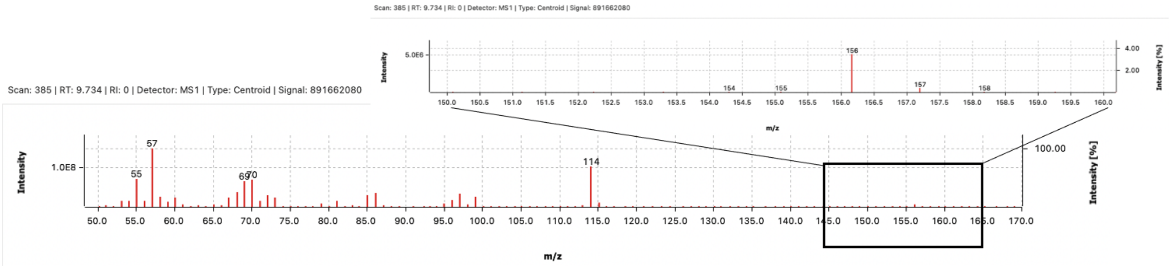

Assignment of Stereochemistry: The products of the proposed *trans* favoring reaction were analyzed by 1H NMR. Literature data on the synthesis of *trans*-4-isopropoxycyclohexanamine reported the 1H multiplet ~3.25 ppm, which was present in our sample. The coupling constant and lower chemical shift of this *trans* signal were consistent with our previous observations of 4-substituted cyclohexylamines. Analysis of the proposed *cis* favoring reaction revealed a major signal at ~3.50 ppm, which did not match signals reported for the pure *trans* product and appeared consistent with previous observations of *cis* 4-substituted cyclohexanamines.

Assignment of Stereochemistry using the ^{1}H NMR signal of ipso hydrogen as observed in the ^{1}H crude reaction: (C_6D_6). Top. Kinetic condition, Bottom. Thermodynamic condition.

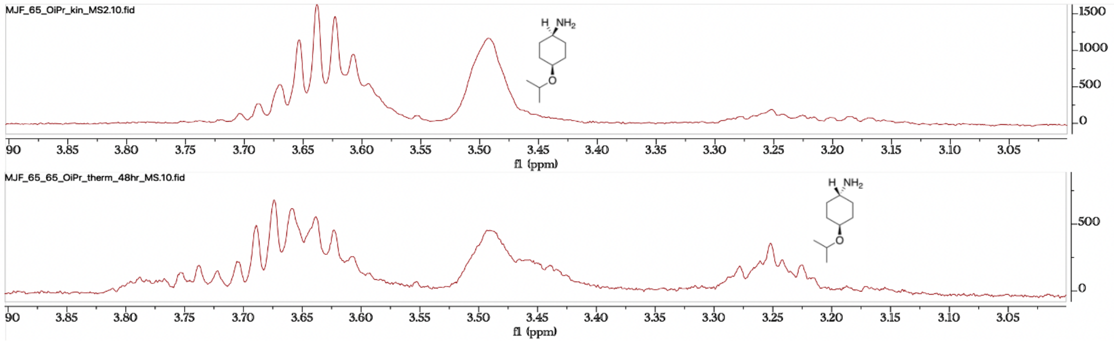

4-Benzyloxycyclohexanone (4A)

GC Data: Top. Kinetic condition, *cis* favoring: 30 eq alanine, WT with pyridoxine hydrochloride, 6 hours. Middle. Thermodynamic condition, *trans* favoring: 2.5 eq alanine, 0.1 eq PLP, WT with pyridoxine hydrochloride. Bottom. Thermodynamic condition, *trans* favoring: 2.5 eq alanine, 0.1 eq PLP, I259V mutant, 6 hours.

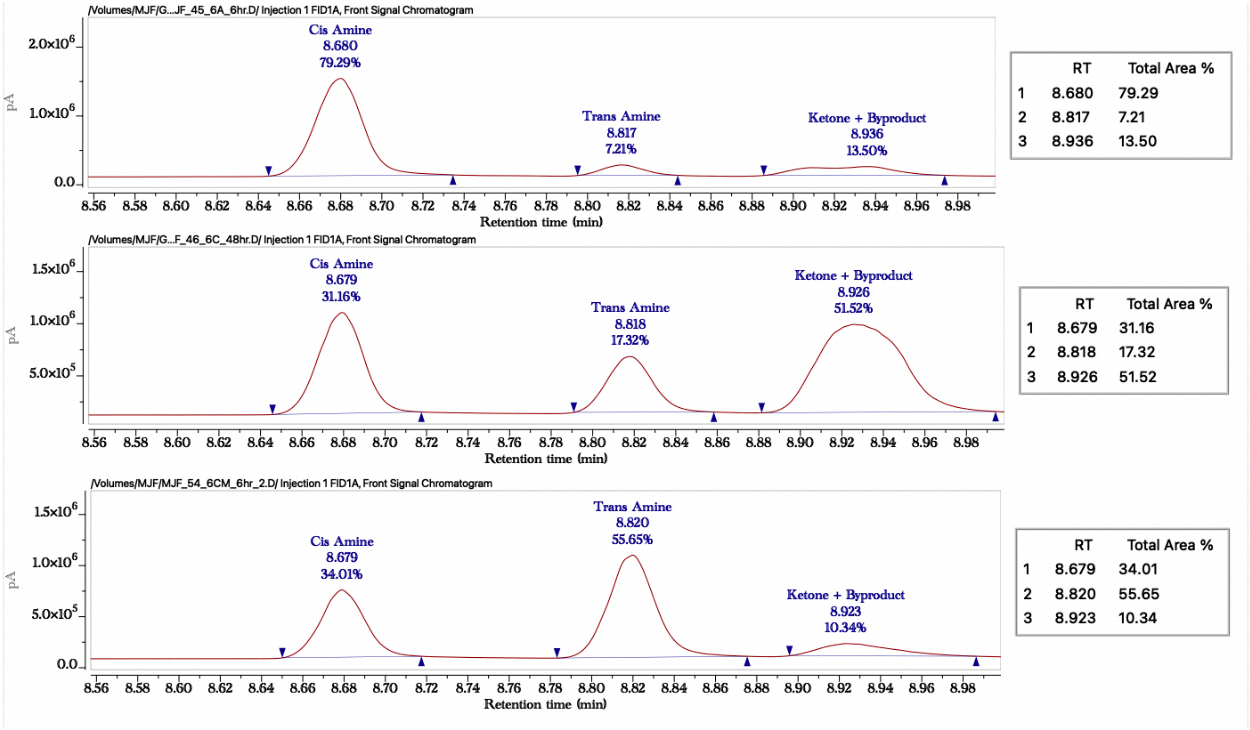

GC-MS Data: Top. Kinetic condition. Bottom. Thermodynamic condition.

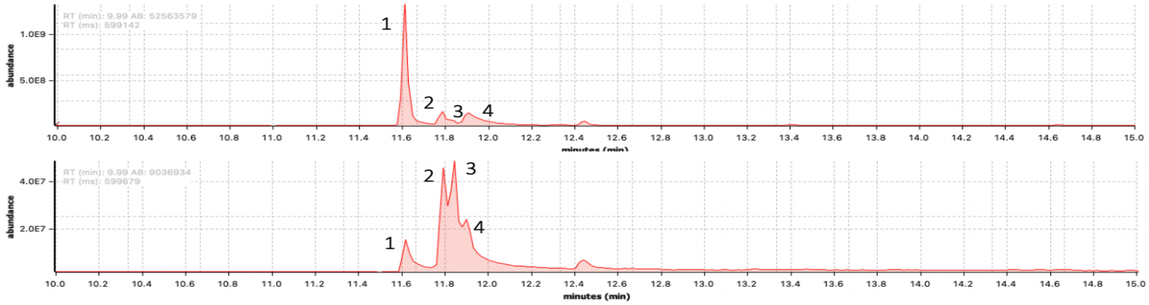

GC-MS Peak 1. *Cis* Amine. MW = 205.

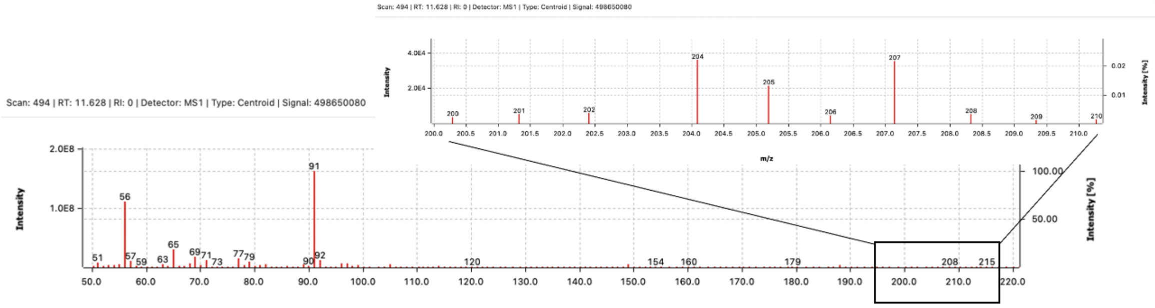

GC-MS Peak 2. *Trans* Amine. MW = 205.

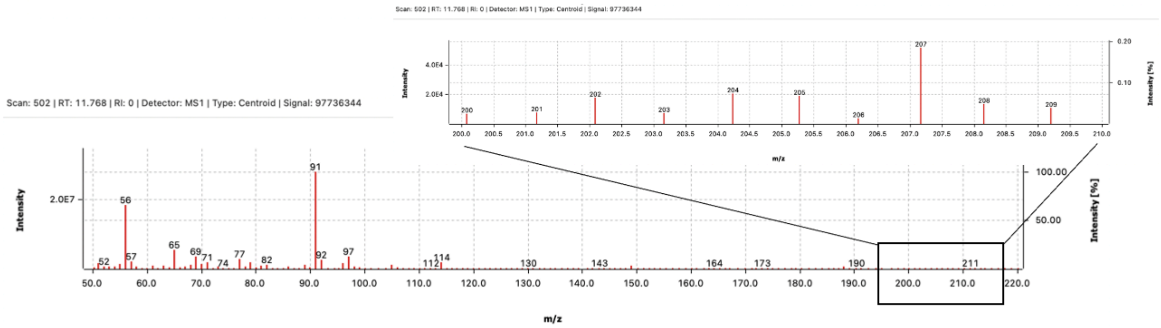

GC-MS Peak 3. Ketone. MW = 204.

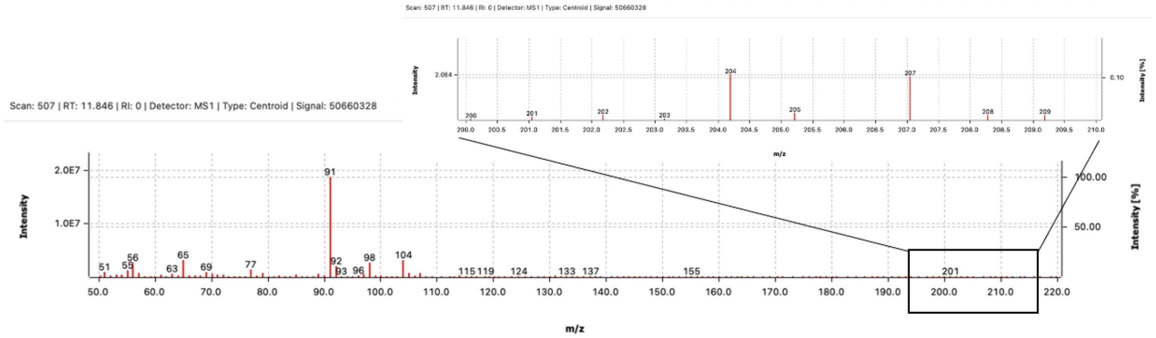

GC-MS Peak 4. Alcohol Byproduct. MW = 206.

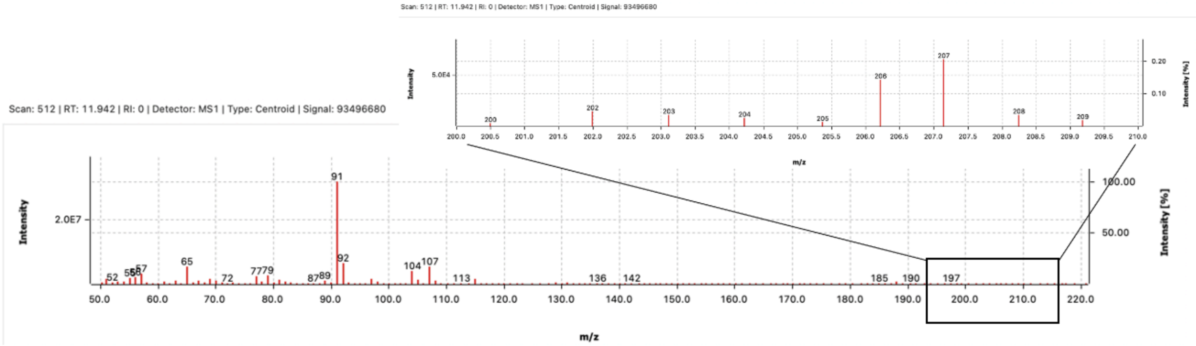

Assignment of Stereochemistry: Crude extracts of the reactions under conditions proposed to be *cis* and *trans* favoring were analyzed by 1H NMR. As observed in other 4-substituted substrates, in the *cis* isomer, the proton ipso to the amine has a higher chemical shift and a smaller coupling constant than the same proton in the *trans* isomer. This same pattern was observed in the case of the products from the benzyl amine. Therefore, by analogy, we assigned the *trans* isomer and *cis* isomer of the 4-benzyloxycyclohexylamine.

Assignment of Stereochemistry using the ^{1}H NMR signal of ipso hydrogen as observed in the ^{1}H crude reaction: (C_6D_6). Top. Kinetic condition, Bottom. Thermodynamic condition.

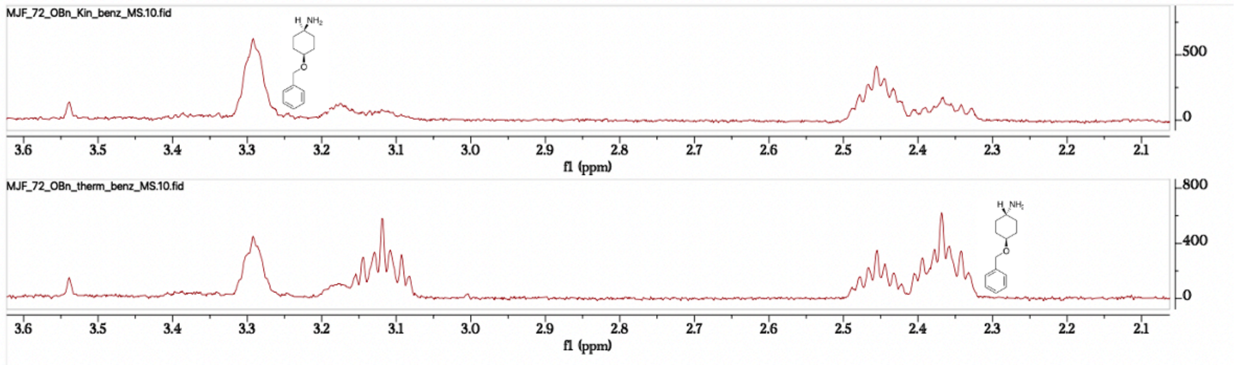

4-tert-Butyl-oxocyclohexanecarboxylate (3A)

GC Data: Top. Kinetic condition, *cis* favoring: 30 eq alanine, WT without pyridoxine hydrochloride, 24 hours. Second. Thermodynamic condition, *trans* favoring: 2.5 eq alanine, 0.1 eq PLP, WT with pyridoxine hydrochloride, 48 hours. Third. Thermodynamic condition, *trans* favoring: 2.5 eq alanine, 0.1 eq PLP, I259V mutant, 48 hours. Bottom: Thermodynamic condition, *trans* favoring: 2.5 eq alanine, 0.1 eq PLP, I259V, W57L mutant, 48 hours.

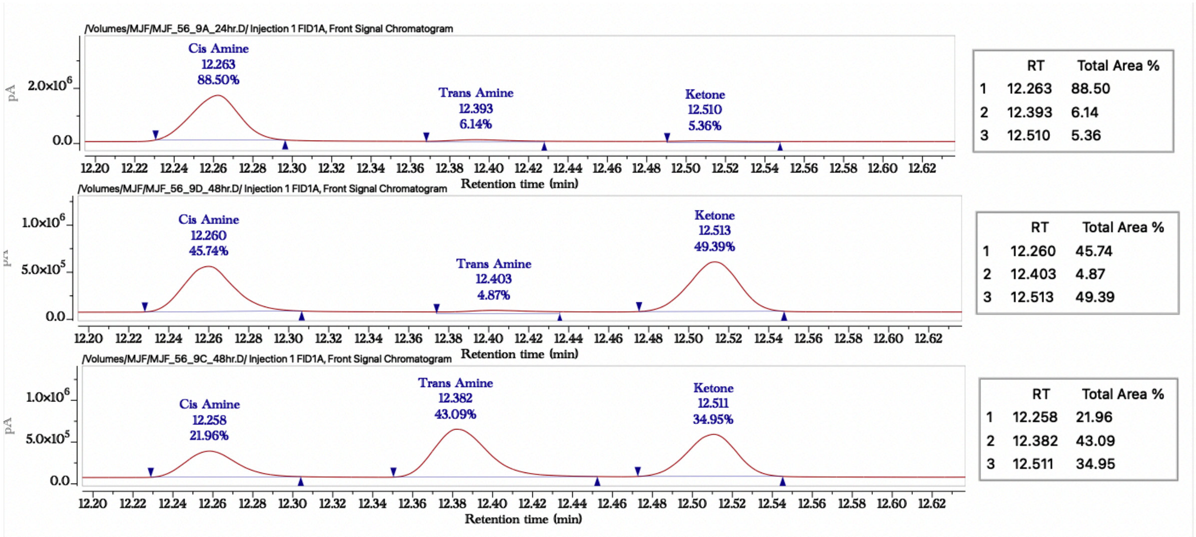

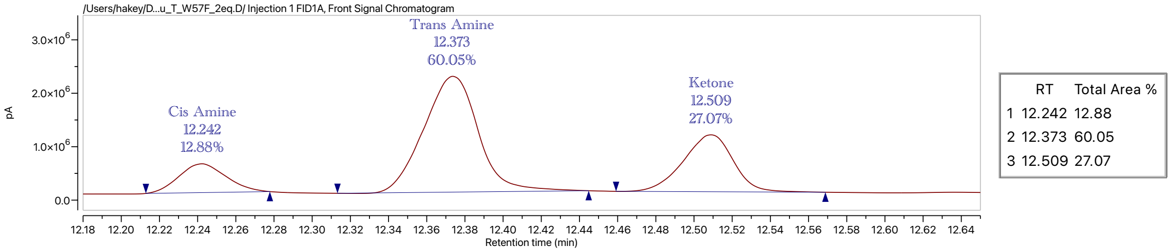

GCMS Data: Top. Kinetic condition. Bottom. Thermodynamic condition.

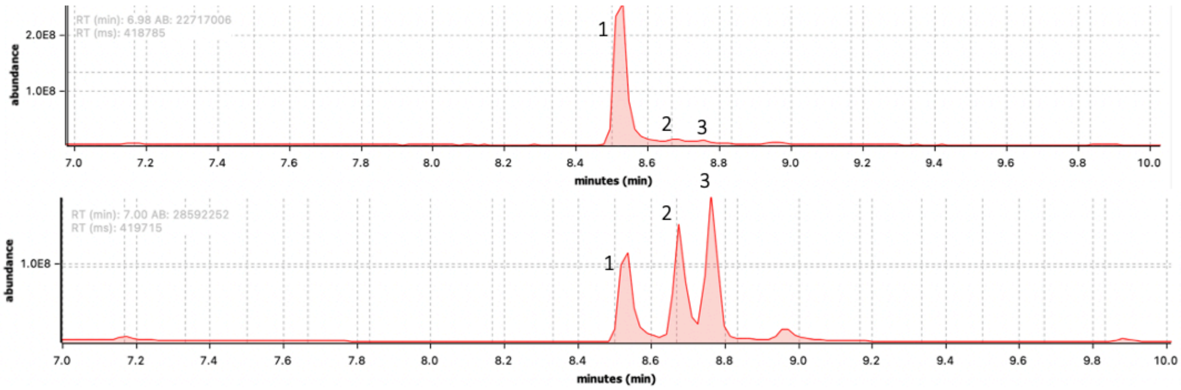

GC-MS Peak 1. *Cis* Amine. MW = 199.

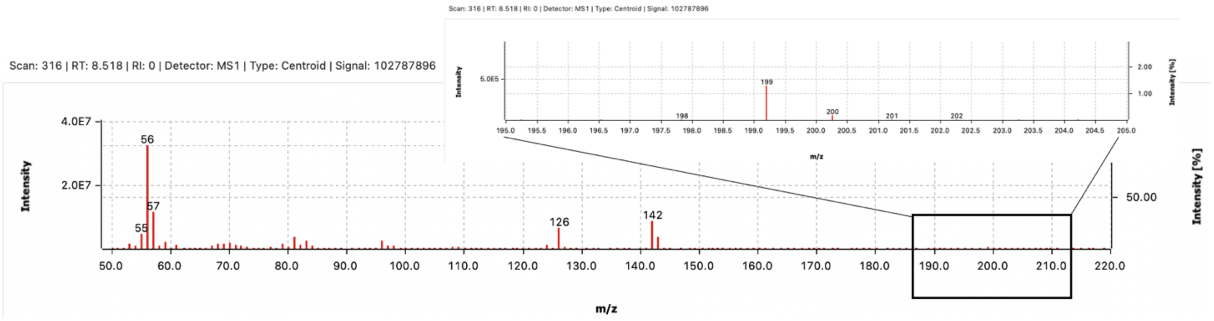

GC-MS Peak 2. *Trans* Amine. MW = 199.

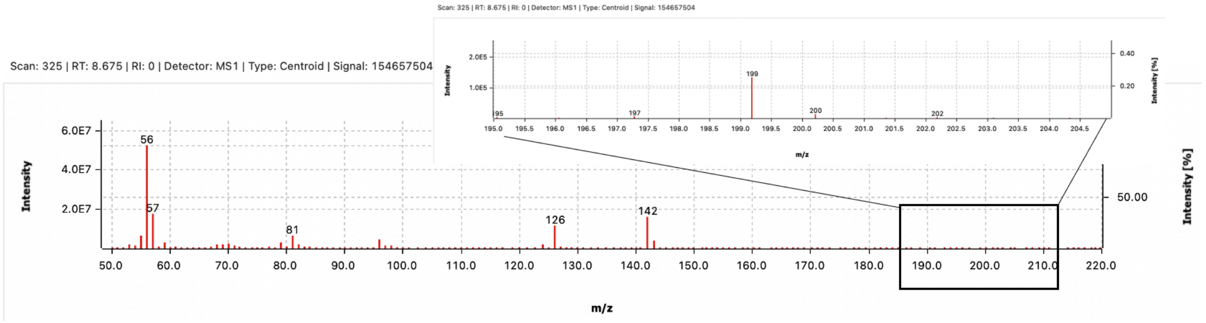

GC-MS Peak 3. Ketone (1i). MW = 198.

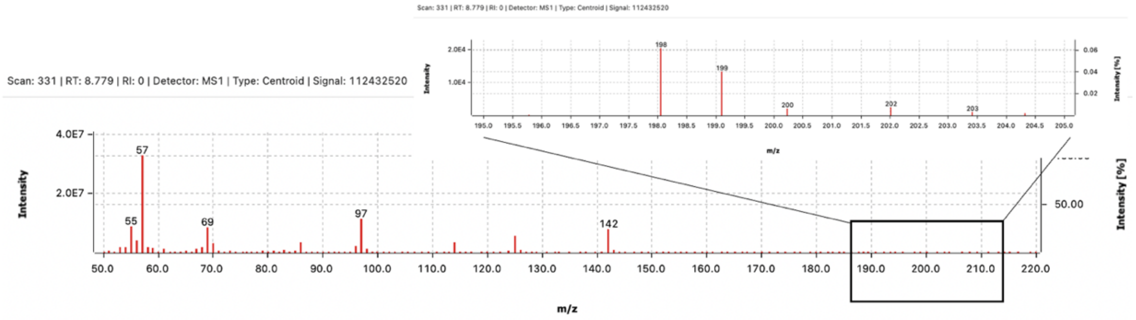

Assignment of Stereochemistry: Crude extracts of the reactions under conditions proposed to be *cis* and *trans* favoring were analyzed by 1H NMR. As observed in other 4-substituted substrates, in the *cis* isomer, the proton ipso to the amine has a higher chemical shift and a smaller coupling constant than the same proton in the *trans* isomer. This same pattern was observed in the case of the products from the tert-butyl ester amine. Therefore we assigned the *trans* isomer and *cis* isomer by analogy

Assignment of Stereochemistry using the ^{1}H NMR signal of ipso hydrogen as observed in the ^{1}H crude reaction: (C_6D_6). Top. Kinetic condition, Bottom. Thermodynamic condition.

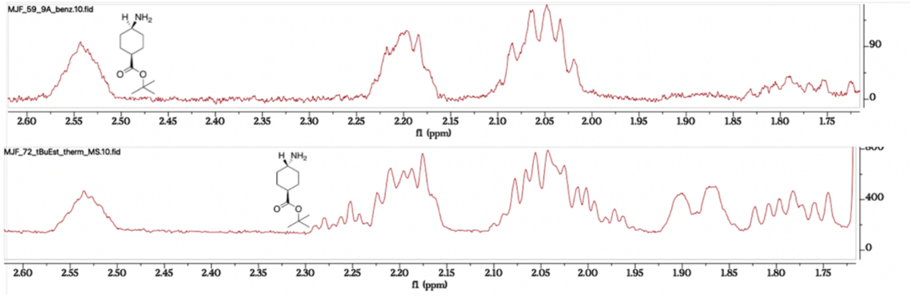

4-tert-butylcyclohexanone (7A)

GC Data: Top: Kinetic Conditions, 30 eq. alanine, 24 hours, WT. Bottom: Thermodynamic Conditions. 2 eq. alanine, 0.1 eq. PLP, 48 hours, WT

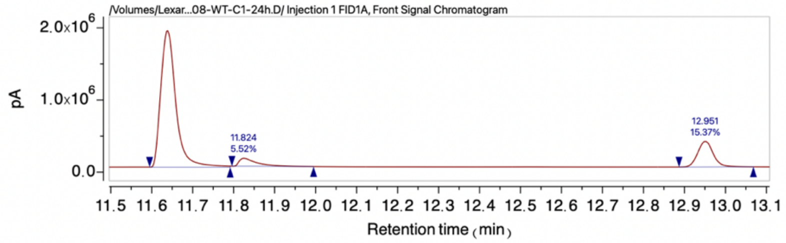

	RT	Total Area %
Cis	11.638	79.11
Trans	11.824	5.52
Ketone	12.951	15.37

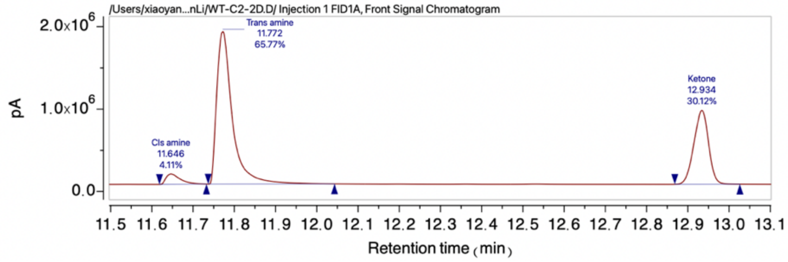

	RT	Total Area %
Cis	11.646	4.11
Trans	11.772	65.77
Ketone	12.934	30.12

GC-MS Data: Top: Kinetic Conditions, Bottom: Thermodynamic Conditions

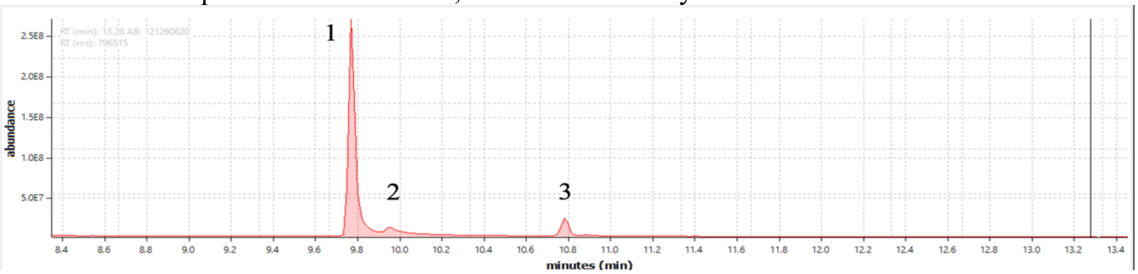

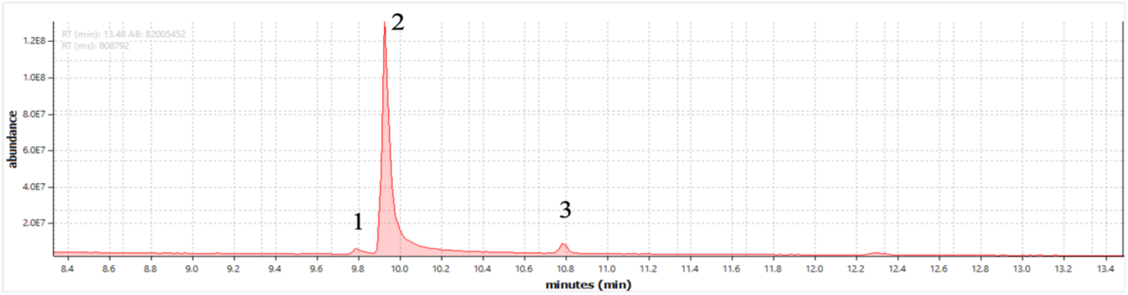

1. Peak 1: *Cis* amine

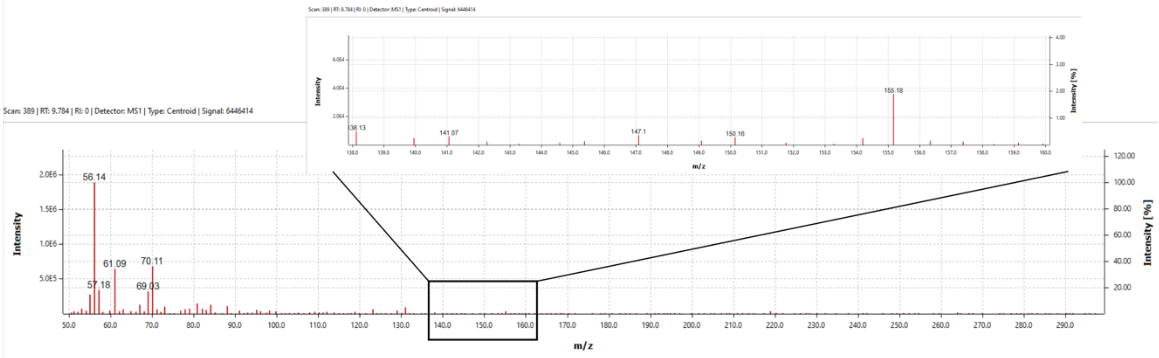

2. Peak 2: *Trans* amine

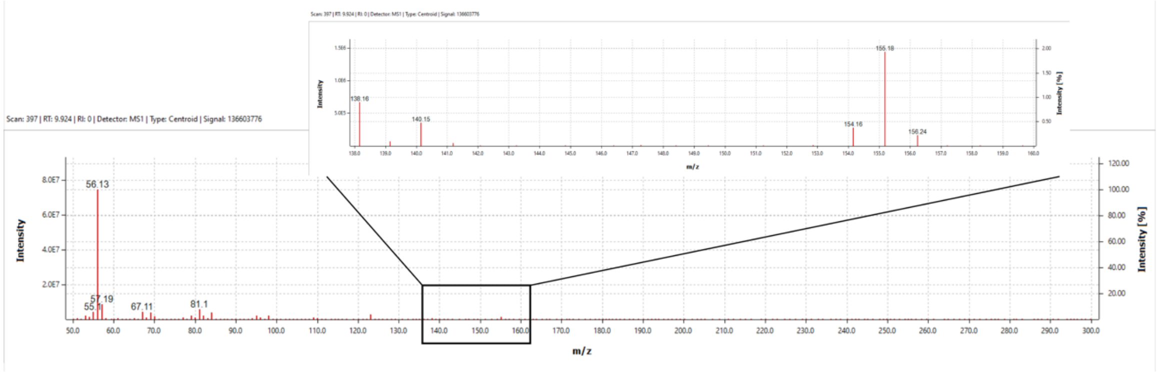

3. Peak 3: Ketone

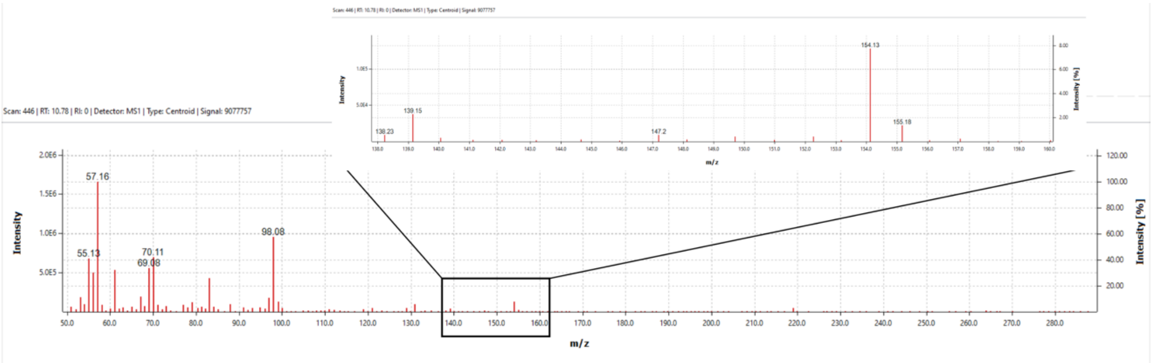

Assignment of Stereochemistry: Crude extracts of the reactions under conditions proposed to be *cis* and *trans* favoring were analyzed by 1H NMR. As reported in the literature, in the *cis* isomer, the proton ipso to the amine has a higher chemical shift and a small coupling constant than the same proton in the *trans* isomer. Thus, comparison to literature data enabled assignment of the *cis* and *trans* isomers.

Assignment of Stereochemistry using the ^{1}H NMR signal of ipso hydrogen as observed in the ^{1}H crude reaction: (C_6D_6). Top. Kinetic condition, Bottom. Thermodynamic condition.

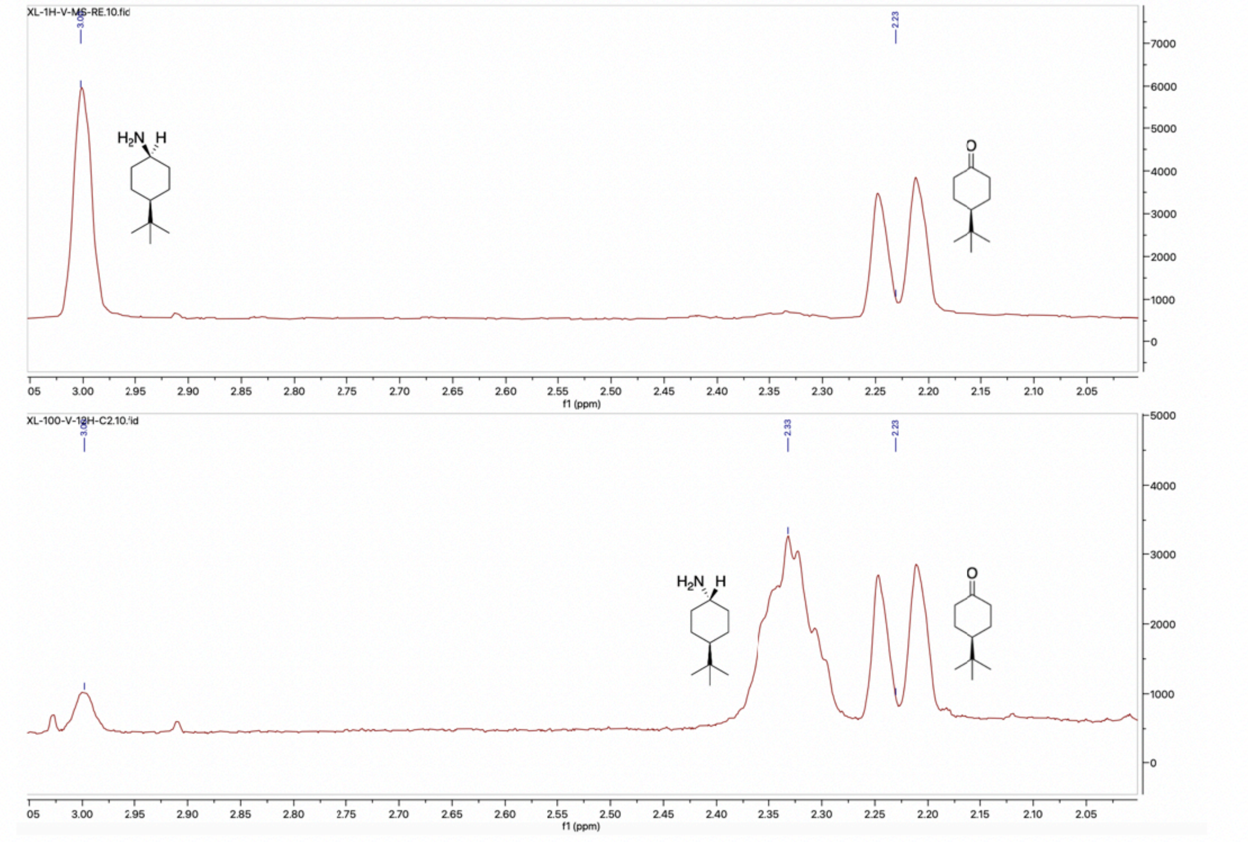

4-Ph Cyclohexanone (8A)

GC Data: Kinetic (30 eq Alanine, WT, 6hrs)

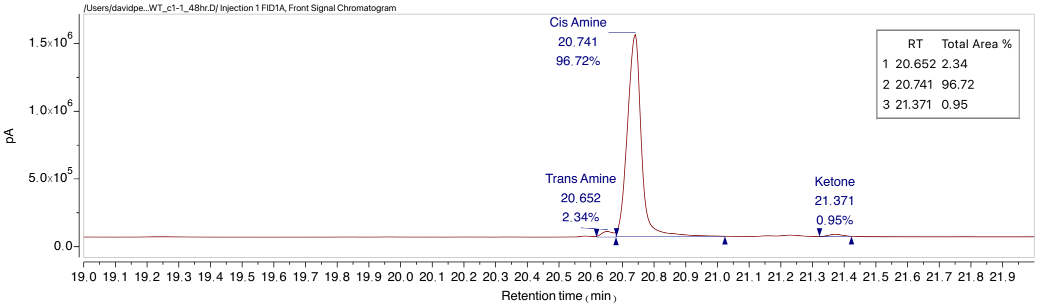

GC Data: Thermodynamic (2.5 eq Alanine, 0.1 eq PLP, WT, 4 days)

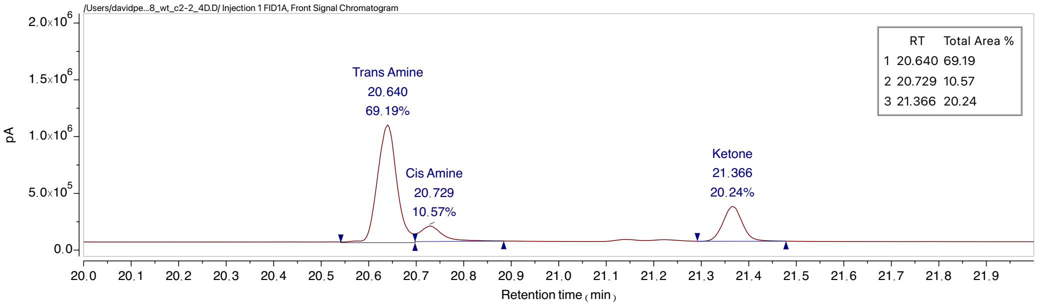

GC Data: Thermodynamic (2.5 Alanine 0.1 PLP, I259V, 4 days)

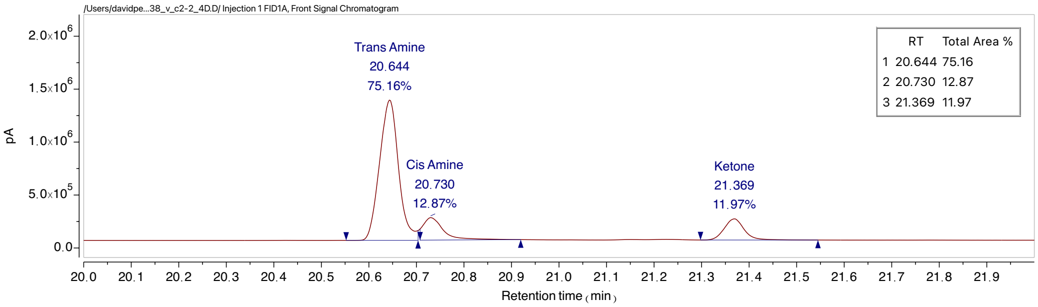

GC-MS Data: Kinetic Conditions

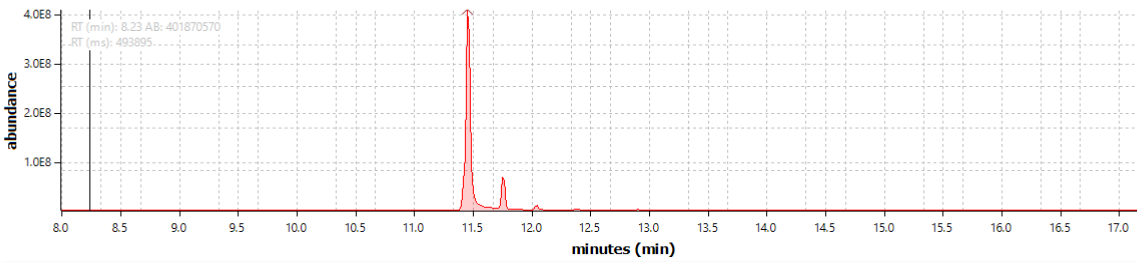

Peak 1 (Amine, overlapped):

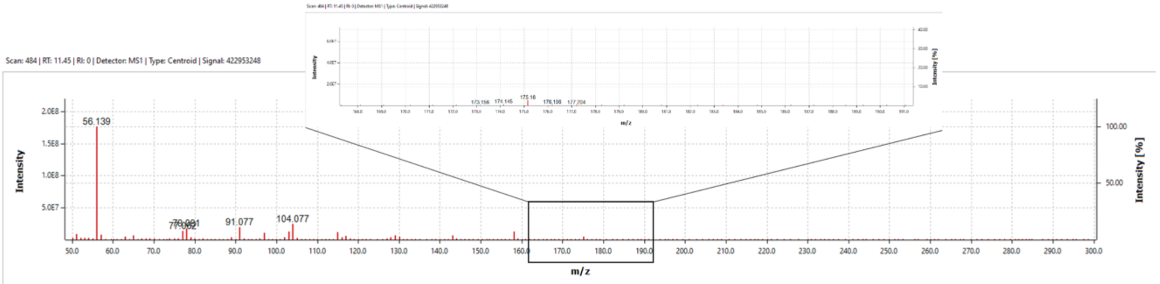

Peak 2 (Ketone):

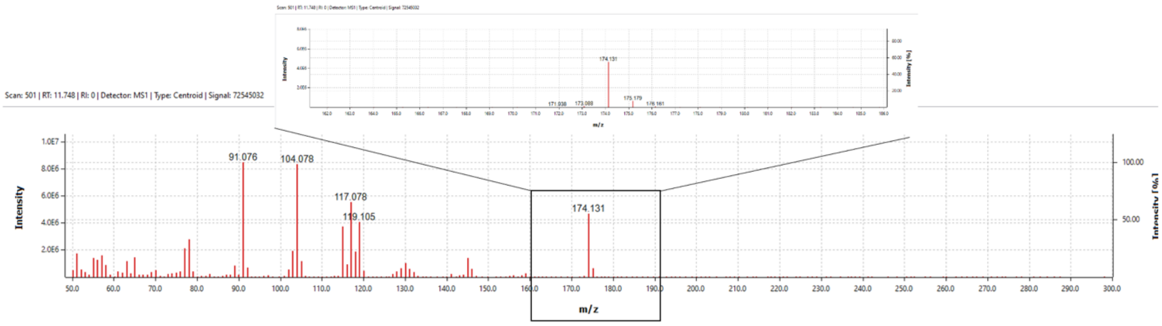

GC-MS Data: Thermodynamic Conditions

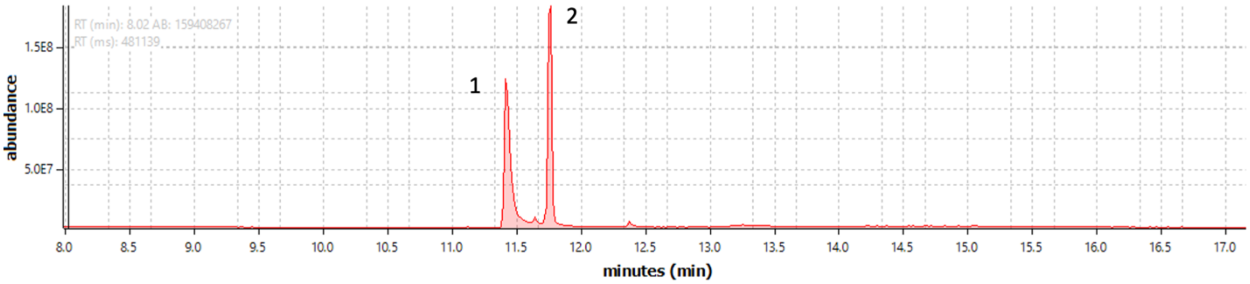

Peak 1: Amine (overlapped)

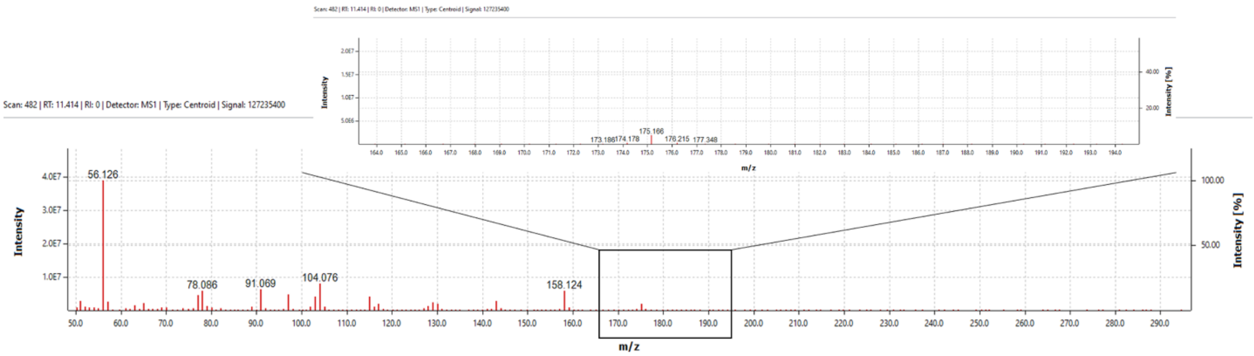

Peak 2: Ketone:

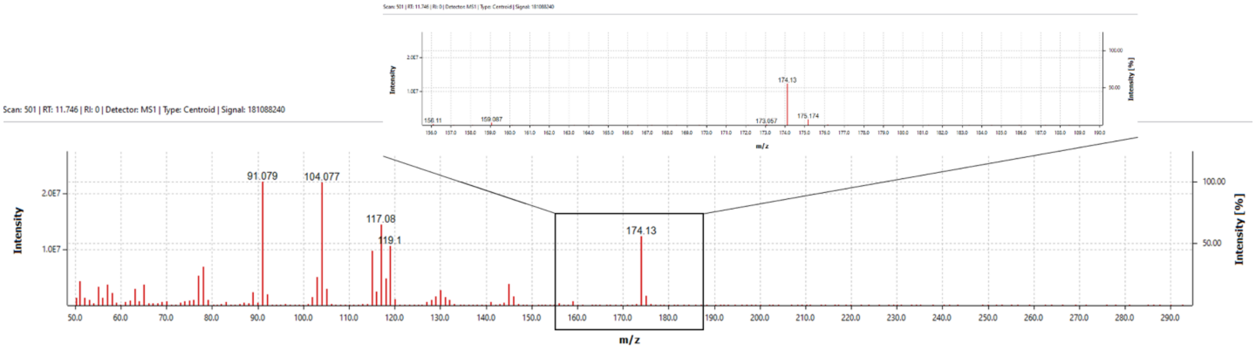

GC of Amide Derivative, Kinetic Conditions

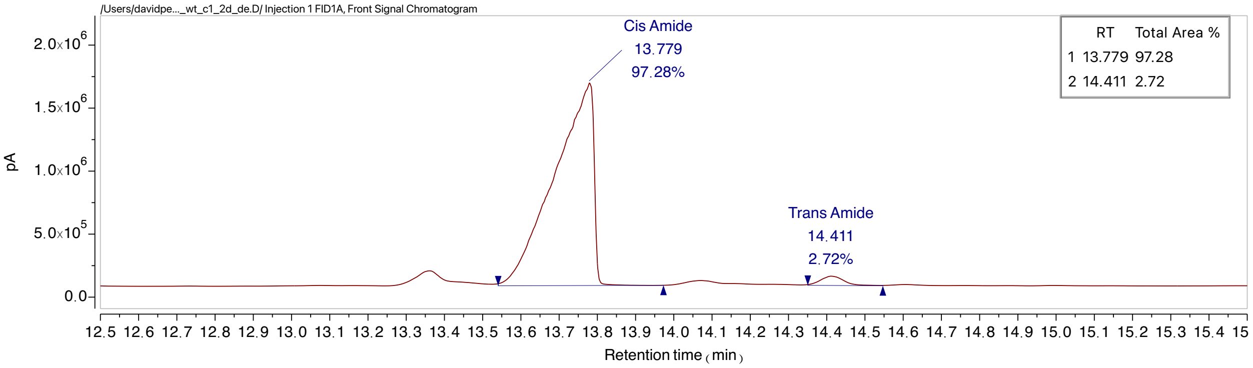

GC of Amide Derivative, Thermodynamic Conditions, WT

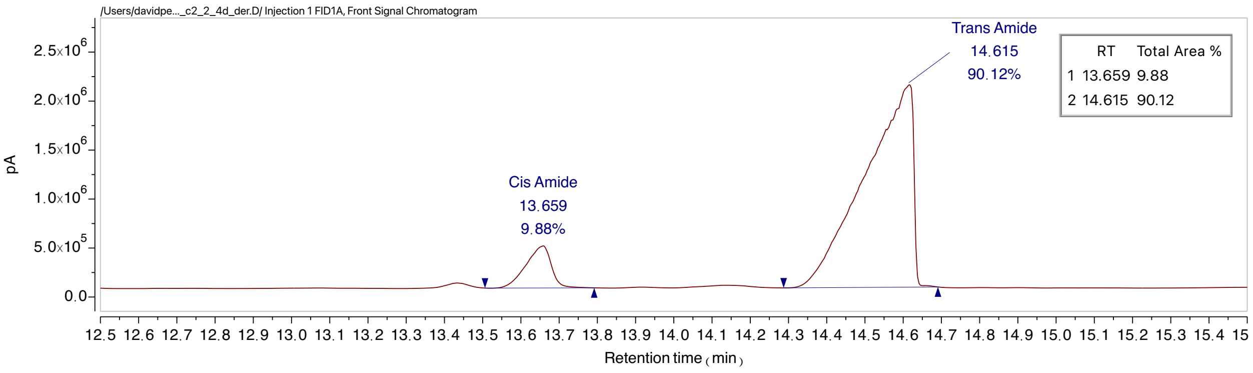

	RT	Total Area %
1	13.659	9.88
2	14.615	90.12

GC of Amide Derivative, Thermodynamic Conditions, I259V

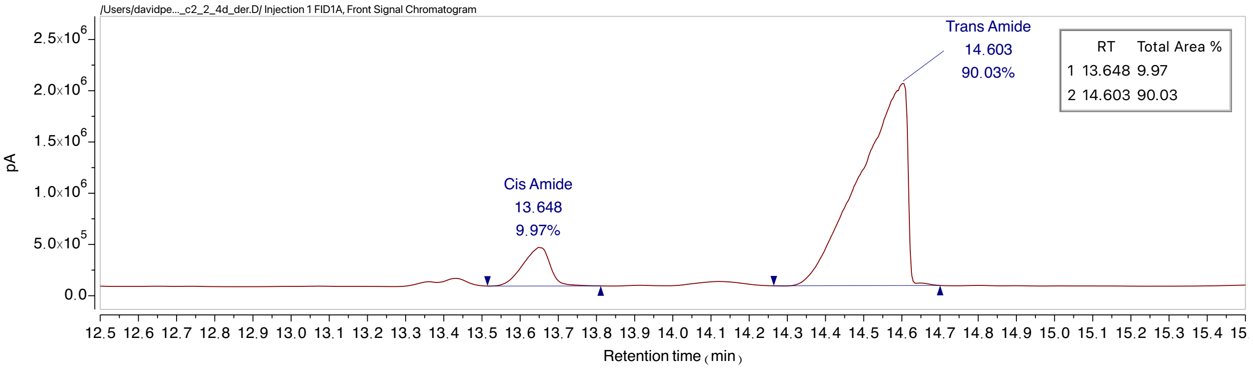

	RT	Total Area %
1	13.648	9.97
2	14.603	90.03

GC-MS of Amide Derivative- Kinetic

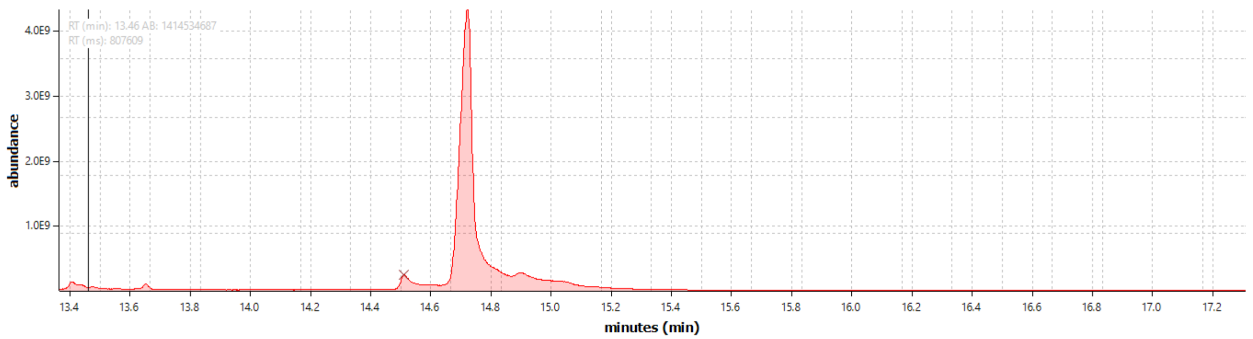

Peak 1, *Cis* Amide:

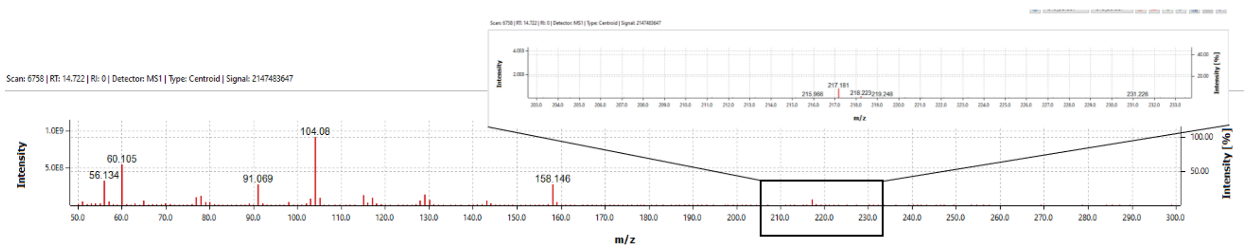

Peak 2, *Trans* Amide:

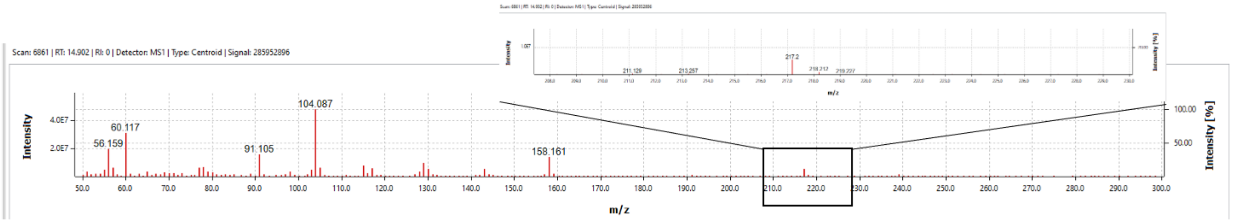

GC-MS of Amide Derivative-Thermodynamic Conditions

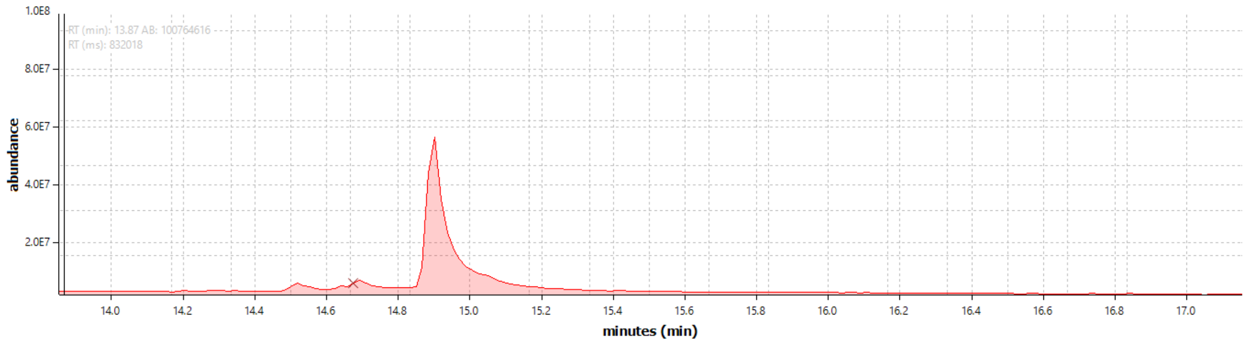

Peak 1- *Cis* Amide

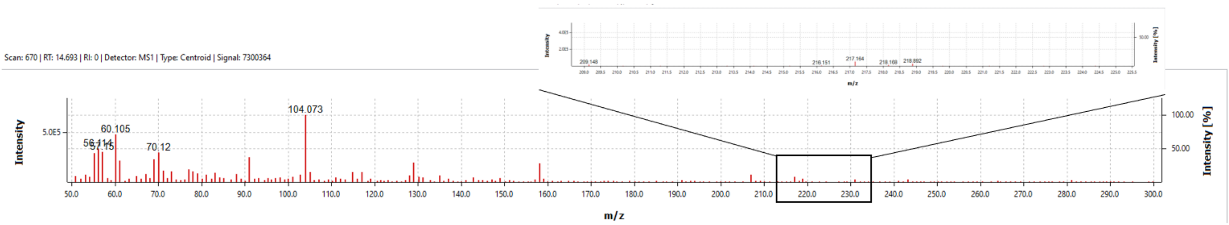

Peak 2- *Trans* Amide

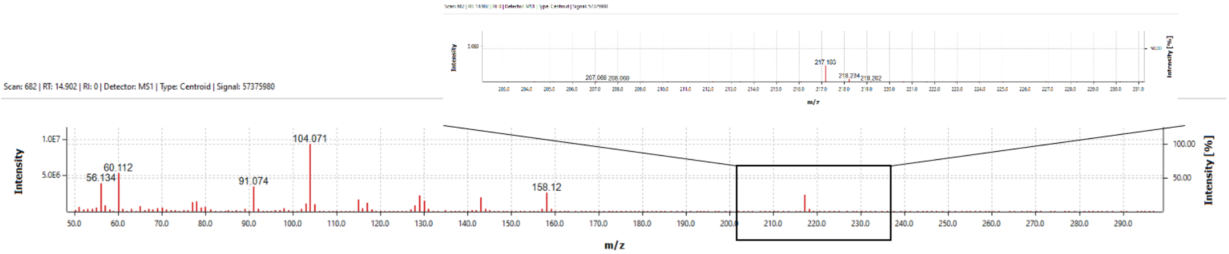

Assignment of Stereochemistry using the ^{1}H NMR signal of ipso hydrogen as observed in the ^{1}H crude reaction: (C_6D_6). Top. Kinetic condition, Bottom. Thermodynamic condition.

NMR (4-Phenyl): Top: Kinetic Conditions. Bottom: Thermodynamic Conditions

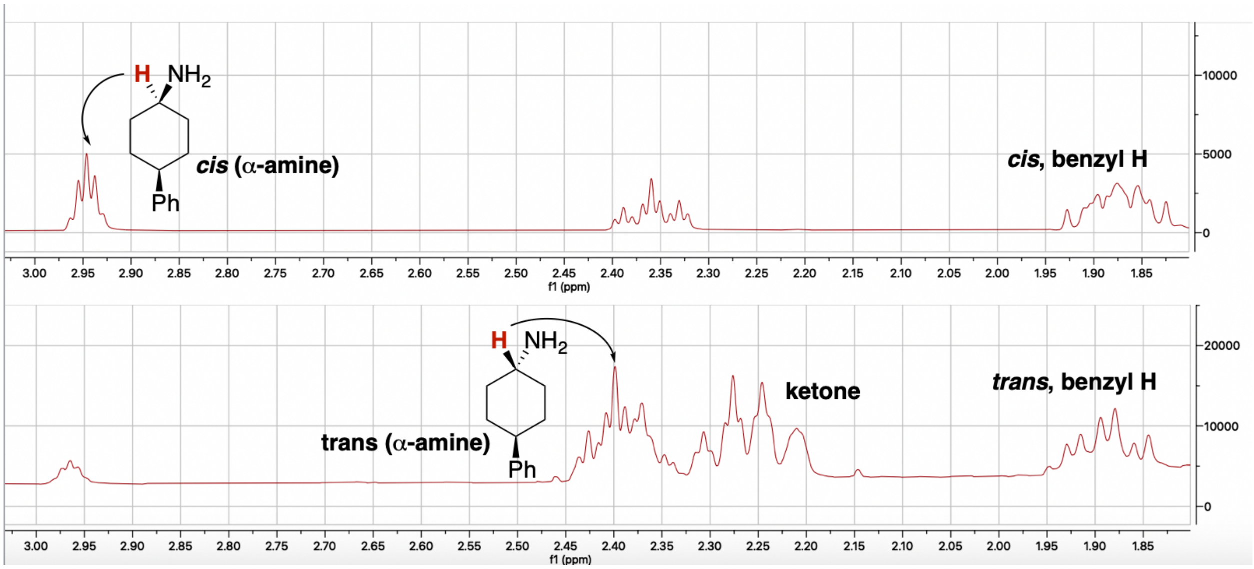

3-Methylcyclohexanone (10A)

GC Data: Top. Kinetic condition, *trans* favoring: 30 eq alanine, WT without pyridoxine hydrochloride, 6 hours. Middle. Thermodynamic condition, *cis* favoring: 2.5 eq alanine, 0.1 eq PLP, WT with pyridoxine hydrochloride, 24 hours. Bottom. Optimized thermodynamic condition, *cis* favoring: 2.5 eq alanine, 0.1 eq PLP, I259V mutant, 4 hours.

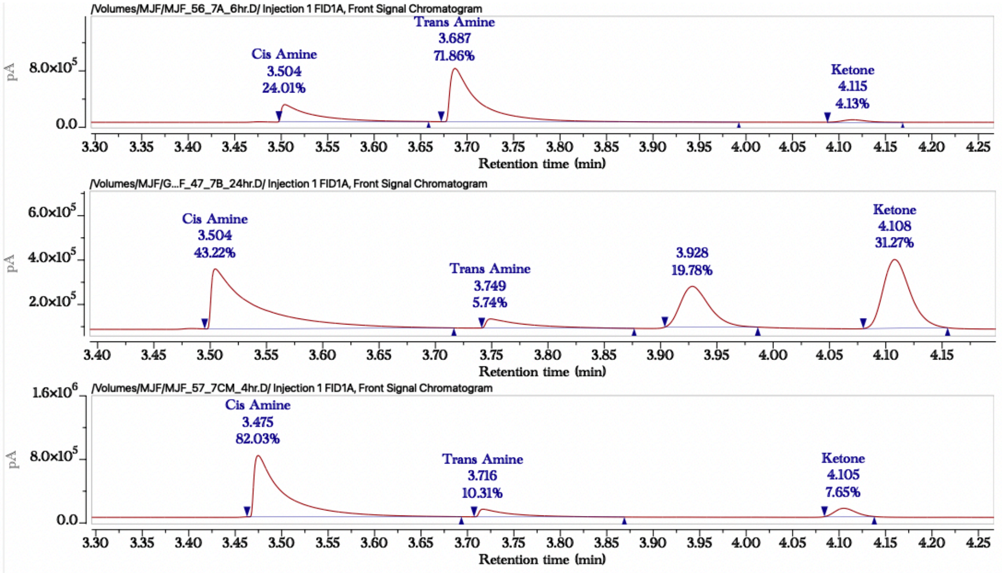

	RT	Total Area %
1	3.504	24.01
2	3.687	71.86
3	4.115	4.13

	RT	Total Area %
1	3.504	43.22
2	3.749	5.74
3	3.928	19.78
4	4.108	31.27

	RT	Total Area %
1	3.475	82.03
2	3.716	10.31
3	4.105	7.65

GC-MS Data: Top. Kinetic condition. Bottom. Thermodynamic condition.

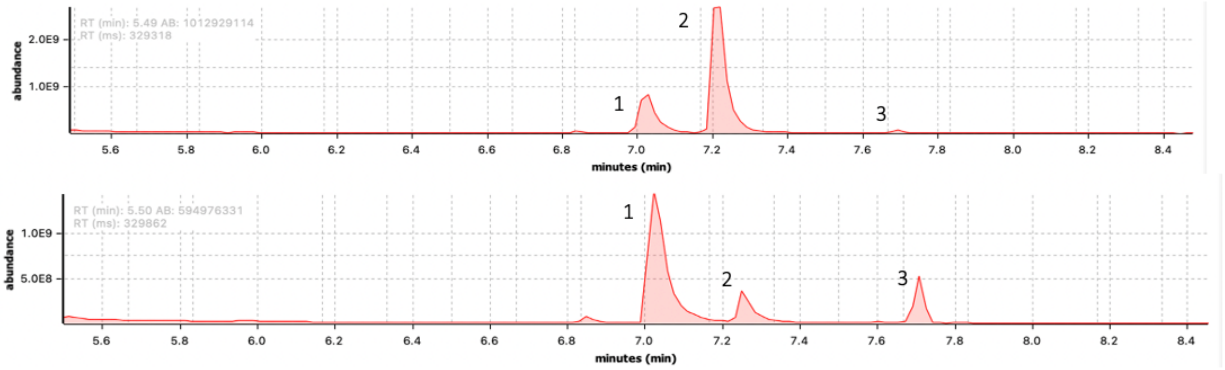

GC-MS Signal 1: *Cis* Amine. MW = 113.

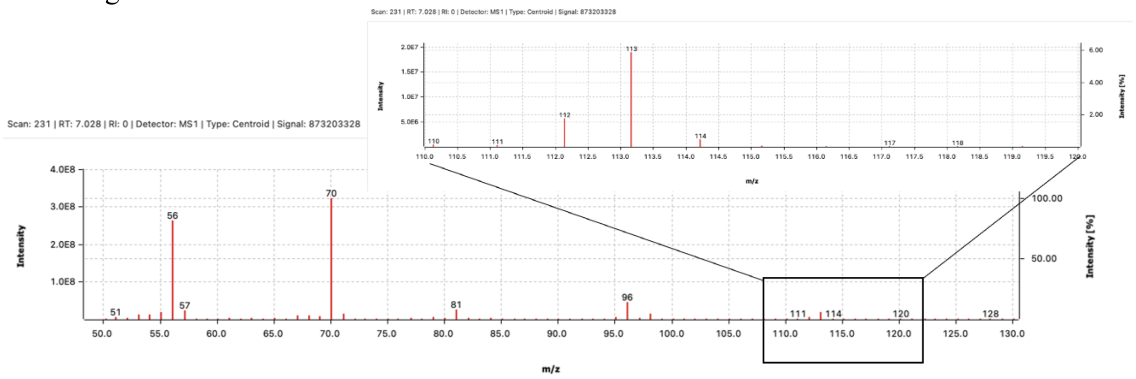

GC-MS Signal 2: *Trans* Amine. MW = 113.

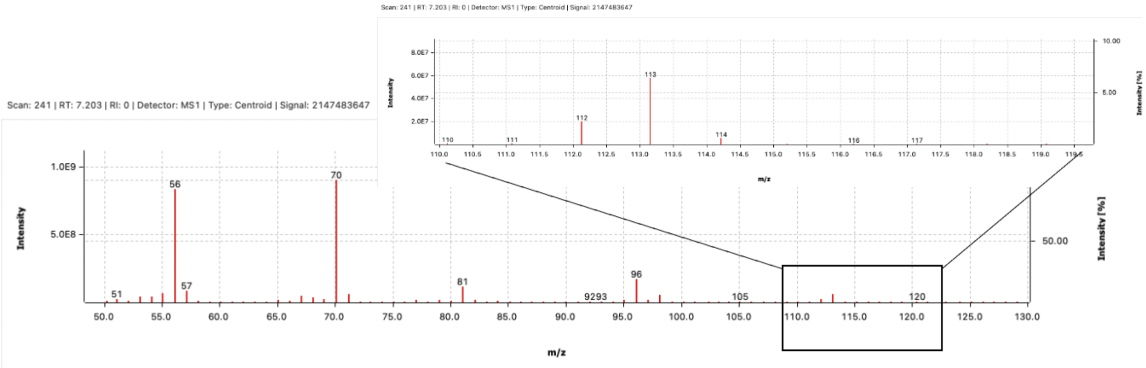

GC-MS Signal 3: Ketone. MW = 112.

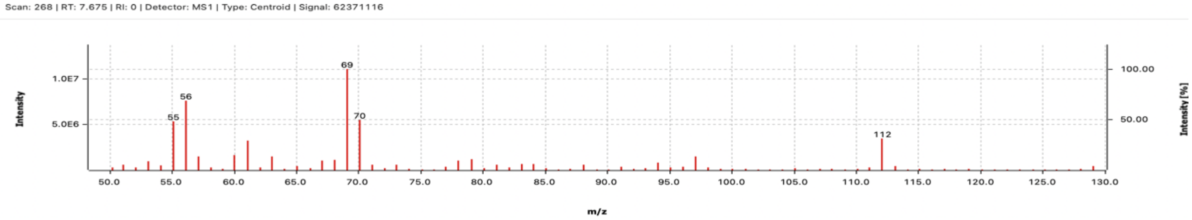

Derivatized Reactions:

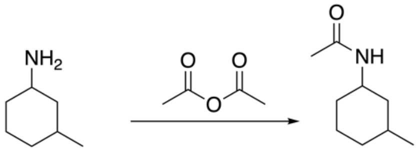

GC Data: Chiral Column. Top: Kinetic condition, *trans* favoring: 30 eq alanine, WT without pyridoxine hydrochloride, 6 hours. Bottom. Thermodynamic condition, *cis* favoring: 2.5 eq alanine, 0.1 eq PLP, I259V mutant, 4 hours.

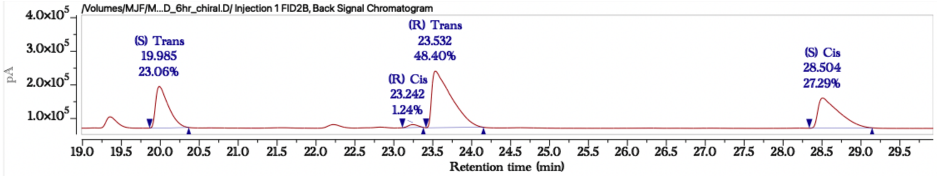

	RT	Total Area %
1	19.985	23.06
2	23.242	1.24
3	23.532	48.40
4	28.504	27.29

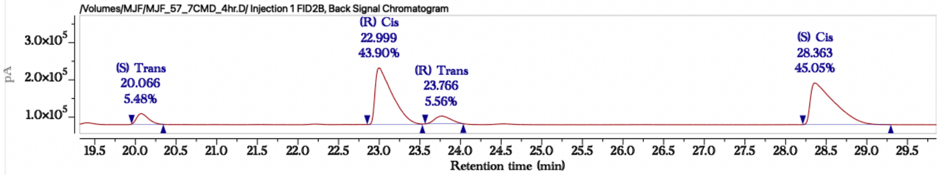

	RT	Total Area %
1	20.066	5.48
2	22.999	43.90
3	23.766	5.56
4	28.363	45.05

GC-MS Data: Top. Kinetic condition. Bottom. Thermodynamic condition.

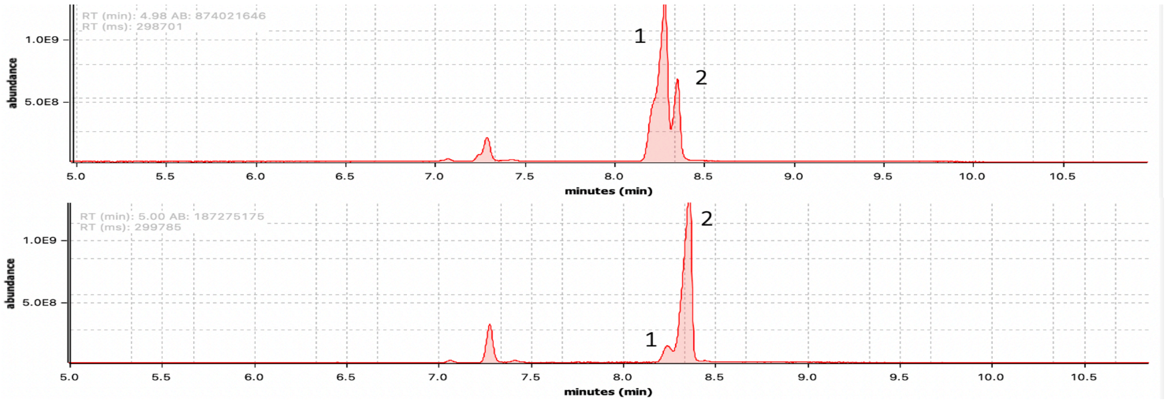

GC-MS Signal 1: *Trans* Amide. MW = 155.

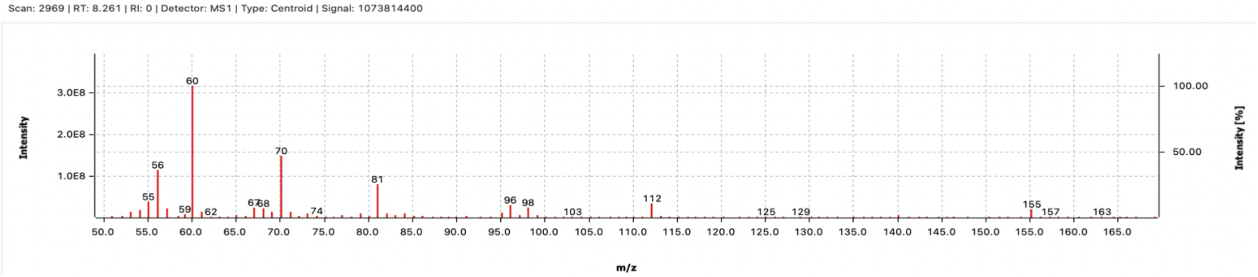

GC-MS Signal 2: *Cis* Amide. MW = 155.

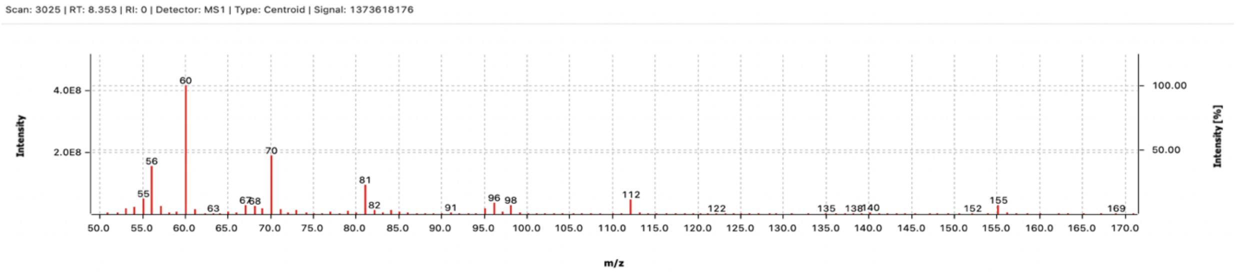

Assignment of Stereochemistry: A commercial solution of 3-Me cyclohexylamine (mixture of diastereomers) was purchased and analyzed by 1H NMR. Comparison to literature data showed the mixture was ~3:1 *trans* to *cis*. The sample was then analyzed by GC, producing two signals in a similar ratio, enabling assignment of the retention times of the cis and *trans* amines. The commercial amine was then derivatized with acetic anhydride to form a ~3:1 mixture of the corresponding amides. The derivatized product was analyzed by chiral GC to determine the retention times of the resulting *cis* and *trans* amides with concurrent separation of the four enantiomers. Separately, we purchased the (*R*)-3-methyl ketone, which, under varied reaction conditions, formed either *cis* (*S*,*R*) or *trans* (*R*,*R*) favoring mixtures of products. Analysis of this mixture of products by chiral GC identified the member of each *cis* and *trans* pair with (*R*)-stereochemistry at the methyl group. Thus, the signals from the stereoisomers with (*S*) stereochemistry at the methyl group were determined to be the other two signals. Thus, all four stereoisomers could be separated, assigned, and quantified.

Assignment of Stereochemistry using the ^{1}H NMR signal of ipso hydrogen as observed in the ^{1}H crude reaction: (C_6D_6). Top. 3-Me commercial mixture of diastereomers. Middle. Kinetic condition. Bottom. Thermodynamic condition.

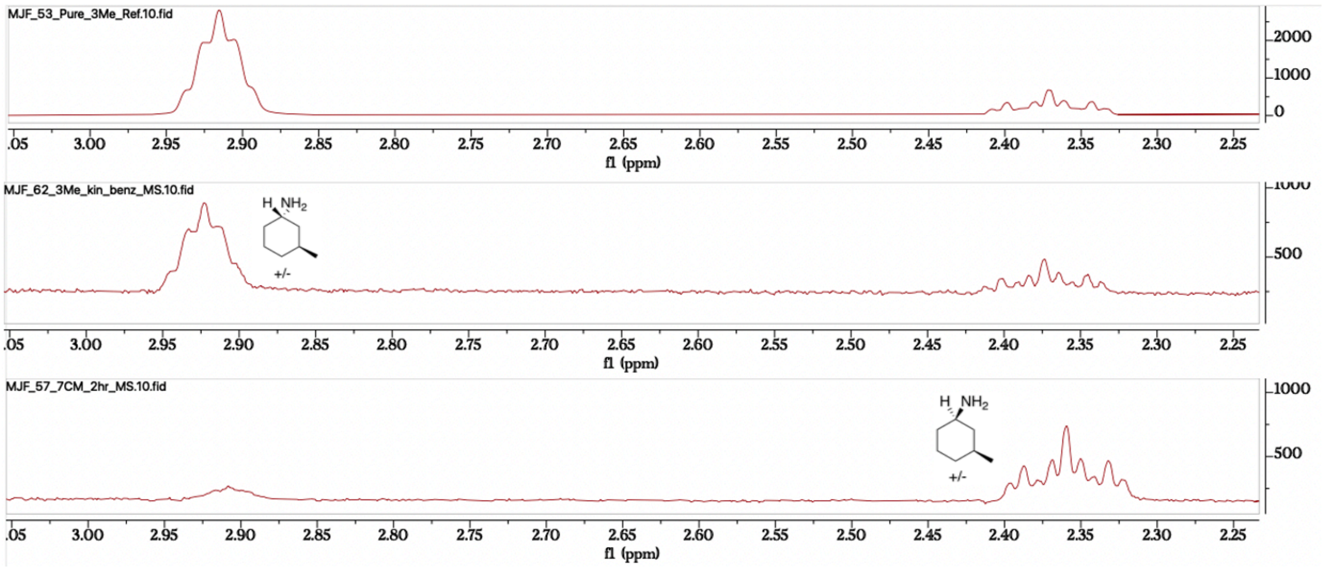

(*S*) 3-Methylcyclohexanone (analyzed from reaction of racemate)- (*S*)-11A

Derivatized Reactions

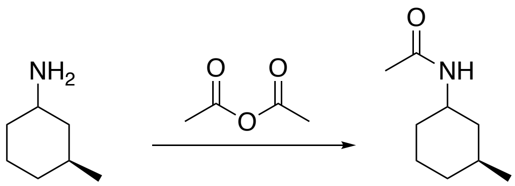

GC Data: Kinetic condition, *trans* favoring: 30 eq alanine, WT without pyridoxine hydrochloride, 6 hours. Top. Racemic 3-Me. Bottom. (*R*)-3-Me.

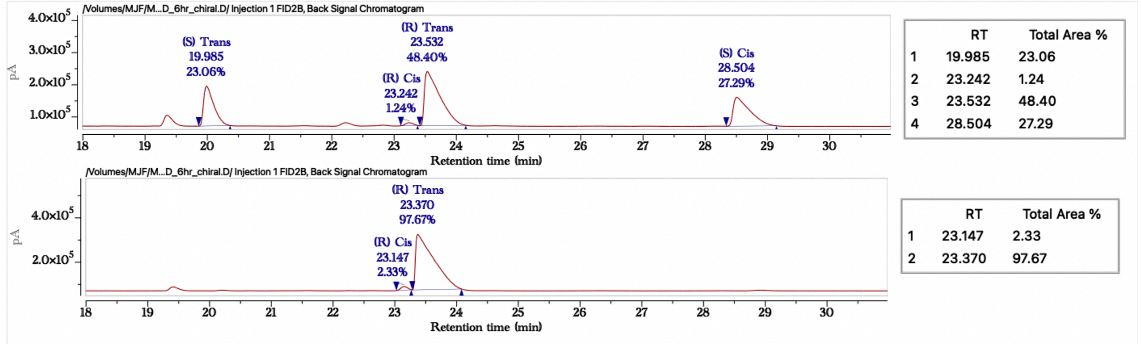

	RT	Total Area %
1	19.985	23.06
2	23.242	1.24
3	23.532	48.40
4	28.504	27.29

	RT	Total Area %
1	23.147	2.33
2	23.370	97.67

Thermodynamic condition, *cis* favoring: 2.5 eq alanine, 0.1 eq PLP, WT with pyridoxine hydrochloride, 24 hours. Top. Racemic 3-Me. Bottom. (*R*)-3-Me.

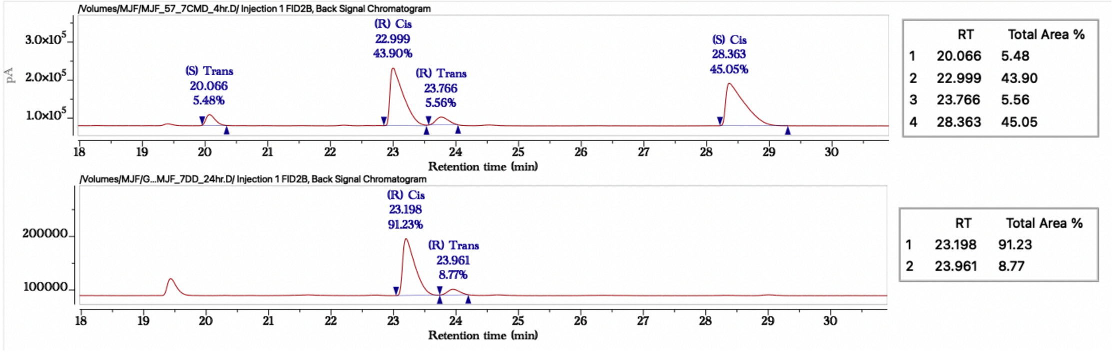

	RT	Total Area %
1	20.066	5.48
2	22.999	43.90
3	23.766	5.56
4	28.363	45.05

	RT	Total Area %
1	23.198	91.23
2	23.961	8.77

Assignment of Stereochemistry: *Cis* and *trans* products were assigned to be consistent with those assigned for the racemic and (*R*) 3-Me data.

(*R*) 3-Methylcyclohexanone (analyzed as reaction of pure enantiomer) – (*R*)-10A

GC Data: Top. Kinetic condition, *trans* favoring: 30 eq alanine, WT without pyridoxine hydrochloride, 6 hours. Bottom. Thermodynamic condition, *cis* favoring: 2.5 eq alanine, 0.1 eq PLP, WT with pyridoxine hydrochloride, 24 hours.

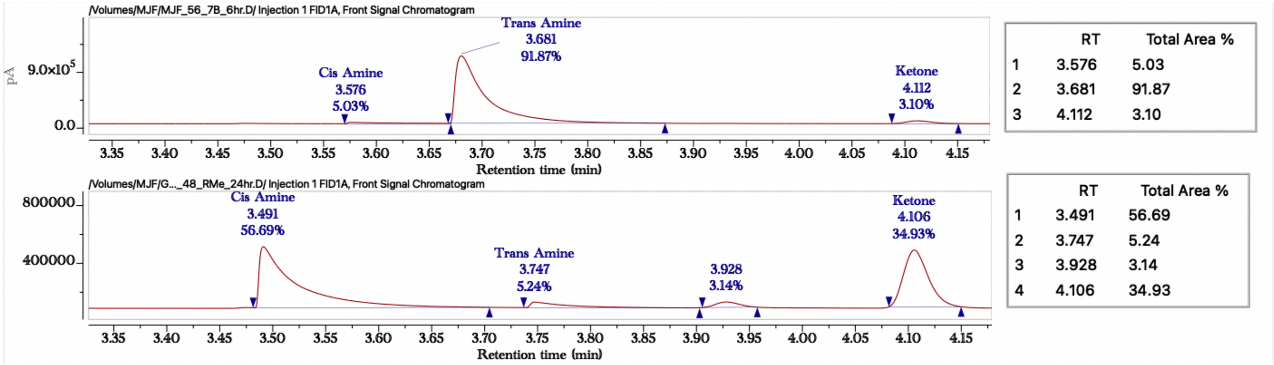

Derivatized Reactions:

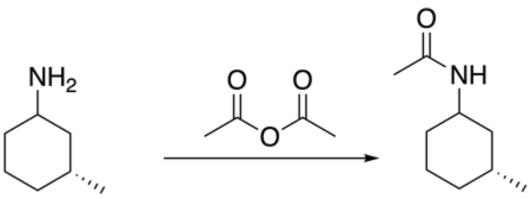

GC Data: Top. Kinetic condition, *trans* favoring: 30 eq alanine, WT without pyridoxine hydrochloride, 6 hours. Bottom. Thermodynamic condition, *cis* favoring: 2.5 eq alanine, 0.1 eq PLP, WT with pyridoxine hydrochloride, 24 hours.

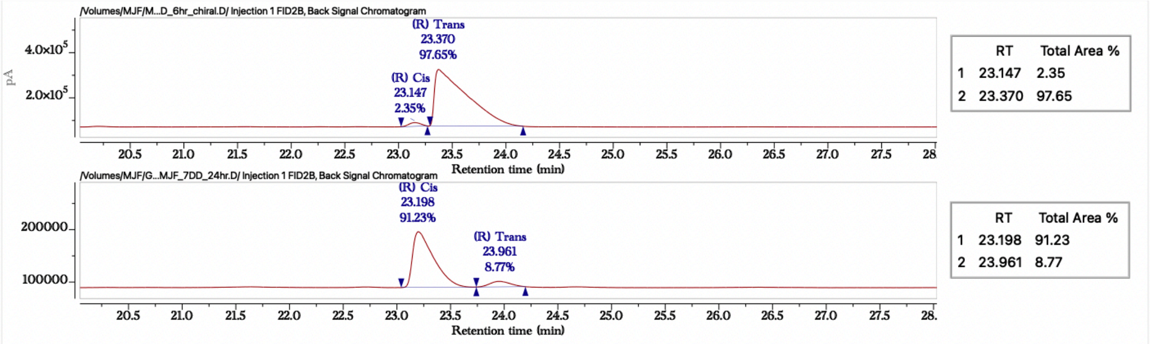

Assignment of Stereochemistry: *Cis* and *trans* products were assigned to be consistent with those assigned for the racemic 3-Me data.

3-Trifluoromethylcyclohexanone – 12A (analyzed as racemate)

GC Data (amines do not separate fully in GC): Top. Kinetic condition, *trans* favoring: 30 eq alanine, WT without pyridoxine hydrochloride, 6 hours. Bottom. Thermodynamic condition, *cis* favoring: 2.5 eq alanine, 0.1 eq PLP, WT with pyridoxine hydrochloride, 6 hours.

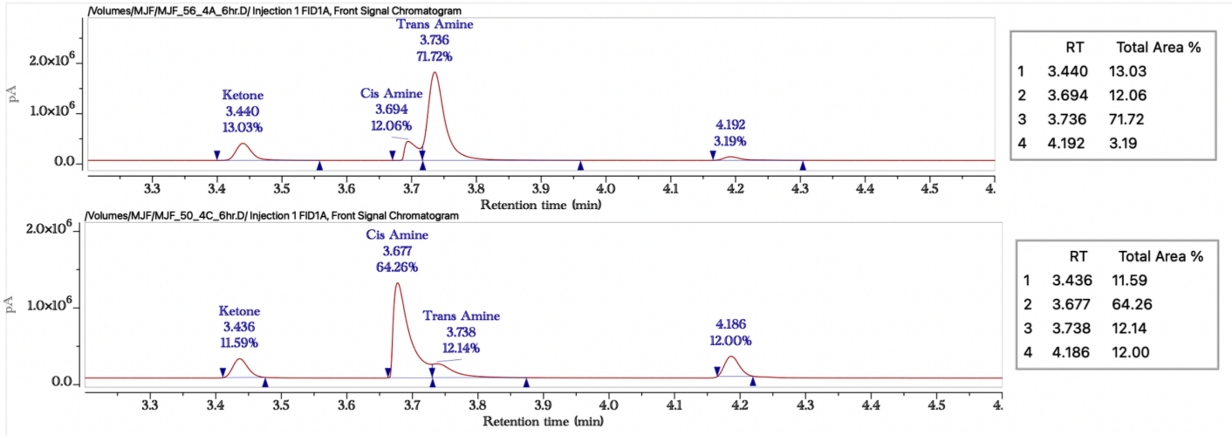

GC-MS Data: Top. Kinetic condition. Bottom. Thermodynamic condition.

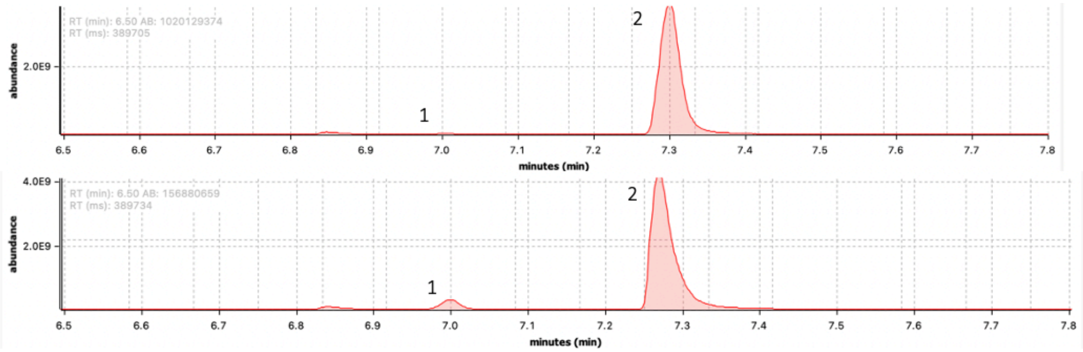

GC-MS Signal 1: Ketone. MW = 166.

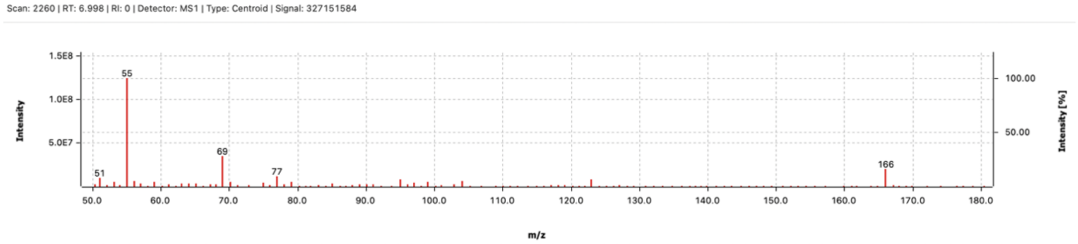

GC-MS Signal 2: Overlapped Amine Products. MW = 167.

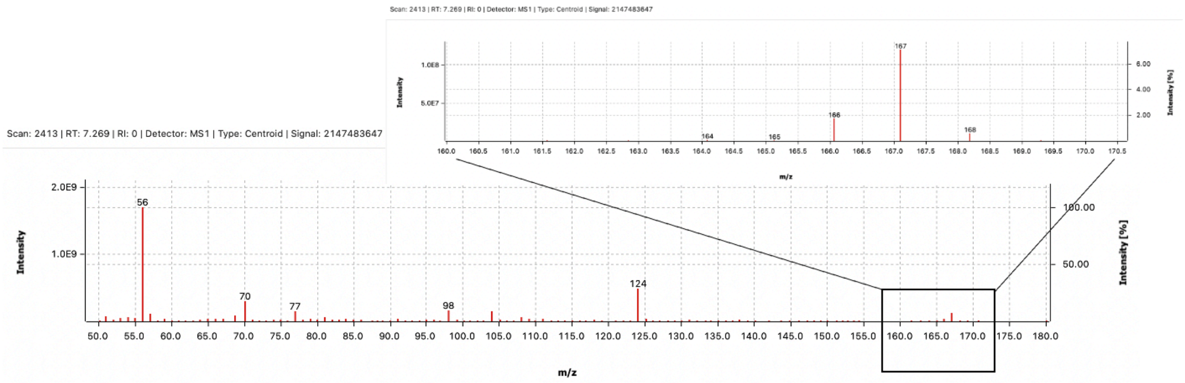

Derivatized Reactions:

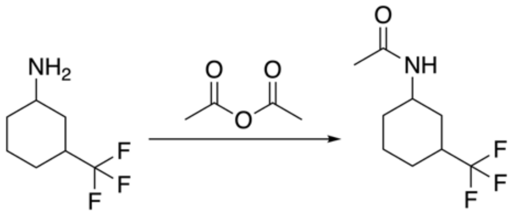

GC Data/Achiral Column: Top. Kinetic condition, *trans* favoring: 30 eq alanine, WT without pyridoxine hydrochloride, 6 hours. Bottom. Thermodynamic condition, *cis* favoring: 2.5 eq alanine, 0.1 eq PLP, WT with pyridoxine hydrochloride, 6 hours.

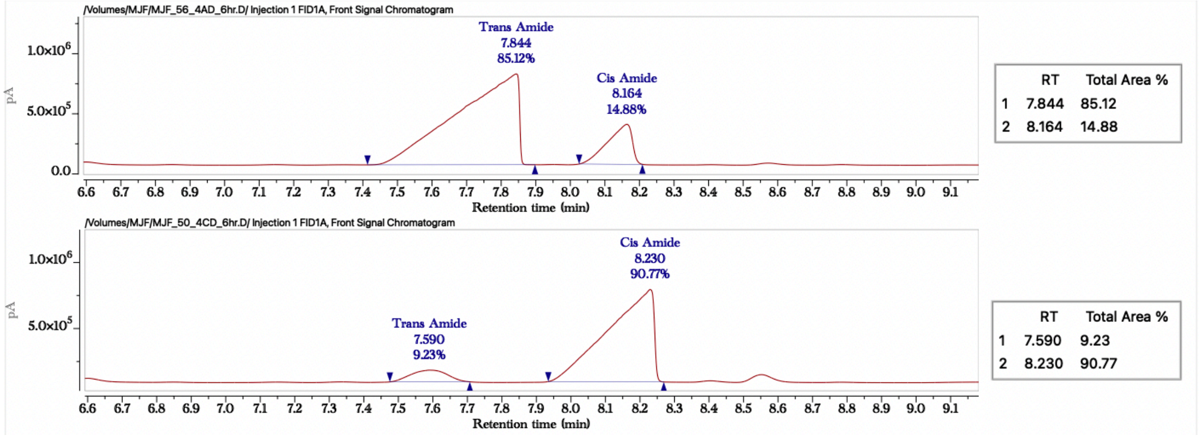

GC Data/Chiral column. Top. Kinetic condition, *trans* favoring: 30 eq alanine, WT without pyridoxine hydrochloride, 6 hours. Bottom. Thermodynamic condition, *cis* favoring: 2.5 eq alanine, 0.1 eq PLP, WT with pyridoxine hydrochloride, 6 hours.

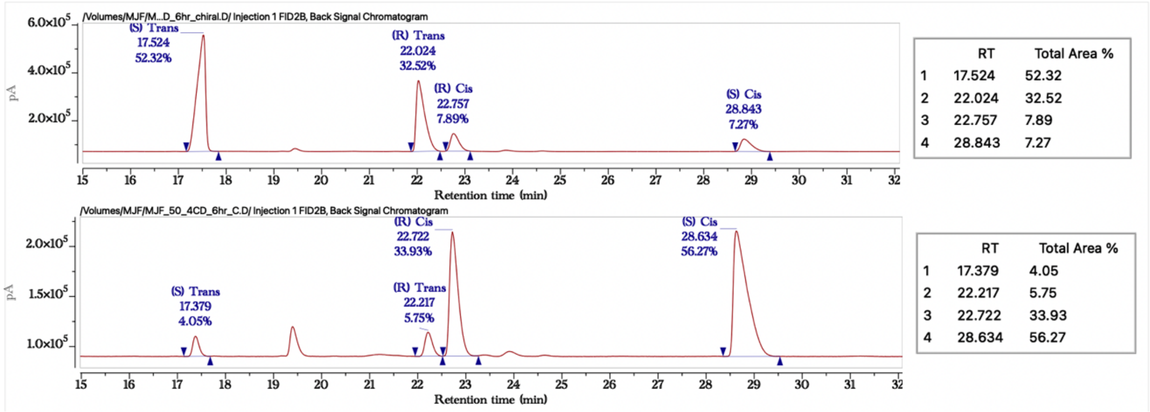

GC-MS Data: Top. Kinetic condition. Bottom. Thermodynamic condition.

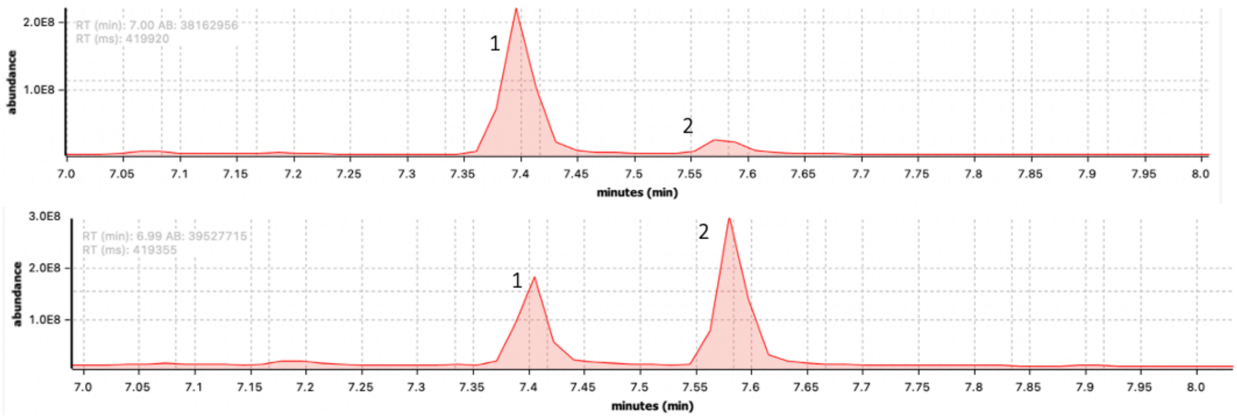

GC-MS Signal 1: *Trans* Amide. MW = 209.

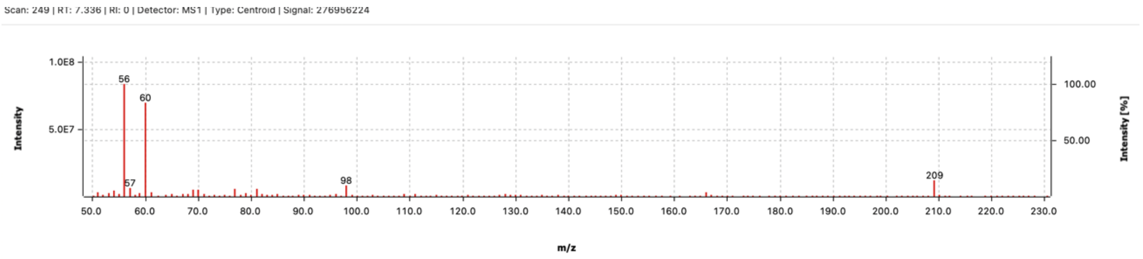

GC-MS Signal 2: *Cis* Amide. MW = 209.

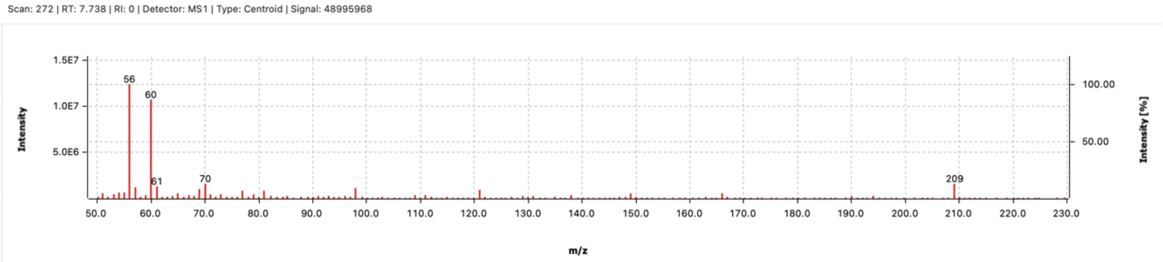

Assignment of Stereochemistry: A commercial solution of 3-Me cyclohexylamine (mixture of diastereomers) was purchased and analyzed by 1H NMR (top spectra). Comparison to literature data showed the mixture was ~3:1 *trans* to *cis*. The products of the transaminase reactions with 3-trifluoromethylcyclohexanone were analyzed by 1H NMR (middle and bottom spectra) and displayed similar signals to those present in the 3Me solution, in which the proton ipso to the supposed *trans* amine had a higher chemical shift and smaller coupling constant than the same proton in the *cis* amine. By analogy, we assigned the *trans* isomer and the *cis* isomer of 3-trifluoromethylcyclohexanamine. The R and S enantiomers were assigned by analogy to the 3-Me derivatization GC data in which the R products were closely clustered in the middle of the two S products.

Assignment of Stereochemistry using the ^{1}H NMR signal of ipso hydrogen as observed in the ^{1}H crude reaction: (C_6D_6). Top: Commercial 3-Me. Middle: Kinetic (*trans*). Bottom: Thermodynamic (*cis*)

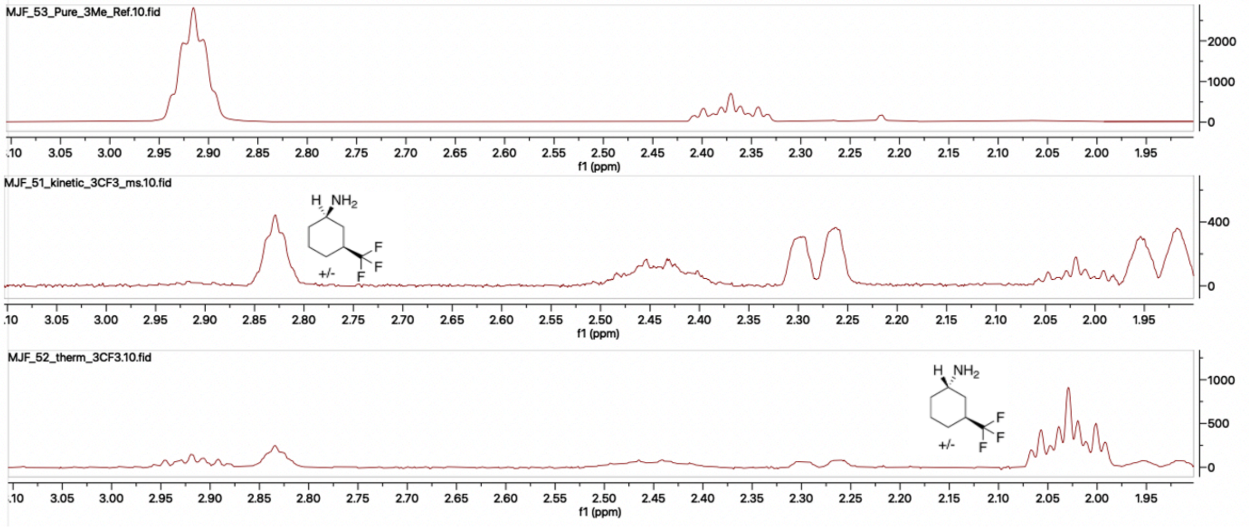

(*S*) 3-Trifluoromethylcyclohexanone *(S)*-12A

Derivatized Reactions

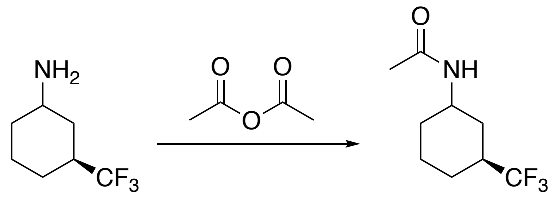

GC Data: Chiral column. Top. Kinetic condition, *trans* favoring: 30 eq alanine, WT without pyridoxine hydrochloride, 6 hours. Bottom. Thermodynamic condition, *cis* favoring: 2.5 eq alanine, 0.1 eq PLP, WT with pyridoxine hydrochloride, 6 hours.

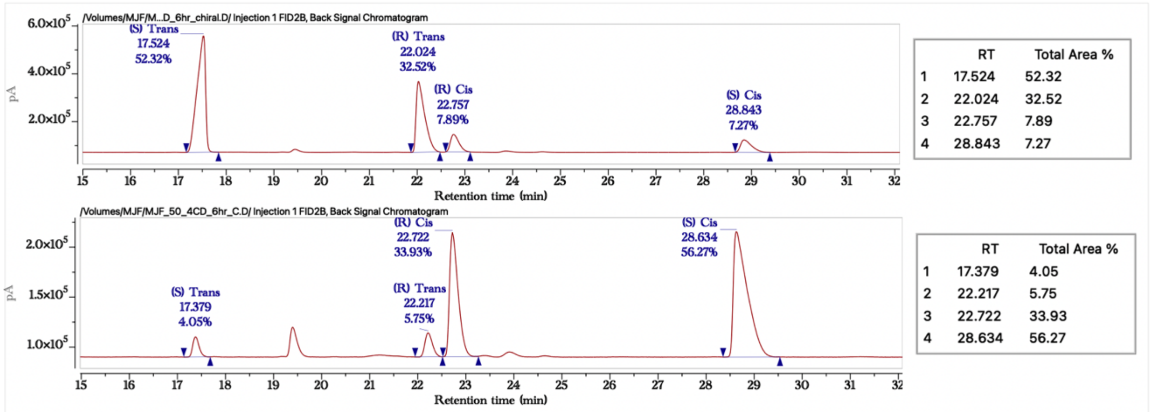

(*R*) 3-Trifluoromethylcyclohexanone *(R)*-12A

Derivatized Reactions

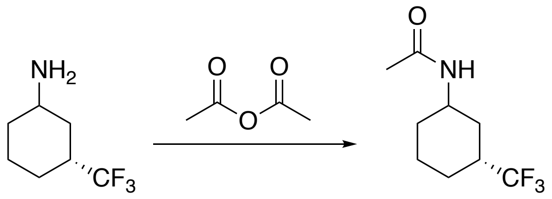

GC Data: Chiral column. Top. Kinetic condition, *trans* favoring: 30 eq alanine, WT without pyridoxine hydrochloride, 6 hours. Middle. Thermodynamic condition, *cis* favoring: 2.5 eq alanine, 0.1 eq PLP, WT with pyridoxine hydrochloride, 6 hours.

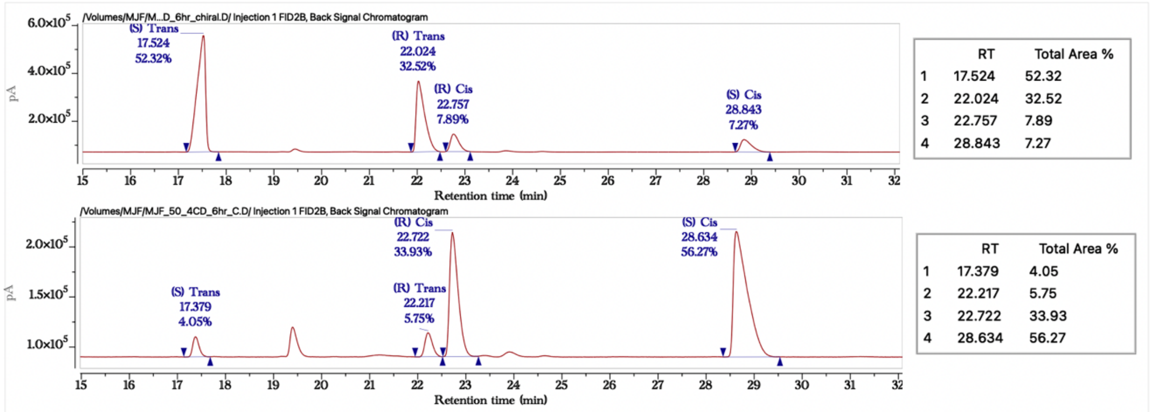

3-Ph Cyclohexanone (13)

GC Data: Kinetic (30 eq Alanine, WT, 6hr)

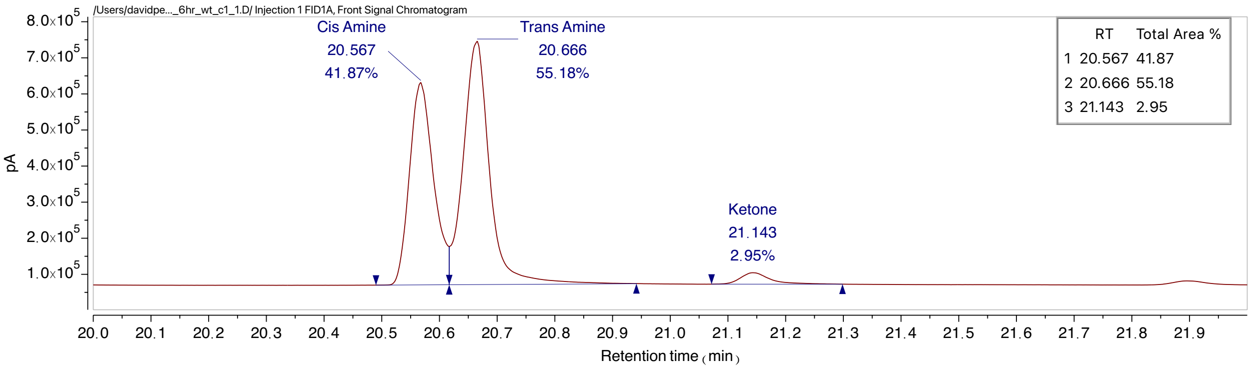

GC Data: Thermodynamic (I259V, double cells, 2.5 eq. Alanine, 0.1 eq. PLP, I259V, 72 hours)

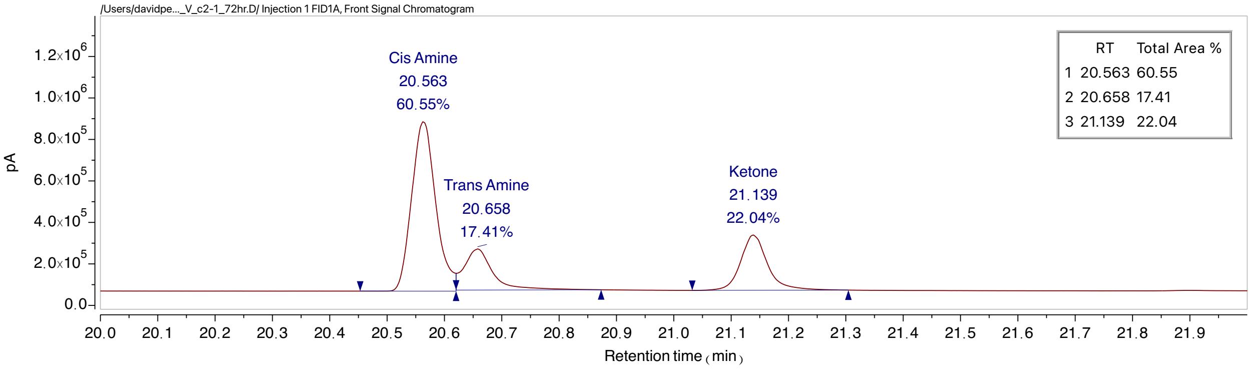

GC Data (Derivatized Reactions of 3-Ph cyclohexylamines): Top. Chemical reaction to produce racemic amines for derivatization to racemic amides (3-Ph Ketone, $NaBH(OAc)_3$, excess NH_4Cl, MeOH, mol sieves, 1 hr). Middle: Enzyme Reaction, Kinetic condition (*trans* favoring): 30 eq alanine, WT without pyridoxine hydrochloride, 24 hours. Bottom. Thermodynamic condition, *cis* favoring: 2.5 eq alanine, 0.1 eq PLP, I259V, 72 hours.

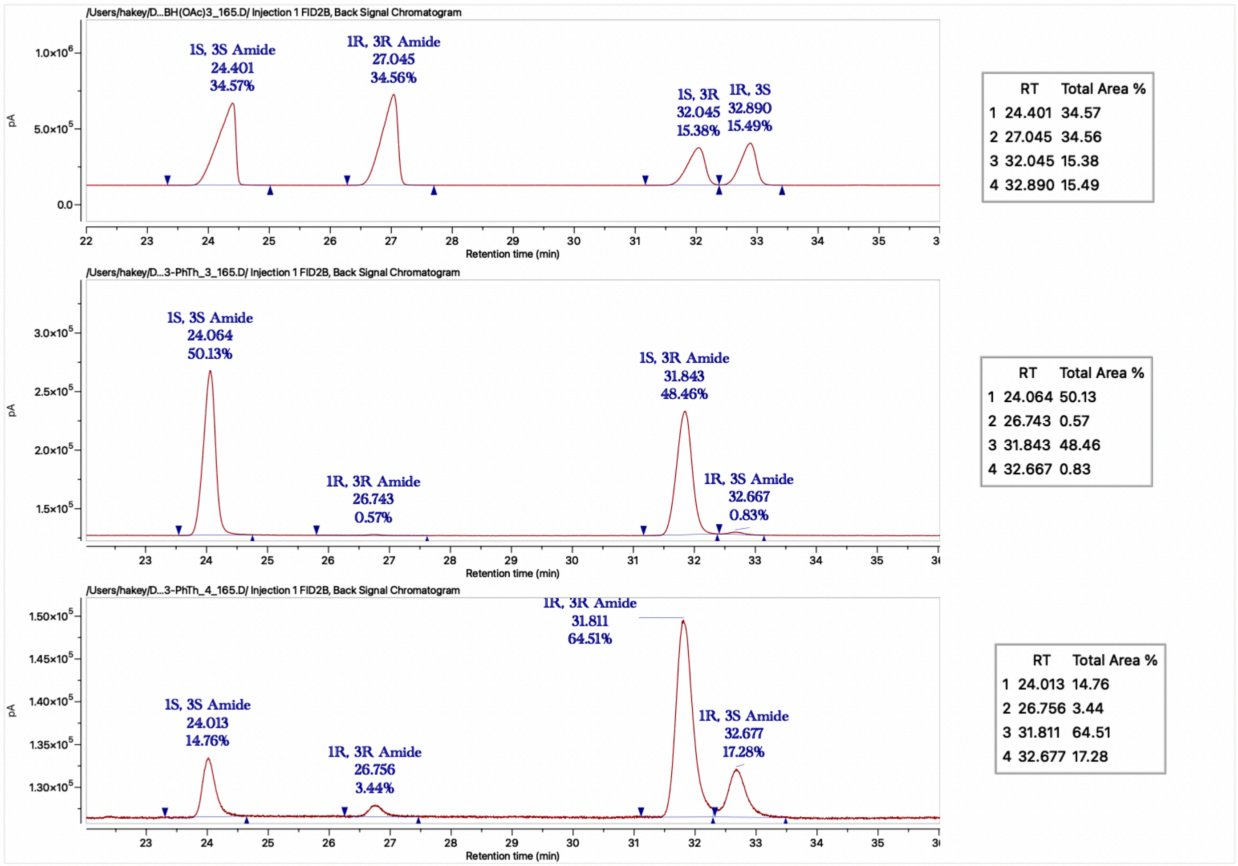

GC-MS Data

Kinetic (30 eq, WT)

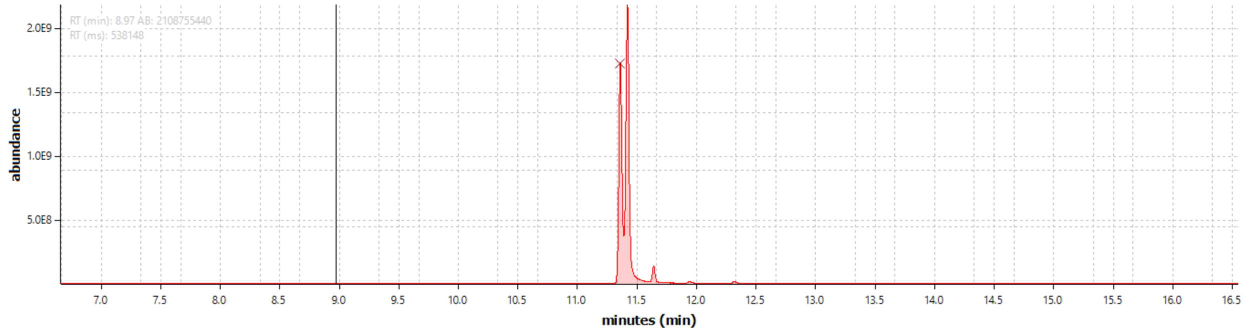

Peak 1:

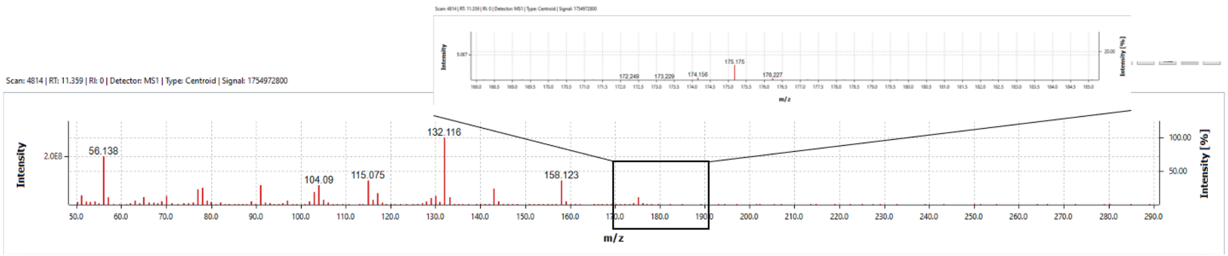

Peak 2:

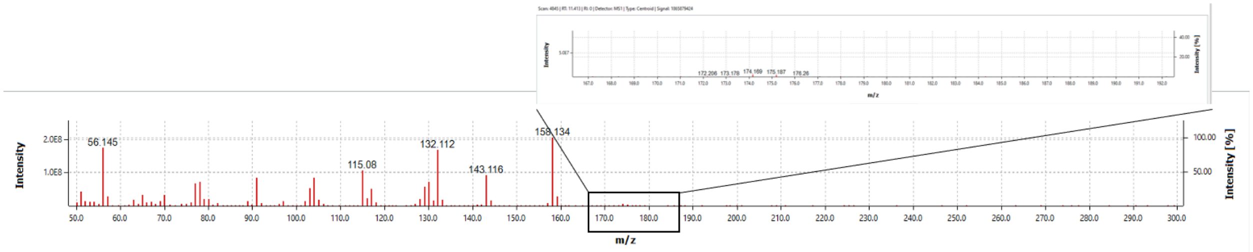

Peak 3:

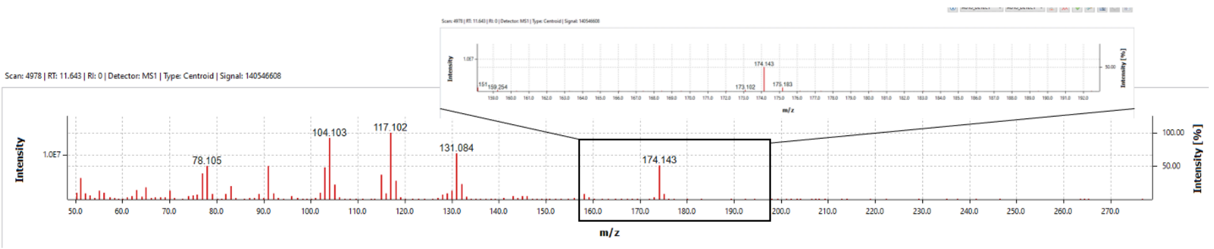

Thermodynamic (2.5 eq Alanine, 0.1 eq PLP, I259V, 72hr)

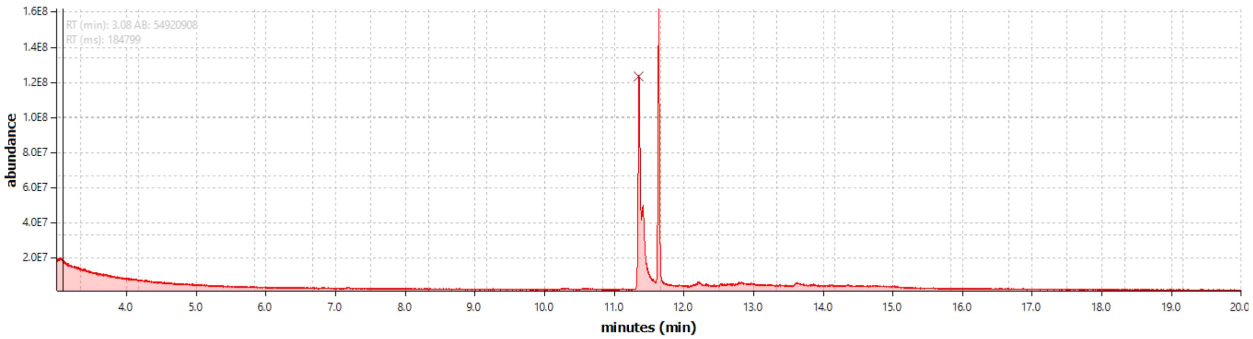

Peak 1:

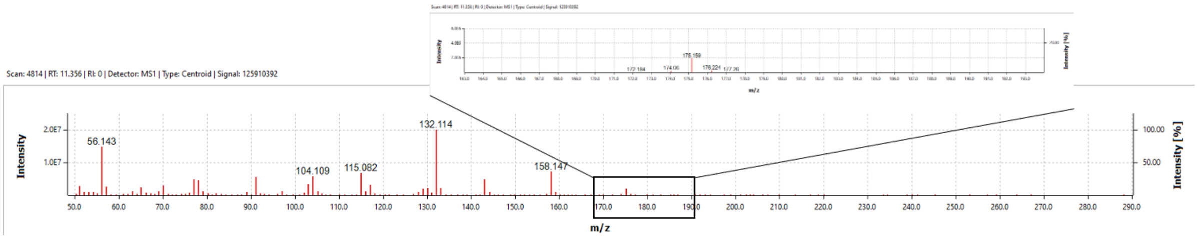

Peak 2:

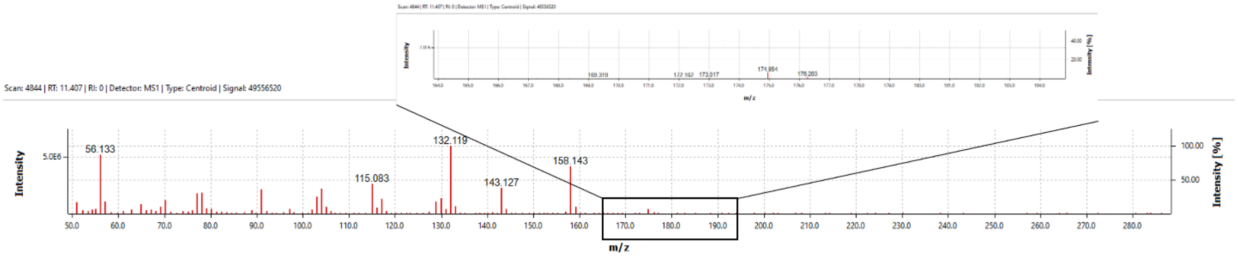

Peak 3:

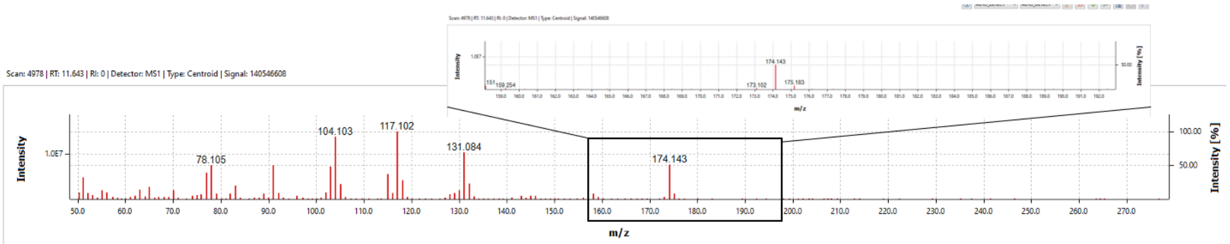

GC-MS Data of Derivatized Reaction- Kinetic Conditions

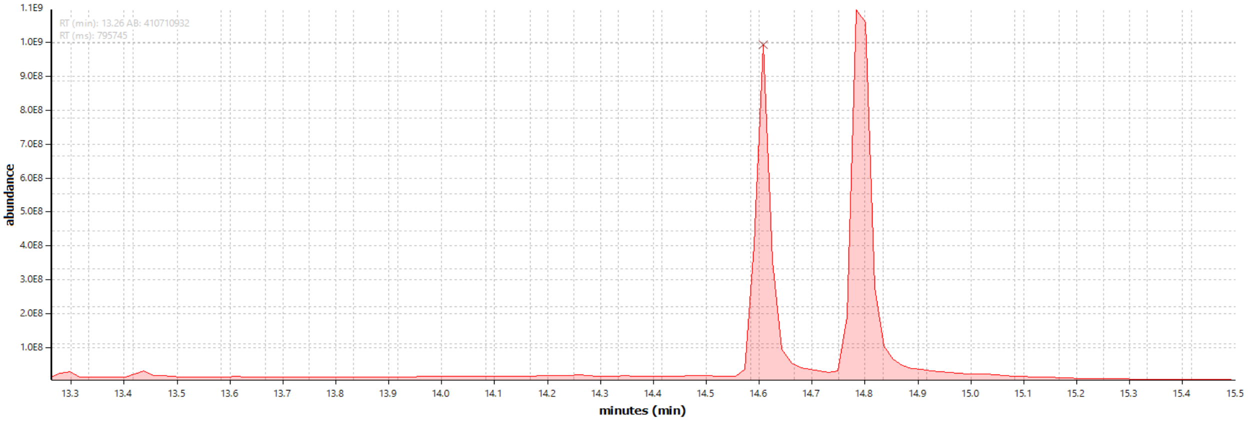

Peak 1- *Trans* Amide:

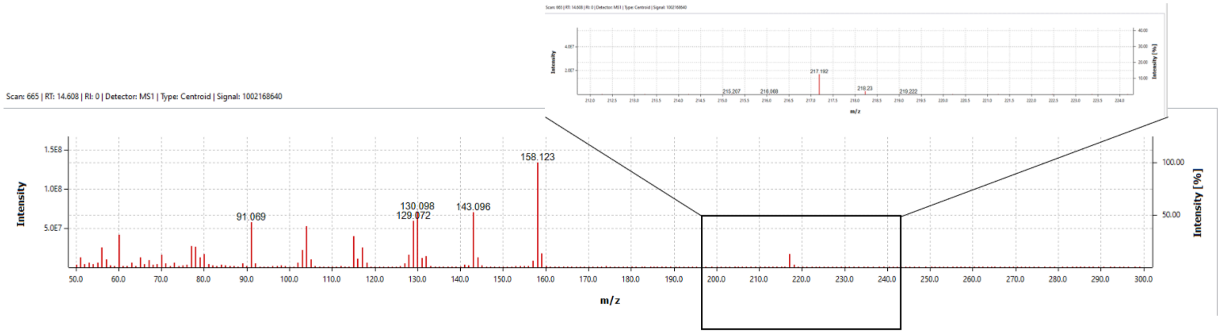

Peak 1- *Cis* Amide:

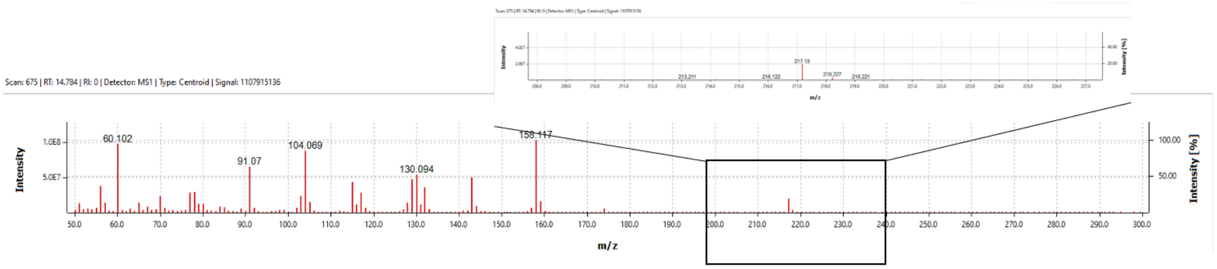

GC-MS Data of Derivatized Reaction- Thermodynamic Conditions

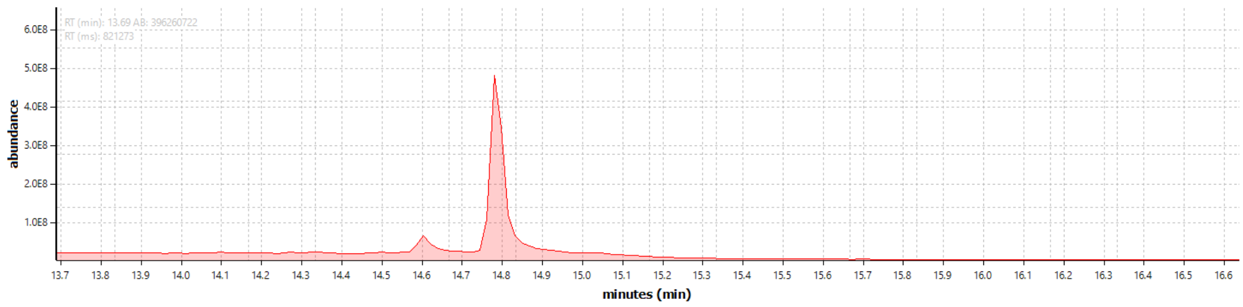

Peak 1- *Trans* Amide:

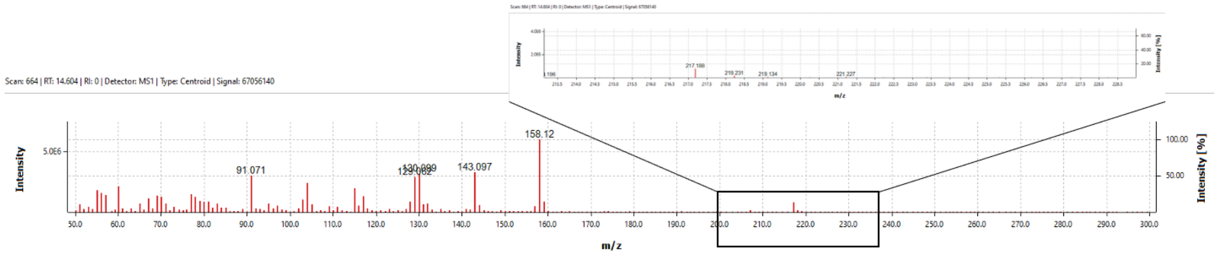

Peak 2- *Cis* Amide:

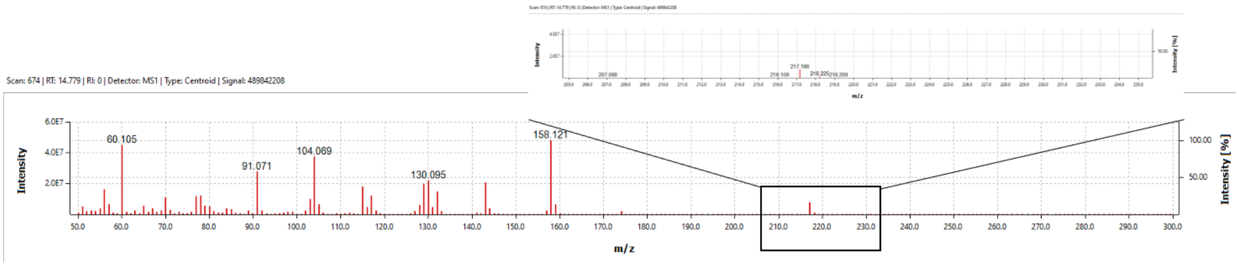

2-Methylcyclohexanone (14A)

GC Data: 1st Spectrum. Kinetic condition, *cis* favoring: 30 eq alanine, double WT cells, 24 hours. 2nd Spectrum. Optimized kinetic condition, *cis* favoring: 30 eq alanine, L56I-I259V cells without pyridoxine hydrochloride, 24 hours. 3rd Spectrum. Thermodynamic condition, *trans* favoring: 2.5 eq alanine, 0.1 eq PLP, WT without pyridoxine hydrochloride, 24 hours. 4th Spectrum. Optimized thermodynamic condition, *trans* favoring: 2.5 eq alanine, 0.1 eq PLP, I259V + L56V mutant, 20 hours. 5th Spectrum. Optimized thermodynamic condition, *trans* favoring: 2.5 eq alanine, 0.1 eq PLP, I259V + W57F mutant, 72 hours

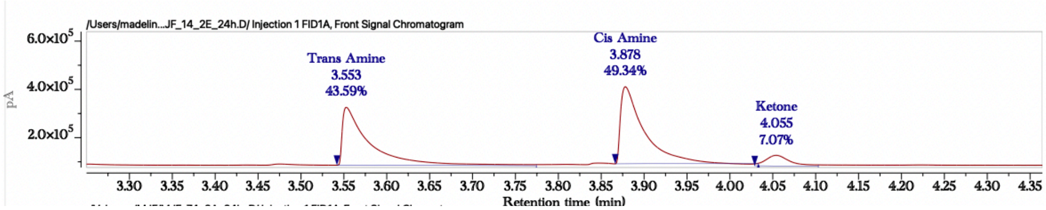

	RT	Total Area %
1	3.553	43.59
2	3.878	49.34
3	4.055	7.07

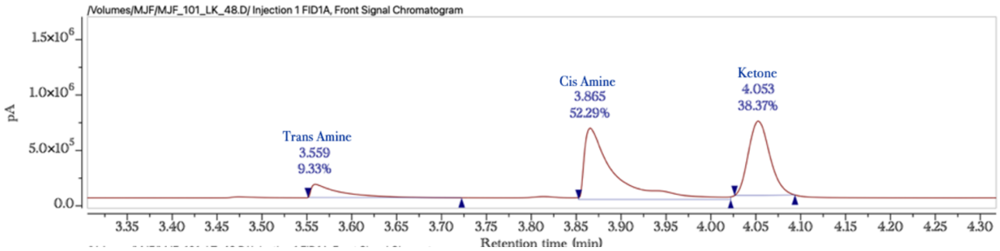

	RT	Total Area %
1	3.559	9.33
2	3.865	52.29
3	4.053	38.37

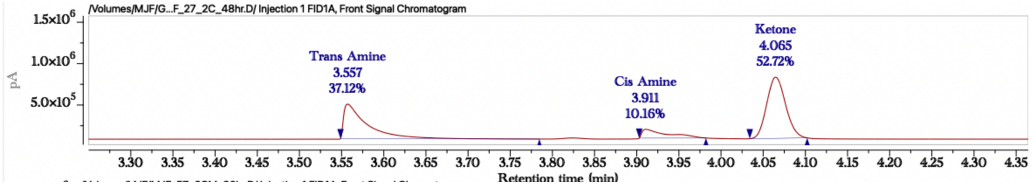

	RT	Total Area %
1	3.557	37.12
2	3.911	10.16
3	4.065	52.72

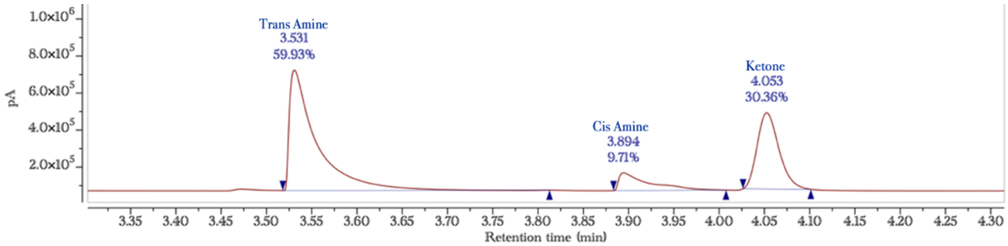

	RT	Total Area %
1	3.531	59.93
2	3.894	9.71
3	4.053	30.36

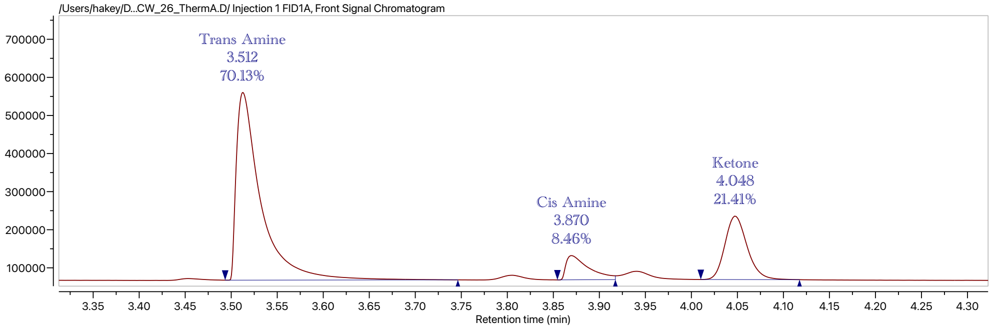

	RT	Total Area %
1	3.512	70.13
2	3.870	8.46
3	4.048	21.41

GC-MS Data: 1. Kinetic condition. 2. Thermodynamic condition.

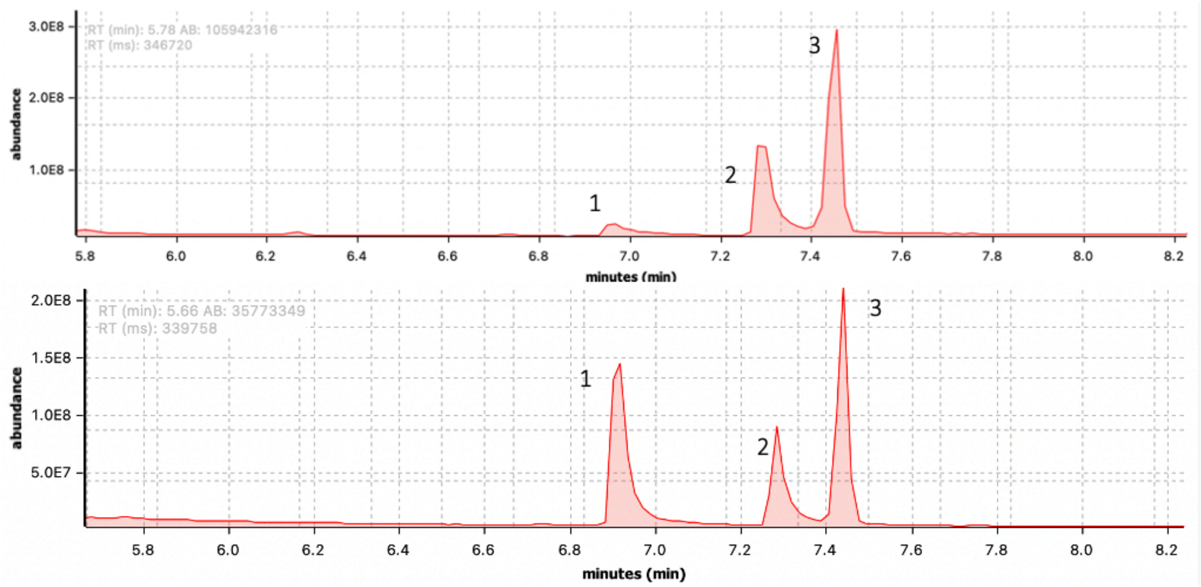

Peak 1. *Trans* Amine. MW = 113.

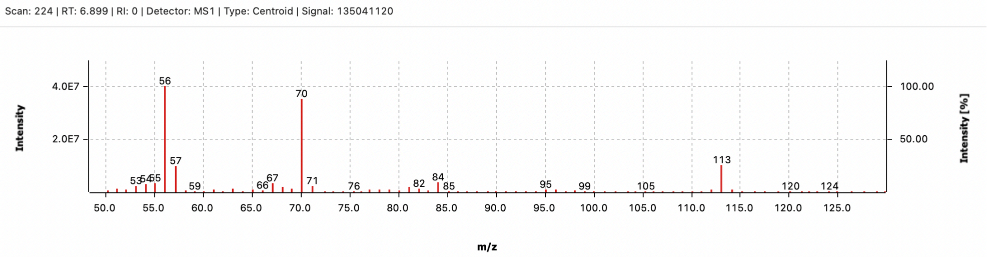

Peak 2. *Cis* Amine. MW = 113.

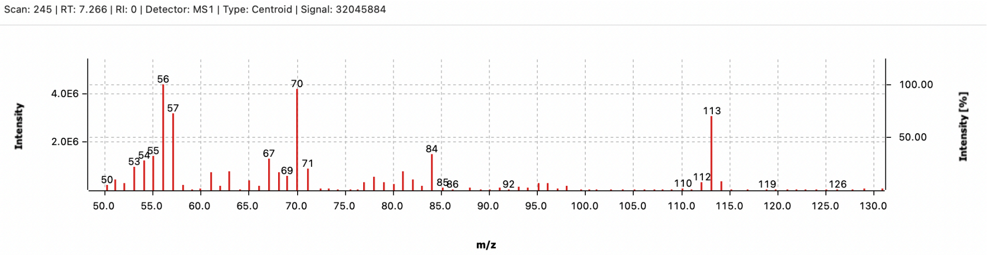

Peak 3. Ketone. MW = 112.

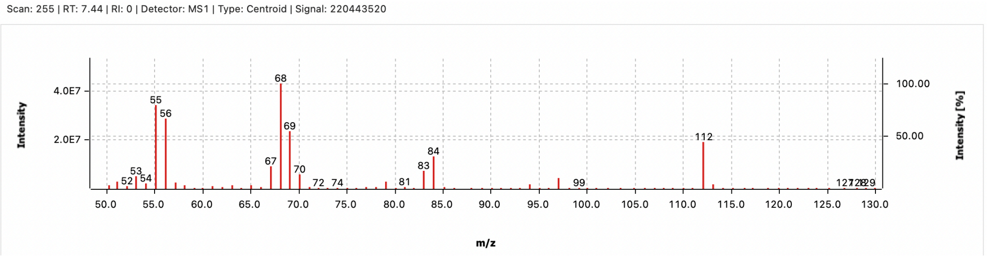

Derivatized Reactions:

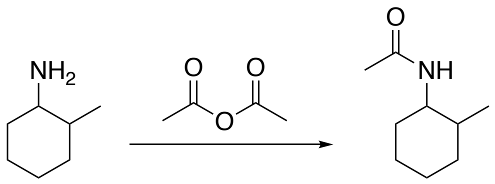

GC Data: Top. 2-Me Commercial Racemic mixture. Middle. Optimized kinetic condition, *cis* favoring: 30 eq alanine, L56I-I259V cells without pyridoxine hydrochloride, 24 hours. Bottom. Optimized thermodynamic condition, *trans* favoring: 2.5 eq alanine, 0.1 eq PLP, I259V + W57L mutant, 48 hours.

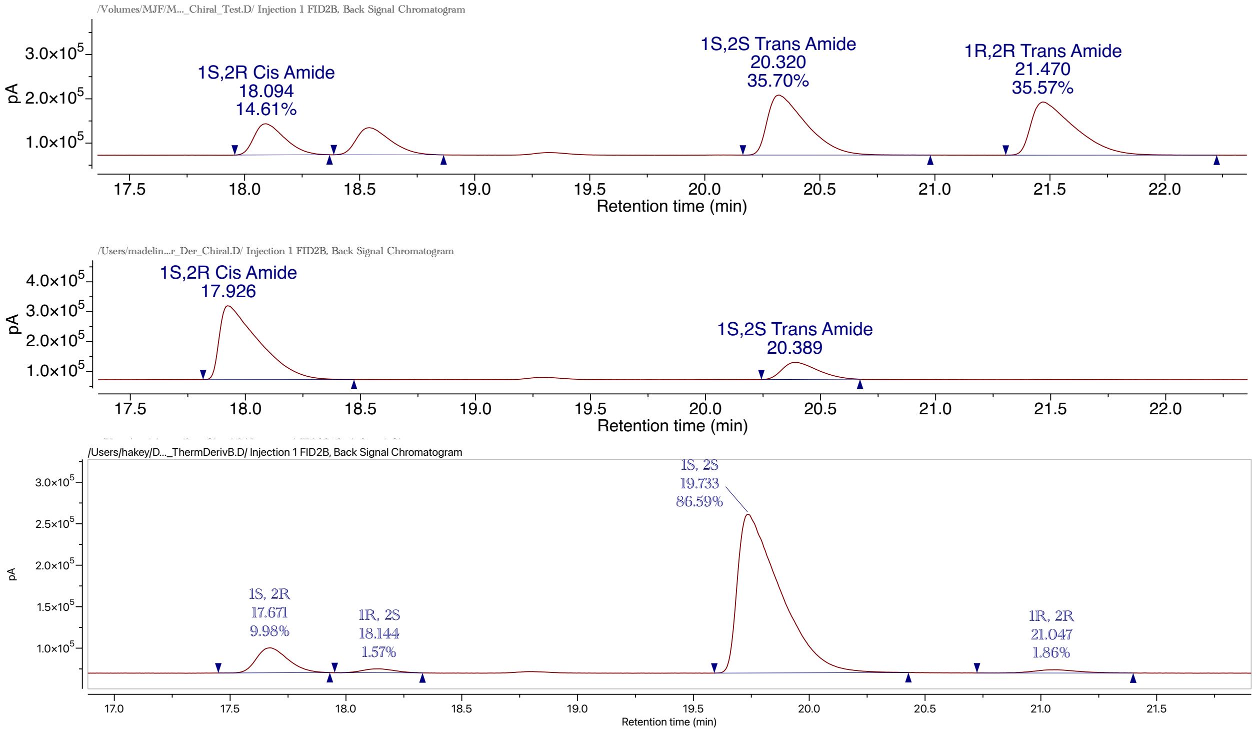

GC-MS Data: Top. Kinetic condition. 2 Bottom. Thermodynamic condition.

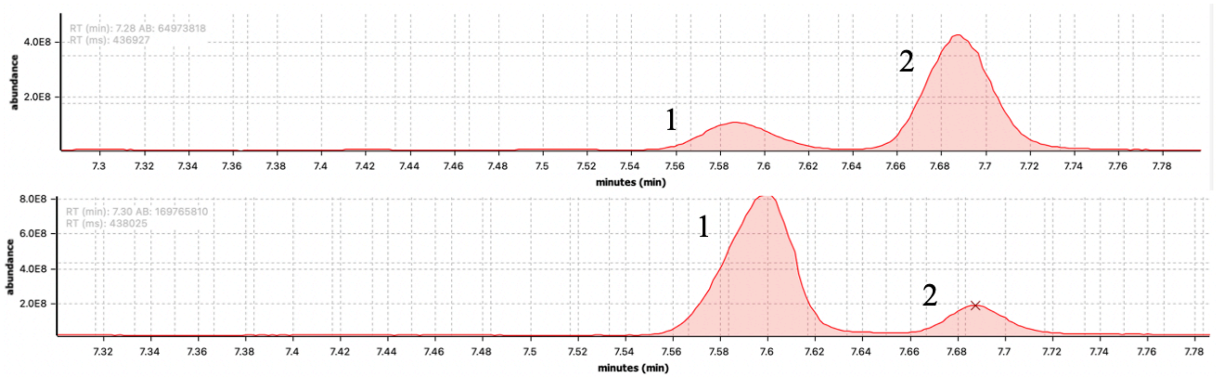

GC-MS Peak 1: *Cis* Amides (4ma, 4mb). MW = 155.

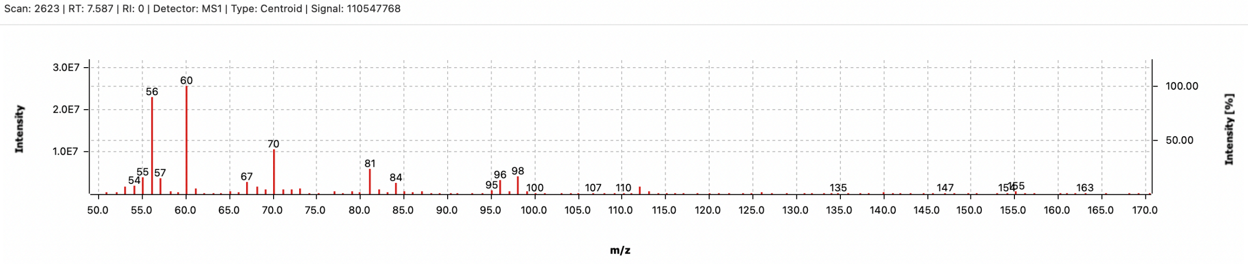

GC-MS Peak 2: *Trans* Amides (5ma, 5mb). MW = 155.

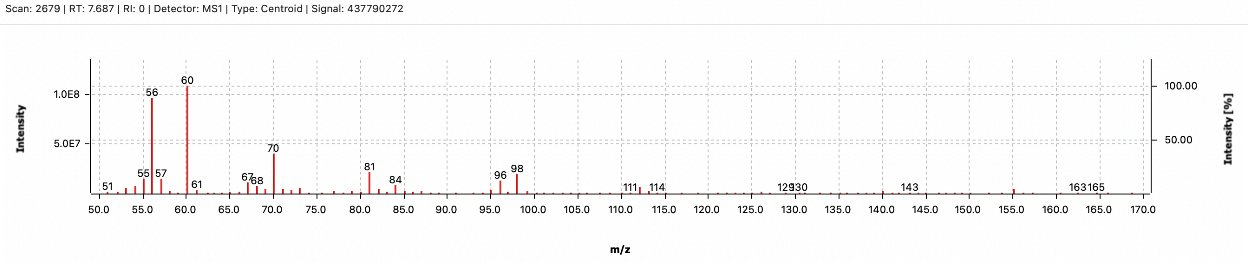

Assignment of Stereochemistry: A commercial solution of 2-Me cyclohexylamine (unreported dr) was purchased and analyzed by 1H NMR. Comparison to literature data showed the mixture was ~5:2 *trans* to cis. The sample was then analyzed by GC, producing two signals in a similar ratio, enabling assignment of the retention times of the *cis* and *trans* amines. The commercial amine was then derivatized with acetic anhydride to form a ~5:2 mixture of the corresponding amides. The derivatized product was analyzed by chiral GC to determine the retention times of the resulting *cis* and *trans* amides with concurrent separation of the four enantiomers. Identification of enantiomers was performed by comparison to 1*S*,2*R* and 1*R*,2*S* derivatized amine standards and literature reports.[32] Crude extracts from the reactions catalyzed by transaminases were either directed analyzed by 1H NMR and GC or derivatized to amides and analyzed with chiral GC in comparison to those samples from the commercially available amines.

Assignment of Stereochemistry using the ^{1}H NMR signal of ipso hydrogen as observed in the ^{1}H crude reaction: (C_6D_6). Top. 2-Me commercial racemic mixture. Middle. Kinetic condition. Bottom. Thermodynamic condition.

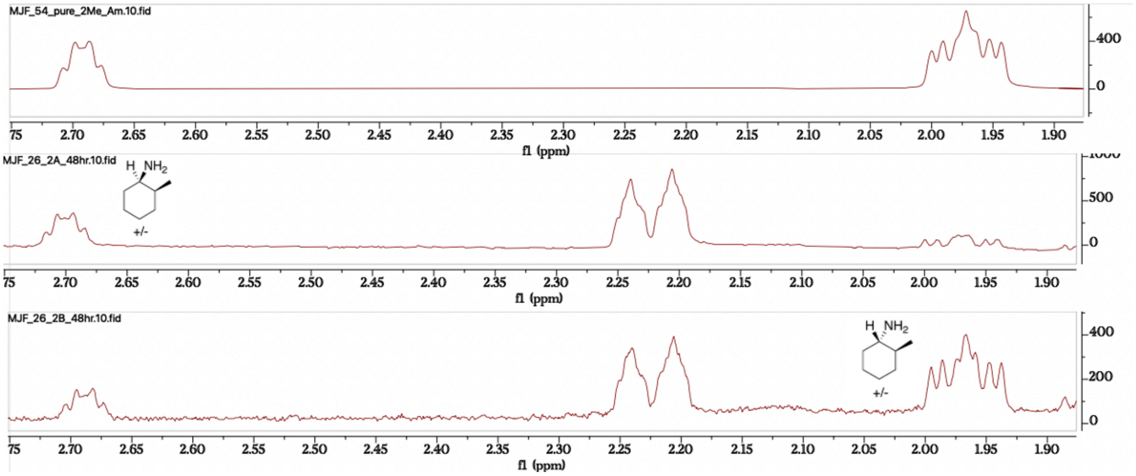

2-Allylcyclohexanone (16A)

GC Data: Top. Kinetic condition, *cis* favoring: 30 eq alanine, double WT cells without pyridoxine hydrochloride, 72 hours. Bottom. Optimized thermodynamic condition, *trans* favoring: 2.5 eq alanine, 0.1 eq PLP, double I259V + W57L mutant cells, 72 hours.

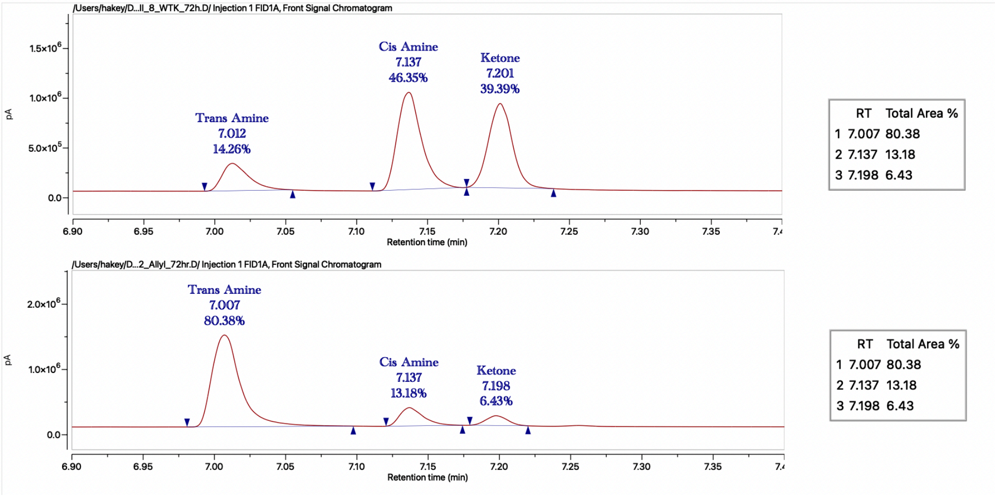

Chiral GC Data of Amide Derivatives: Stereochemistry assigned in analogy to 2-methyl substrate. Top. Chemical reaction to produce racemic amines for derivatization to racemic amides (2-allyl ketone, $NaBH(OAc)_3$, excess NH_4Cl, MeOH, mol sieves, 1 hr). Middle: Kinetic condition, *cis* favoring: 30 eq alanine, double WT cells without pyridoxine hydrochloride, 72 hours. Bottom: Optimized thermodynamic condition, *trans* favoring: 2.5 eq alanine, 0.1 eq PLP, double I259V + W57L mutant cells, 48 hours.

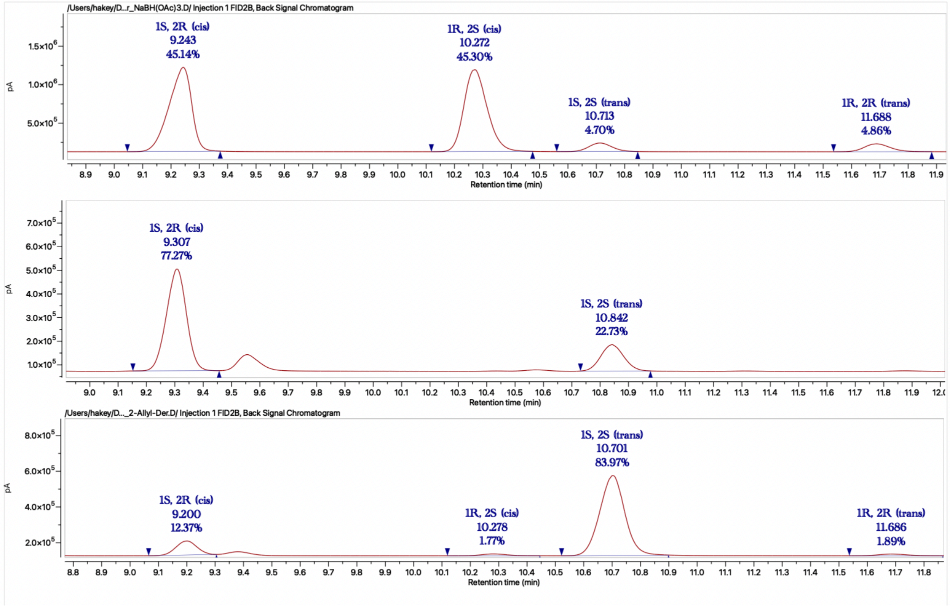

2-Ethyl Cyclohexanone (15A)

GC Data of Amines: Top. Kinetic condition, *cis* favoring: 30 eq alanine, WT cells with pyridoxine hydrochloride, 72 hours. Bottom. Optimized thermodynamic condition, *trans* favoring: 2.5 eq alanine, 0.1 eq PLP, double I259V + W57L mutant cells, 48 hours.

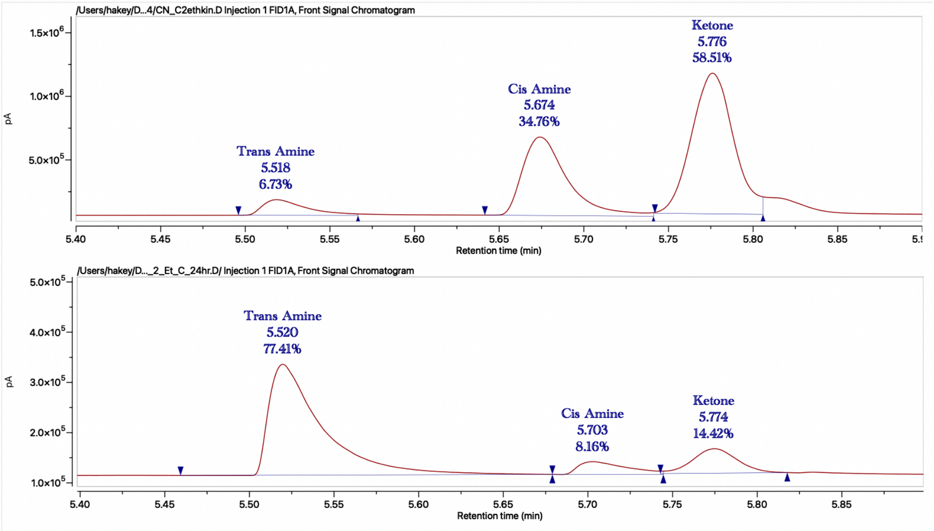

Chiral GC Data of Amine Derivatives: Stereochemistry assigned in analogy to 2-methyl substrate. Top: Chemical reaction to produce racemic amines for derivatization to racemic amides (2-ethyl ketone, $NaBH(OAc)_3$, excess NH_4Cl, MeOH, mol sieves, 1 hr). Middle. Kinetic condition, *cis* favoring: 30 eq alanine, WT cells with pyridoxine hydrochloride, 72 hours. Bottom. Optimized thermodynamic condition, *trans* favoring: 2.5 eq alanine, 0.1 eq PLP, double I259V + W57L mutant cells, 48 hours.

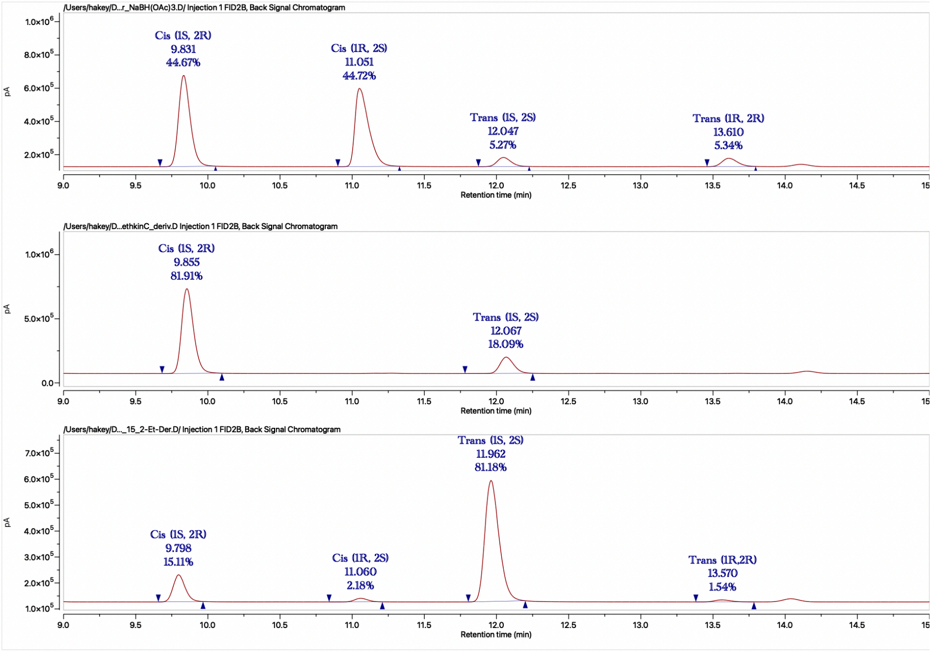

GC-MS Confirming Amine Formation

Kinetic (*cis* favoring) conditions

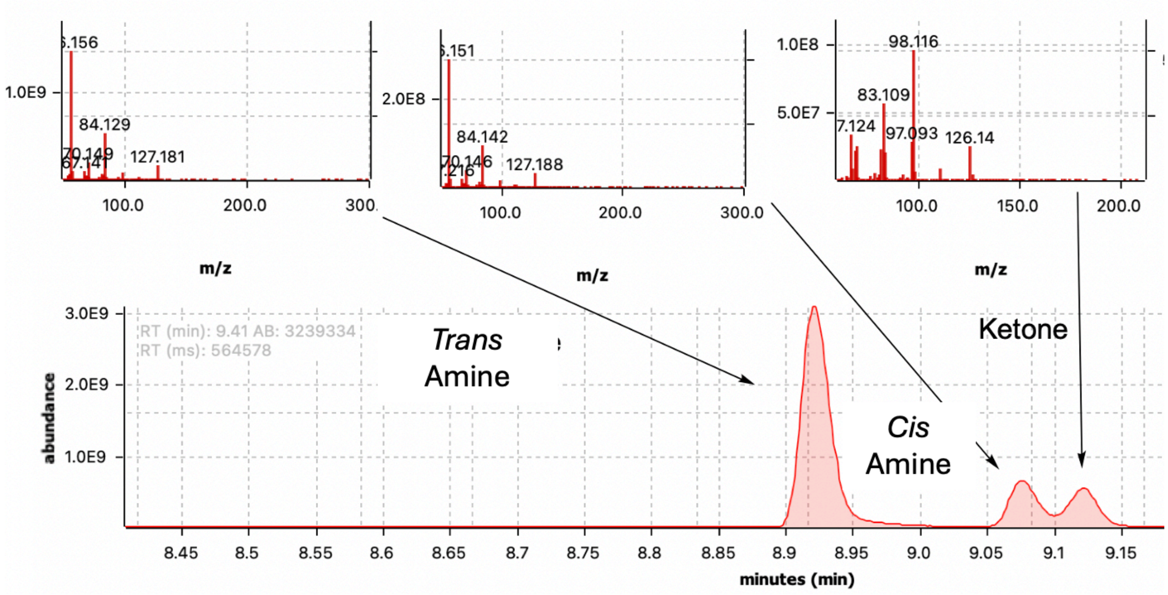

2-Phenylcyclohexanone (17A)

GC Data of Amines: Top: Kinetic condition: 30 eq alanine, 0.1 eq PLP, double I259V + W57G mutant cells, 48 hours. Bottom: Optimized thermodynamic condition, *trans* favoring: 30 eq alanine, 0.1 eq PLP, double I259V + W57G mutant cells, 48 hours.

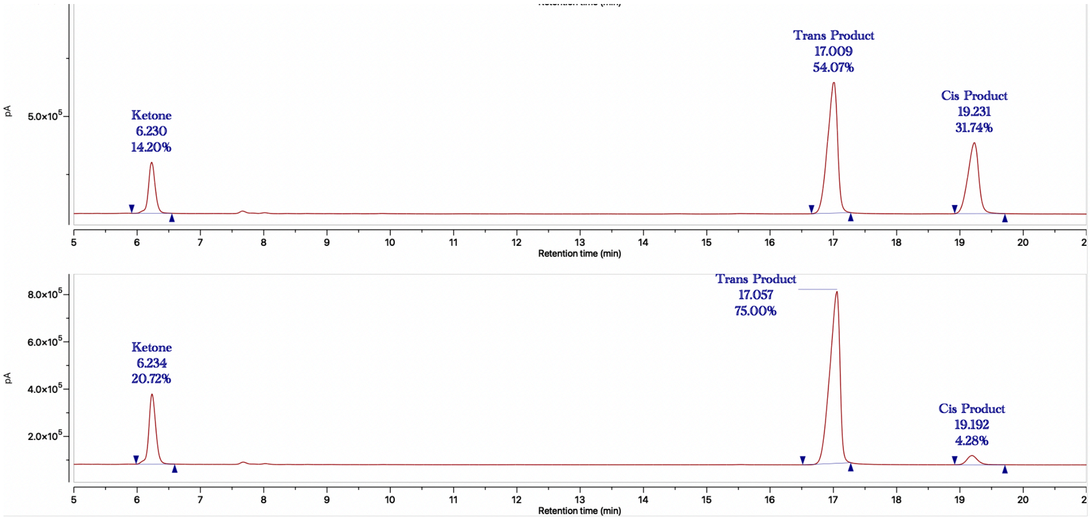

Chiral GC Data of Amine Derivatives: Stereochemistry assigned in analogy to 2-methyl substrate. Top: Chemical reaction to produce racemic amines for derivatization to racemic amides (2-Ph ketone, $NaBH(OAc)_3$, excess NH_4Cl, MeOH, mol sieves, 1 hr). Alcohol byproducts are also observed. Middle. Kinetic condition: 30 eq alanine, 0.1 eq PLP, double I259V + W57G mutant cells, 48 hours. Bottom. Optimized thermodynamic condition, *trans* favoring: 30 eq alanine, 0.1 eq PLP, double I259V + W57G mutant cells, 48 hours.

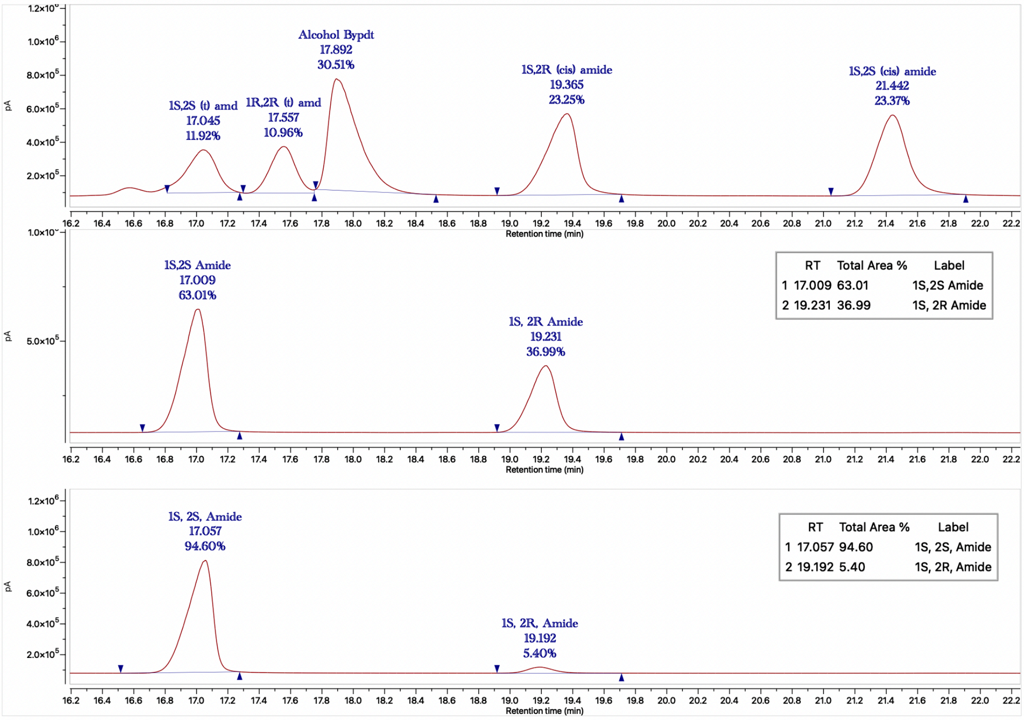

GC-MS Confirming Amine Formation

Kinetic (*cis* favoring) conditions

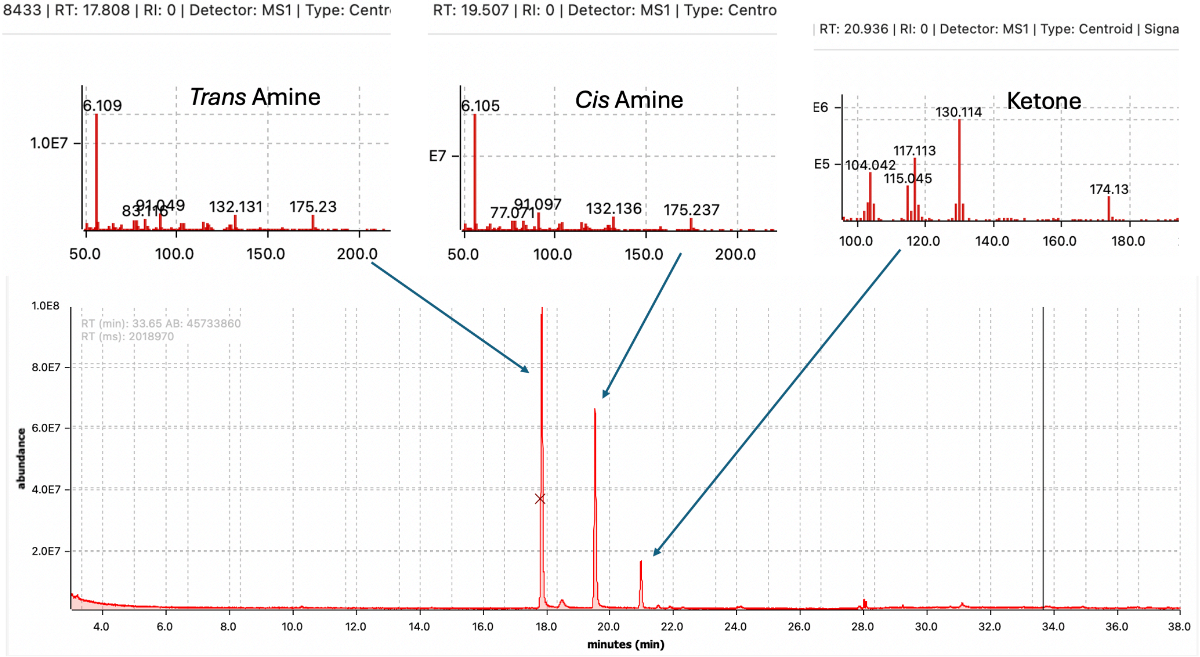

VIII. Characterization of Products from Representative Reactions Conducted on Preparative Scale

Reaction Protocol:

Kinetic Conditions:
Reactions were conducted in 100 or 250 mL round bottom flasks in a shaking incubator (0.8-1.0 inch orbit, 250 rpm, 37 ºC). Cells expressing WT ATA without PYP (160 grams/L in 100 mM NaPi, pH = 8.0) were thawed in a room temperature water bath and 8.75 mL were decanted into the round bottom flask. Additional NaPi buffer (17.5 mL) was then added to the round bottom flask, followed by aq. alanine (1M, 7.5 mL, 735 mg, 30 eq). The ketone (1M in DMSO, 250 uL, 0.250 mmol) was added in two portions and the reaction flask was briefly swirled between additions. The reaction flask was covered with an air permeable Kim wipe secured with tape around the neck of the flask and placed the aforementioned shaking incubator for 1-3 hours. Samples of the reaction (100 uL) were removed and analyzed by GC to monitor the progress of the reaction. Upon completion of the reaction, 1.5 mL of 10 M NaOH were added and the reaction was divided into two 40 mL glass vials. Each half of the reaction was extracted with diethyl ether (3 x 15 mL), and the amine from the combined organic extracts was then extracted with 0.1 M HCl (3 x 50 mL). The aqueous extract containing the amine was then basified with 3 mL of 10 M NaOH and extracted with diethyl ether (3 x 50 mL). The combined organic extracts were dried with $MgSO_4$, filtered, and concentrated under reduced pressure. Reactions on 1 mmol scale were conducted in proportional way to the described method for 0.25 mmol scale reactions, except that a total of 20 additional mL of buffer was added and the combined organic extracts from the first extraction were concentrated to approximately 100 mL before proceeding with further extractions in the purification process.

Thermodynamic Conditions:
Reactions were conducted in 100 or 250 mL round bottom flasks in a shaking incubator (0.8-1.0 inch orbit, 250 rpm, 37 ºC). Cells expressing Vf-ATA with I259V with PYP (160 grams/L in 100 mM NaPi, pH = 8.0) were thawed in a room temperature water bath and 17.5 mL were decanted into the round bottom flask. Additional NaPi buffer (10 mL) was then added to the round bottom flask, followed by aq. alanine (1M, 0.625 mL, 61 mg, 2.5 eq) and PLP (10 mM, 2.5 mL, 6.18 mg, 0.1 eq). The ketone (1M in DMSO, 250 uL, 0.250 mmol) was added in two portions and the reaction flask was briefly swirled between additions. The reaction flask was covered with an air permeable Kim wipe secured with tape around the neck of the flask and placed the aforementioned shaking incubator for 18-24 hours. Samples of the reaction (100 uL) were removed and analyzed by GC to monitor the progress of the reaction. Upon completion of the reaction, 1.5 mL of 10 M NaOH were added and the reaction was divided into two 40 mL glass vials. Each half of the reaction was extracted with diethyl ether (3 x 15 mL), and the amine from the combined organic extracts was then extracted with 0.1 M HCl (3 x 50 mL). The aqueous extract containing the amine was then basified with 3 mL of 10 M NaOH and extracted with diethyl ether (3 x 50 mL). The combined organic extracts were dried with $MgSO_4$, filtered, and concentrated under reduced pressure.

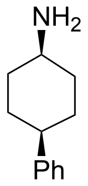

Cis-4-phenylcyclohexylamine (**8B**): 43.5 mg (0.250 mmol) ketone used in reaction. 34.1 mg amine isolated (white solid). 78% isolated yield, >40 : 1 dr (GC-FID). Compound previously reported[1]

mmol scale reaction: 174 mg (1.00 mmol) ketone used in reaction. 136.1 mg amine isolated (white solid). 78% isolated yield, >40 : 1 dr (GC-FID).

GC-FID of purified amine (mmol scale). Found to contain 1.3% residual ketone.

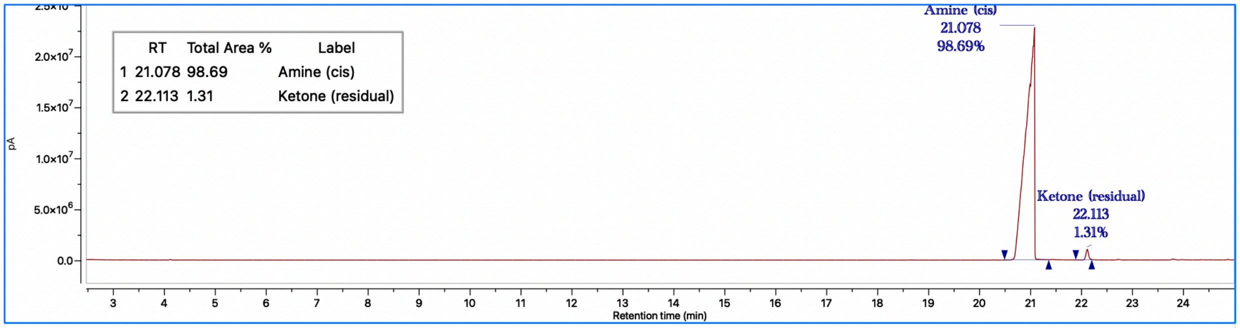

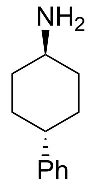

Trans-4-phenylcyclohexylamine (**8C**): 43.5 mg (0.250 mmol) ketone used in reaction. 28.3 mg amine isolated (white solid). 65% isolated yield, 14:1 dr (by NMR). Compound previously reported[2]

GC-FID of Purified Amine (imperfect separation)

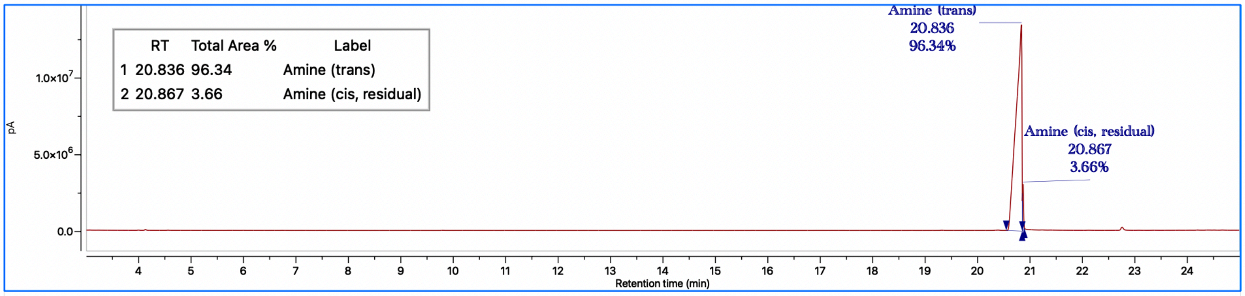

Alternative determination of diastereomeric ratio by NMR

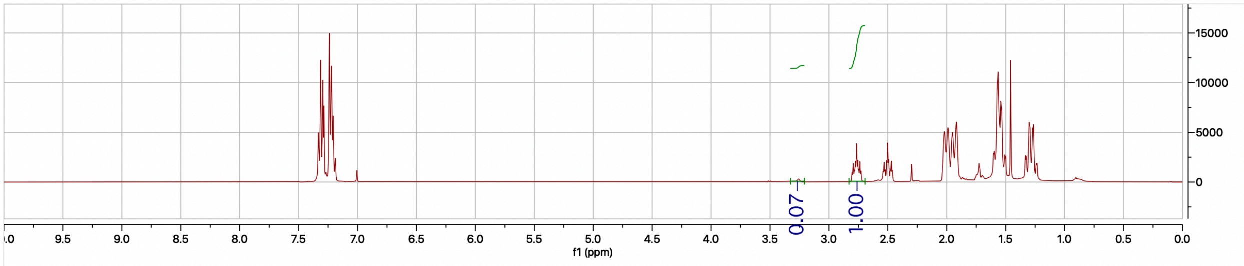

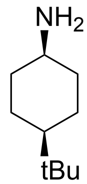

Cis-4-*tert*-butylcyclohexylamine (**7B**): 38.5 mg ketone used in reaction. 28.1 mg amine isolated (white solid). 73% isolated yield (>40:1 dr, GC-FID). Compound previously reported[1]

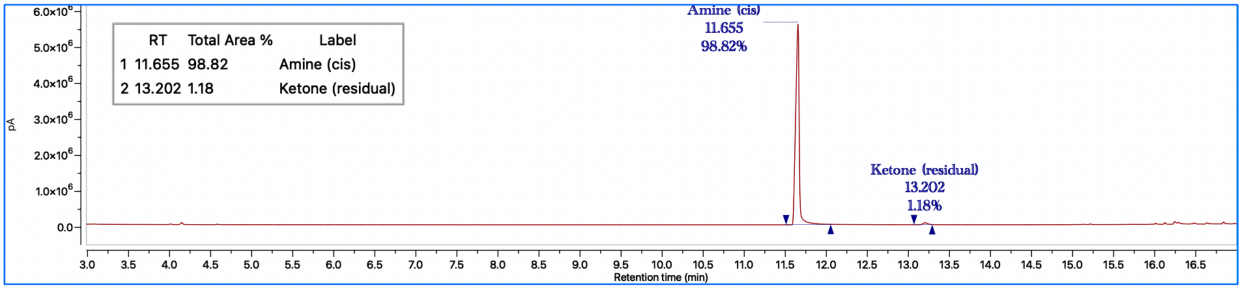

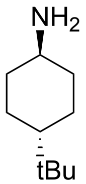

Trans-4-*tert*-butylcyclohexylamine (**7C**): 38.5 mg ketone used in reaction. 29.3 mg amine isolated (white solid). 76% isolated yield, 32:1 dr (GC-FID). Compound previously reported[3]

GC-FID of the Purified Amine

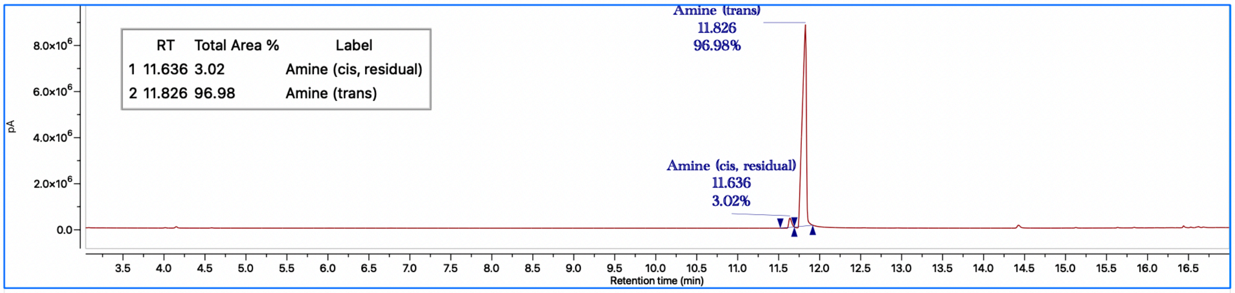

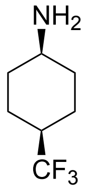

Cis-4-trifluoromethylcyclohexylamine (**9B**): 41.5 mg (0.250 mmol) ketone used in reaction. 23.2 mg amine isolated (colorless liquid). 56% isolated yield (6 : 1 dr). Compound previously reported[4]

mmol scale: 166 mg (1.00 mmol) ketone used in reaction. 131.3 mg amine isolated (colorless liquid). 79% isolated yield (6 : 1 dr).

GC-FID of Purified Amine:

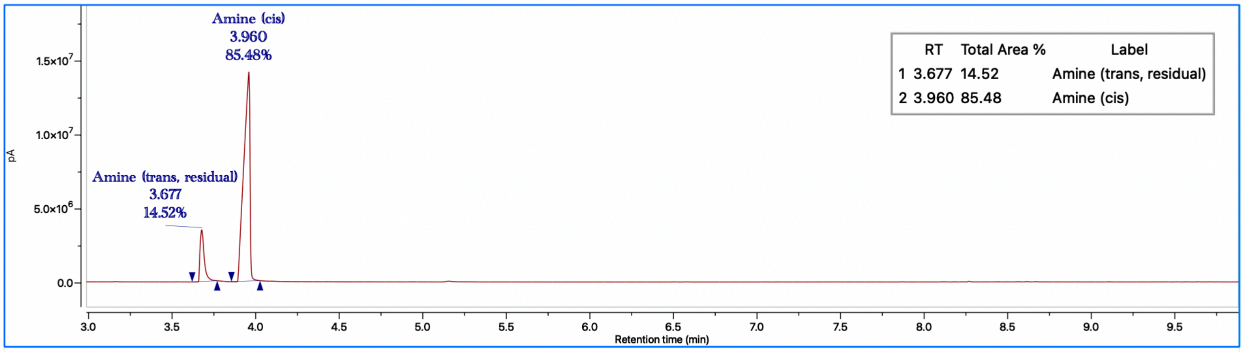

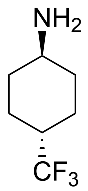

Trans-4-trifluoromethylcyclohexylamine (**9C**): 41.5 mg (0.250 mmol) ketone used in reaction. 19.4 mg amine isolated (colorless liquid). 46% isolated yield, 12: 1 dr (GC-FID). Compound previously reported[4]

GC-FID of Purified Amine:

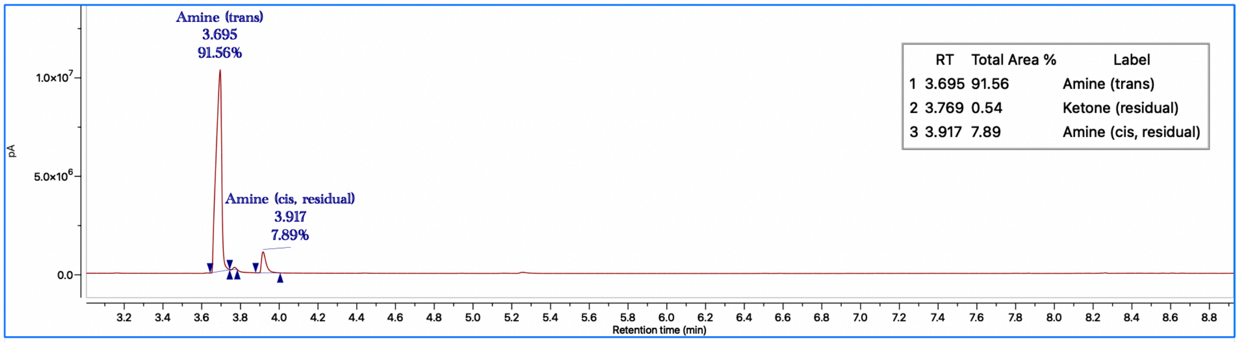

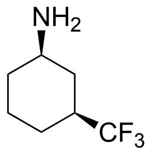

Cis-3-trifluoromethylcyclohexylamine (**12C, mix of enantiomers**): 41.5 mg (0.250 mmol) ketone used in reaction. 24.0 mg amine isolated (colorless liquid). 57% isolated yield (14:1 dr, GC-FID). Compound previously reported[5]

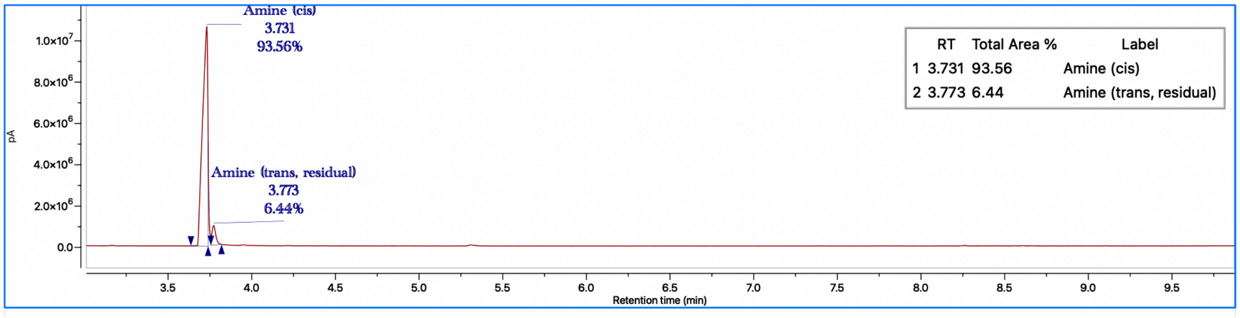

Determination of diastereomeric ratio by 19F NMR:

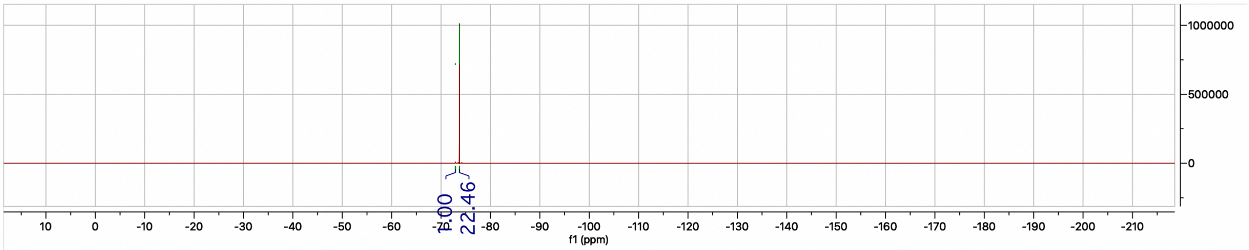

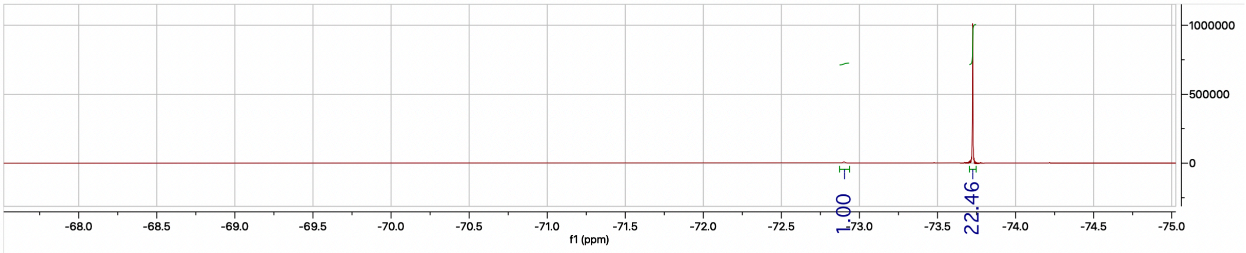

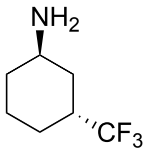

Trans-3-trifluoromethylcyclohexylamine (**19B**, mixture of enantiomers): 41.5 mg (0.250 mmol) ketone used in reaction. 20.2 mg amine isolated (colorless liquid). 48% isolated yield (8:1 dr). Compound previously reported.[5]

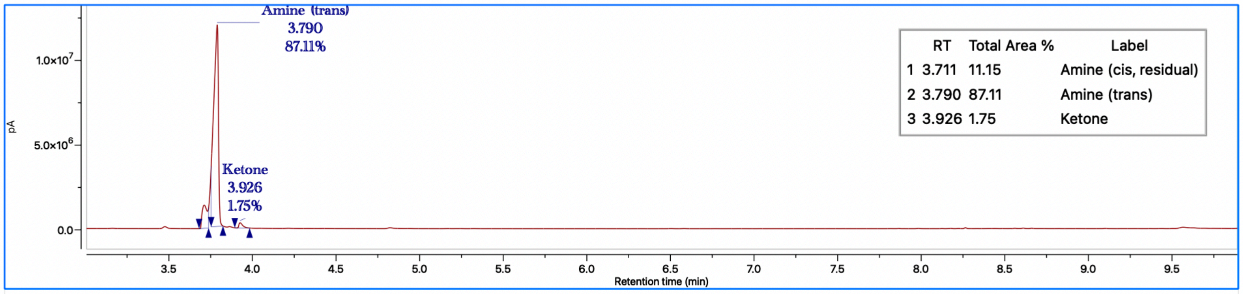

Determination of diastereomeric ratio by ^{19}F NMR:

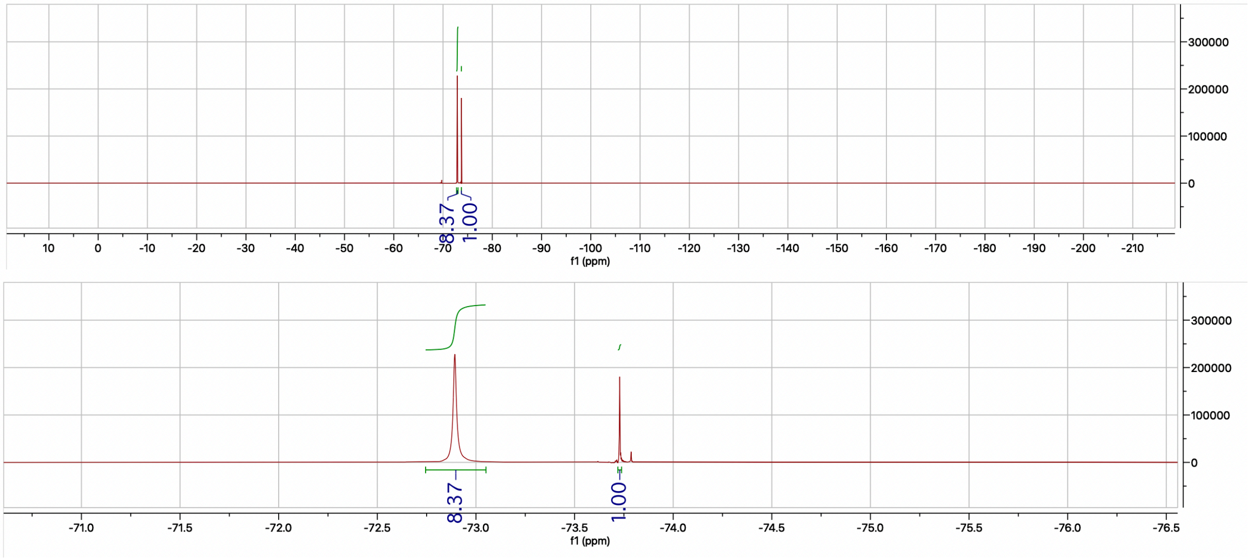

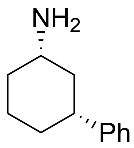

Cis-3-phenylcyclohexylamine (**13C**): 43.5 mg (0.250 mmol) ketone used in reaction. 30.0 mg amine isolated (white solid). 69% isolated yield (7:1 dr). Compound previously reported[6]

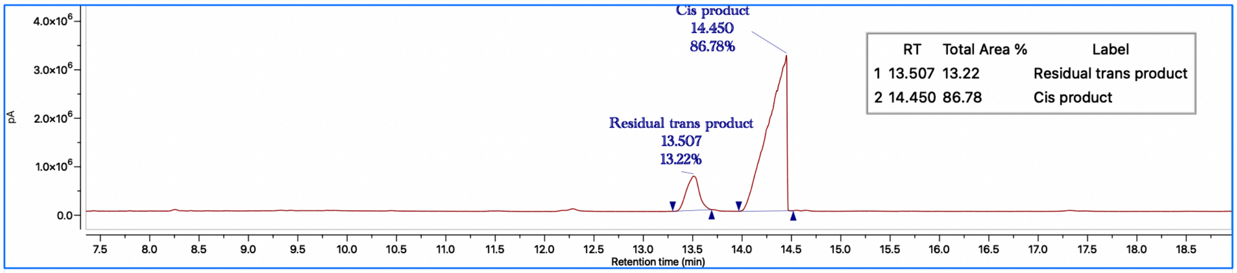

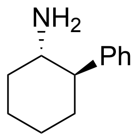

Trans-2-phenylcyclohexylamine (**17C**): 43.5 mg ketone used in reaction. 26.9 mg amine isolated (white solid). 62% isolated yield (40 : 1 dr, GC-FID). Compound previously reported[7]

GC-FID of Purified Product

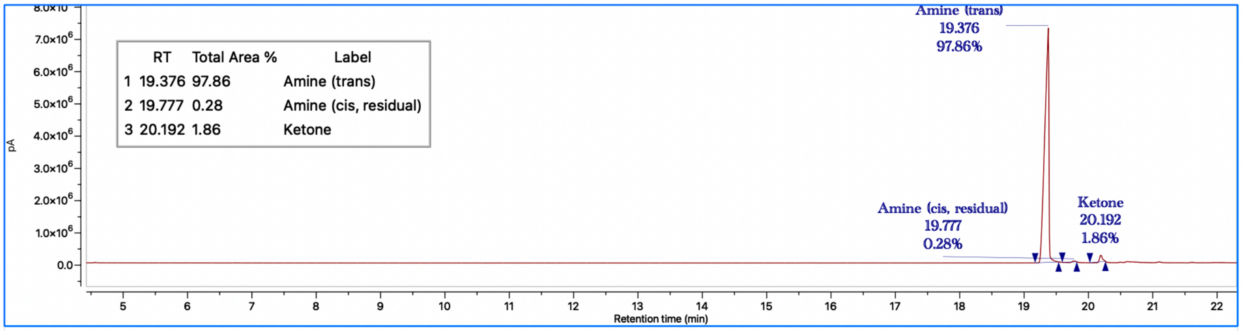

Chiral GC-FID of the Derivatized Product

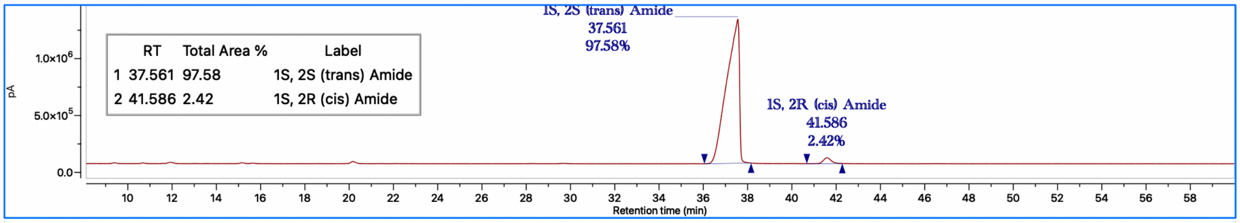

IX. References

(1) Tanaka, K.; Miki, T.; Murata, K.; Yamaguchi, A.; Kayaki, Y.; Kuwata, S.; Ikariya, T.; Watanabe, M. Reductive Amination of Ketonic Compounds Catalyzed by Cp*Ir(III) Complexes Bearing a Picolinamidato Ligand. *J. Org. Chem.* **2019**, *84* (17), 10962–10977. https://doi.org/10.1021/acs.joc.9b01565.

(2) Gawaskar, S.; Schepmann, D.; Bonifazi, A.; Wünsch, B. Synthesis, GluN2B Affinity and Selectivity of Benzo[7]Annulen-7-Amines. *Bioorg. Med. Chem.* **2014**, *22* (23), 6638–6646. https://doi.org/10.1016/j.bmc.2014.10.004.

(3) Fiorati, A.; Berglund, P.; Humble, M. S.; Tessaro, D. Application of Transaminases in a Disperse System for the Bioamination of Hydrophobic Substrates. *Adv. Synth. Catal.* **2020**, *362* (5), 1156–1166. https://doi.org/10.1002/adsc.201901434.

(4) Harrison, J. R.; Sarkar, S.; Hampton, S.; Riley, J.; Stojanovski, L.; Sahlberg, C.; Appelqvist, P.; Erath, J.; Mathan, V.; Rodriguez, A.; Kaiser, M.; Pacanowska, D. G.; Read, K. D.; Johansson, N. G.; Gilbert, I. H. Discovery and Optimization of a Compound Series Active against Trypanosoma Cruzi, the Causative Agent of Chagas Disease. *J. Med. Chem.* **2020**, *63* (6), 3066–3089. https://doi.org/10.1021/acs.jmedchem.9b01852.

(5) Alekseenko, A.; Denis, K.; Lukin, O.; Mykhailiuk, P.; Shishkin, O.; Pustovit, Y. An Improved Synthesis of 2-, 3-, and 4-(Trifluoromethyl)Cyclohexylamines. *Synthesis* **2012**, *44* (17), 2739–2742. https://doi.org/10.1055/s-0032-1316985.

(6) Llona-Minguez, S.; Mackay, S. P. Stereoselective Synthesis of Carbocyclic Analogues of the Nucleoside Q Precursor (PreQ0). *Beilstein J. Org. Chem.* **2014**, *10* (1), 1333–1338. https://doi.org/10.3762/bjoc.10.135.

(7) Cheng, X.; Yang, B.; Hu, X.; Xu, Q.; Lu, Z. Visible-Light-Promoted Metal-Free Aerobic Oxidation of Primary Amines to Acids and Lactones. *Chem. A Eur. J.* **2016**, *22* (49), 17566–17570. https://doi.org/10.1002/chem.201604440.
